# Network-Based Method for Identifying Co-Regeneration Genes in Bone, Dentin, Nerve and Vessel Tissues

**DOI:** 10.3390/genes8100252

**Published:** 2017-10-02

**Authors:** Lei Chen, Hongying Pan, Yu-Hang Zhang, Kaiyan Feng, XiangYin Kong, Tao Huang, Yu-Dong Cai

**Affiliations:** 1 School of Life Sciences, Shanghai University, Shanghai 200444, China; chen_lei1@163.com; 2 College of Information Engineering, Shanghai Maritime University, Shanghai 201306, China; 3 Department of Oral Medicine, Infection and Immunity, Harvard School of Dental Medicine, Harvard University, Boston, MA 02115, USA; hypan.harvard@gmail.com; 4 Department of Orthopedic Surgery, Brigham and Women’s Hospital, Harvard University, Boston, MA 02115, USA; 5 Institute of Health Sciences, Shanghai Institutes for Biological Sciences, Chinese Academy of Sciences, Shanghai 200031, China; zhangyh825@163.com; 6 Department of Computer Science, Guangdong AIB Polytechnic, Guangzhou 510507, China; addland@126.com

**Keywords:** co-regeneration, bone, dentin, nerve, vessel, protein–protein interaction

## Abstract

Bone and dental diseases are serious public health problems. Most current clinical treatments for these diseases can produce side effects. Regeneration is a promising therapy for bone and dental diseases, yielding natural tissue recovery with few side effects. Because soft tissues inside the bone and dentin are densely populated with nerves and vessels, the study of bone and dentin regeneration should also consider the co-regeneration of nerves and vessels. In this study, a network-based method to identify co-regeneration genes for bone, dentin, nerve and vessel was constructed based on an extensive network of protein–protein interactions. Three procedures were applied in the network-based method. The first procedure, searching, sought the shortest paths connecting regeneration genes of one tissue type with regeneration genes of other tissues, thereby extracting possible co-regeneration genes. The second procedure, testing, employed a permutation test to evaluate whether possible genes were false discoveries; these genes were excluded by the testing procedure. The last procedure, screening, employed two rules, the betweenness ratio rule and interaction score rule, to select the most essential genes. A total of seventeen genes were inferred by the method, which were deemed to contribute to co-regeneration of at least two tissues. All these seventeen genes were extensively discussed to validate the utility of the method.

## 1. Introduction

Bone and tooth are the only two hard organs in the human body and share many common features. These organs are both mineralized tissues composed of multipotent stem cells and calcified structures enclosing soft tissues that are full of nerves and vessels. Soft tissue inside the bone and enamel-dentin-cementum complex (EDCC) of teeth provide nutrition and sensors for the organ, shape the essential microenvironment for development and homeostasis of hard tissues, and build stem cell niches for stem cell differentiation in case of tissue repair and regeneration.

According to the International Osteoporosis Foundation and National Institutes of Health (NIH) reports, bone and dental diseases are considered serious public health concerns. In the USA, an estimated 1.5 million individuals suffer from bone fractures due to bone disease per year, while an additional 33.6 million are at risk of osteoporosis and its potential complications after 50 years of age [1]. Fractures in elderly populations represent a growing economic burden on the world health care system. In China alone, costs of osteoporosis treatment will rise from approximately USD 10 billion in 2010 to USD 25.4 billion in 2050. Due to an epidemic of dental diseases, 5% of adults from 20 to 64 years old lose all of their teeth. Moreover, among the remaining 95% of adults, the prevalence of tooth disease is as high as 92% (NIH reports). Due primarily to increasing life expectancy and global aging problems, there is an urgent need for research focused on new treatment targets to address bone and dental diseases (WHO reports).

Cell-mediated tissue repair and regeneration are regarded as promising therapies for bone and dental diseases. These therapies have been applied in human clinical trials with successful outcomes. However, many influencing factors may minimize the development of therapeutic regeneration, including scientific, technical, practical and regulatory obstacles and engineering technical bottlenecks [2]. The current leading theory in tissue engineering is to construct polymers of tissues or complex tunable tissue structures, whether they are naturally derived, synthetically produced or 3D-printed [3]. In this way, the compound tissue structure has the greatest potential to imitate the original dynamic tissue environment, recover cell-cell interactions and cell-environment interactions, and support compositional properties of various types of cells in cell-based therapies for injury, regeneration, and even drug delivery. However, one of the major challenges in this field is the limited understanding of the mechanisms of cell-mediated regeneration [2]. There is a call for genetic information and biological evidence of co-regeneration of multiple layers of tissues to form complex structures. The classic method to investigate this complicated biological process is through traditional biological experiments. Unfortunately, it is time-consuming and very expensive to discover a new key gene from hypothesis to validation. With the development of high-throughput-omics technologies and large-scale multi-omic data in public databases, building proper computational approaches is an alternative approach. Computational biological informatics makes it possible to uncover potential biological key factors based on previous experimental data, although it is challenging to apply suitable algorithms to these data correctly and efficiently. Therefore, in the present study, we sought to design a computational method that utilized validated data on regeneration in four tissues (bone, dentin, nerves and vessels) for the identification of co-regeneration genes for two tissues.

Recently, the classical shortest path graph algorithm has been widely adopted to identify genes with novel functions [4,5,6,7,8,9,10,11,12,13,14]. According to this method, the shortest paths connecting any two validated genes were calculated in a constructed network, and the inner nodes (genes) of these paths were extracted as candidate genes. In this study, we also used the shortest path algorithm as a basis for building the network-based method. To implement this method, we constructed a large network using protein–protein interaction (PPI) information retrieved from the Search Tool for the Retrieval of Interacting Genes/Proteins (STRING) [15]. The proposed method included three procedures: searching, testing and screening. Because we sought to identify co-regeneration genes of two tissues, the searching procedure used the shortest path algorithm to identify the shortest path connecting any regeneration gene of one tissue and any regeneration gene of the other tissue in this network. The inner nodes (genes) were extracted as possible co-regeneration genes for two tissues. The testing procedure identified false discoveries among candidate genes found in the searching procedure and excluded them. Finally, the screening procedure helped us to select the most essential genes. As a result, seventeen genes were yielded by the method, which were deemed to be co-regeneration genes of at least two tissues. Finally, all seventeen inferred genes were extensively analyzed based on their possible contribution to regeneration in at least two tissues. These results also validated the utility of the network-based method.

## 2. Materials and Methods

### 2.1. Regeneration Genes of Bone, Dentin, Nerve and Vessel

Gene profiles for regeneration of bone, dentin, vessels and nerves were collected from AmiGo [16] and the Regeneration Gene database (REGene) [17,18]. AmiGo has been widely validated as an optimal gene ontology since 2007 [16]. We used several biological processes, listed in Table 1, that occur during tissue regeneration and have been reported in previous literature [3,19] as keywords to search for human regeneration genes supported by experimental evidence. REGene is a regeneration-targeted informatics resource in humans and animals [17], from which we obtained regeneration genes for bone, dentin, nerve and vessel. For each tissue, we combined the regeneration genes retrieved from the aforementioned two databases. The number of regeneration genes for each tissue is listed in Table 2. Let us denote the regeneration gene sets for bone, dentin, nerve and vessel by $C_{\mathrm{bone}},C_{\mathrm{dentin}},C_{\mathrm{nerve}},C_{\mathrm{vessel}}$. Genes in these sets, together with their Ensembl IDs, are provided in Table S1.

### 2.2. Network Construction

An accurate network can provide abundant information integrated in a rigorous way. In this study, we constructed a protein network in which the candidate genes for co-regeneration in two tissues can be identified. It is known that PPIs play important roles in several intracellular and intercellular biochemical processes. Thus, this information was used to construct the network. Presently, several databases provide PPI information, including the DIP (Database of Interaction Proteins) database [20] and BioGRID [21]. In this study, we used PPI information reported in STRING (Version 9.1) [15]. Unlike PPI information reported in the aforementioned databases, which is validated by traditional experiments, PPI information in STRING is derived from not only experiments but also the following sources: genomic context, (conserved) coexpression and previous knowledge. Accordingly, these interactions indicate physical and functional associations between proteins. Human PPI information was extracted from a file (‘protein.links.v9.1.txt.gz’) that contains PPI information for several organisms, resulting in 2,425,314 human PPIs. In each PPI, there are two proteins, represented by Ensembl IDs, and one score with range between 150 and 999. In detail, the score measures the occurrence of the interaction; specifically, a high score indicates a high probability. In addition, a high interaction score indicates that two proteins have strong associations. For formulation, let us denote the score of an interaction between proteins $p_{1}$ and $p_{2}$ by $S(p_{1},p_{2})$.

The constructed network *G* used all proteins occurring in 2,425,314 human PPIs as nodes, and there were 20,770 nodes in total. An edge in the network represented a human PPI, i.e., two nodes were connected by an edge if and only if their corresponding proteins comprised a human PPI. As mentioned above, the strength of the PPI was represented by its interaction score. Considering the shortest path algorithm would be used in the network-based method, it is not reasonable to assign directly an interaction score to the corresponding edge as a weight because an edge with a low weight indicates strong correlations between its endpoints in a shortest path algorithm based model, which is contrary to the definition of interaction score. Thus, for each edge e, its weight was defined as $w(e)=1000-S(p_{1},p_{2})$, where $p_{1}$ and $p_{2}$ were corresponding proteins for the endpoints of e.

### 2.3. Network-Based Method

A network-based method was built in this section that would be executed on network *G*. This method consisted of three procedures: (I) searching procedure using the shortest path algorithm; (II) testing procedure using a permutation test; (III) and a screening procedure using certain rules. For each pair of gene sets for regeneration of bone, dentin, vessels and nerves, the network-based method yielded several candidate genes that were deemed co-regeneration genes for two tissues. For convenience, we used C and $C^{\prime }$ to denote any two regeneration gene sets for two tissues, i.e., $C,C^{\prime }\in \{C_{\mathrm{bone}},C_{\mathrm{dentin}},C_{\mathrm{nerve}},C_{\mathrm{vessel}}\}$; this notation is utilized in the following description.

#### 2.3.1. Searching Procedure

Several studies have reported that proteins that can interact with each other often share similar functions [22,23,24,25]. This phenomenon implies that a protein that interacts with a protein encoded by a regeneration gene may also be encoded by a regeneration gene. Furthermore, according to the definition of the interaction score, two proteins with a high interaction score are more likely to share similar functions than those with a low interaction score. Thus, a protein that shares a high interaction score with another protein may have a high probability of being encoded by a regeneration gene. In the current study, we sought to identify co-regeneration genes for tissue pairs. If we identify a series of proteins $p_{1},p_{2},\cdots ,p_{s}$ such that consecutive proteins comprise an interaction with a high score and $p_{1},p_{s}$ are encoded by regeneration genes for two different tissues, then the inner proteins $p_{2},\cdots ,p_{s-1}$ may be regeneration genes for both tissues and thus have high probabilities of being co-regeneration genes. According to the definition of network G, the corresponding nodes of $p_{1},p_{2},\cdots ,p_{s}$ may comprise a shortest path. Thus, for any node in C and any node in $C^{\prime }$, we searched for the shortest path connecting them using the Dijkstra’s algorithm [26], a classic shortest path algorithm. The length of shortest path reflects the distance between pairs of genes. Actually, the network-based method using the shortest path algorithm to search novel genes was an extension of the most intuitive method, direct neighbor. If the length of the shortest path between a pair of genes was small, they may be direct neighbors. The basic principle is the modularization of network [27], i.e., genes with similar functions tend to act together, locate in close region of a network, and form a module. The shortest path can be an approximate of signaling pathway. After collecting all shortest paths, inner nodes were extracted, and their corresponding genes were accessed. The obtained genes were called shortest path genes. In addition, to ascertain the importance of each shortest path gene, a measurement of betweenness [28] was calculated for it, defined as the number of shortest paths containing it. In fact, the betweenness indicated the direct and indirect influences of genes in a distant network [29], suggesting the direct and indirect association with genes in C and $C^{\prime }$.

#### 2.3.2. Testing Procedure

Based on regeneration genes for two tissues, possible co-regeneration genes can be detected using the shortest path algorithm and designated shortest path genes. However, this set of genes inevitably includes false discoveries. To control such false discoveries as much as possible, a permutation test was performed. For one regeneration gene set C, 1000 gene sets were randomly constructed (denoted by $C_{1},C_{2},\ldots ,C_{n}$); each set had the same size as C. Furthermore, to give a strict permutation test, degree distribution of genes (nodes) in each set was same as that for C, i.e., the numbers of nodes with same degrees in C and $C_{i}\;(i=1,2,\cdots 1000)$ were identical. Following the same rules, for the regeneration gene set $C^{\prime }$, we randomly constructed 1000 gene sets (denoted by $C_{1}\prime ,C_{2}\prime ,\cdots ,C_{1000}\prime$). For $C_{i}$ and $C_{i}\prime$ (i=1,2,⋯,1000), the shortest paths in G connecting any gene in $C_{i}$ and any gene in $C_{i}\prime$ were determined. The derived shortest paths were used to calculate betweenness for each shortest path gene. Accordingly, 1000 betweenness values on randomly constructed set pairs were obtained for each shortest path gene. By comparing betweenness on C and $C^{\prime }$, a permutation false discovery rate (FDR) measure was calculated as:

(1) $$FDR(g)=\frac{\Delta }{1000}$$

where Δ is the number of randomly produced set pairs for which betweenness was greater than that of C and $C^{\prime }$. It is clear that the range of permutation FDR was between 0 and 1. A high permutation FDR means the corresponding gene can be produced by numerous gene set pairs, and it is not specific for the gene sets C and $C^{\prime }$. Thus, we should select shortest path genes with low permutation FDRs. Because 0.05 is widely accepted as the significance cutoff for statistical tests, we set 0.05 as the threshold for permutation FDR, i.e., shortest path genes with permutation FDRs less than 0.05 were selected for further evaluation. For convenience, the selected shortest path genes were referred to as candidate genes.

#### 2.3.3. Screening Procedure

The testing procedure thus produced a set of candidate genes. Most of these genes had weak or strong associations with the regeneration systems of two tissues. To select the most essential genes among them, two screening rules were constructed.

*Betweenness ratio rule.* Each candidate gene was assigned a betweenness value as mentioned in Section 2.3.1. This betweenness value intuitively indicates the association between the candidate genes and two regeneration systems. A candidate gene with high betweenness is more likely to be a co-regeneration gene for two tissues. However, because this study considered four tissues, it would not be reasonable only to consider the absolute magnitude of betweenness. Different pairs of tissues have different backgrounds, such as regeneration gene sets of different sizes. Inspired by Freeman’s betweenness measure [30,31], defined as

(2) $$C_{B}(v)=\sum_{s\neq v\neq t}\frac{\delta_{st}(v)}{\delta_{st}}$$

where s and t represent any two nodes in a network, $\delta_{st}$ represents the number of shortest paths connecting s and t, and $\delta_{st}(v)$ represents the number of shortest paths connecting s and t and containing v, we considered the relative value of betweenness, namely, betweenness ratio, for each candidate gene. This measure can be calculated as:

(3) $$BR(g)=\frac{bet(g)}{\left|C\right|\cdot \left|C^{\prime }\right|}$$

where bet(g) represents the betweenness of g. The denominator of Equation (3) represents the number of possible shortest paths connecting genes in C and $C^{\prime }$. Thus, the betweenness ratio indicates the proportion of paths containing the candidate gene among all possible paths. Clearly, a high betweenness ratio means that the candidate gene is more likely to be a co-regeneration gene. We applied a threshold of 0.01 for betweenness ratio, i.e., candidate genes with betweenness ratios greater than 0.01 were selected.

*Interaction score rule.* This rule utilized the fact that proteins that interact with each other can share similar functions [22,23,24,25]. If a candidate gene has strong associations with regeneration genes for two tissues, it is deemed to be a co-regeneration gene with a high probability. Thus, for each candidate gene *g*, we computed its Min-Max interaction score as follows:

(4) $$\mathrm{Min}-\mathrm{Max}(g)=\mathrm{Min}\{\mathrm{Max}\{S(g,g_{a}):g_{a}\in C\},\mathrm{Max}\{S(g,g_{b}):g_{b}\in C^{\prime }\}\}$$

Similarly, we selected candidate genes with high Min–Max interaction scores. Because 400 was set as the cutoff for medium confidence, it was set as the threshold of for Min–Max interaction scores, i.e., candidate genes with Min–Max interaction scores greater than or equal to 400 were selected.

According to the screening rules mentioned above, if a candidate gene was assigned a betweenness ratio larger than 0.01 and a Min–Max interaction score no less than 400, it would be selected as a novel co-regeneration gene for two tissues.

## 3. Results

**Figure 1:**
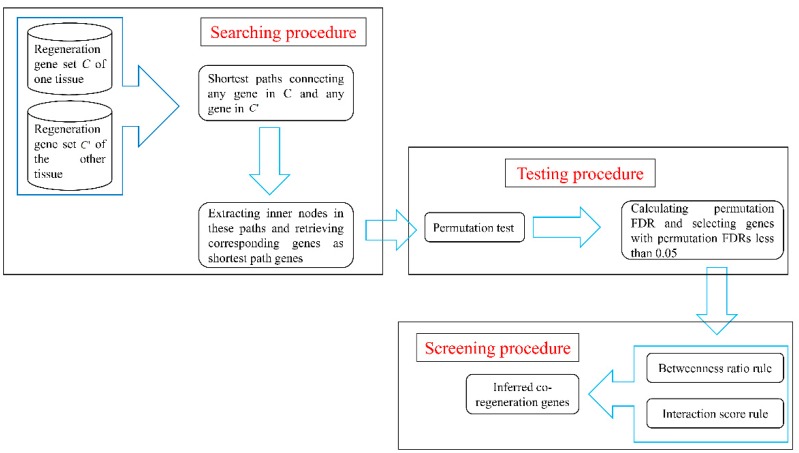
A flow chart to illustrate the network-based method. FDR: false discovery rate.

The network-based method described in Section 2.3 was applied to identify co-regeneration genes for every pair of the four tissues. The flow chart in Figure 1 illustrates this network-based method. This section gives a detailed description of results obtained in each of the three procedures of this method.

*Shortest path genes obtained in the searching procedure.* As mentioned in Section 2.3.1, the shortest path in network *G* connecting any regeneration gene for one tissue and any regeneration gene of another tissue was calculated. Corresponding genes of inner nodes in these paths were extracted as shortest path genes. Shortest path genes for each pair of tissues as well as their betweenness values are provided in Table S2. The number of shortest path genes for each pair of tissues is listed in column 2 of Table 3, from which we can see that several shortest path genes were obtained. This list is likely to include many false discoveries; thus, further procedures to select relevant co-regeneration genes are necessary.

**Figure 2:**
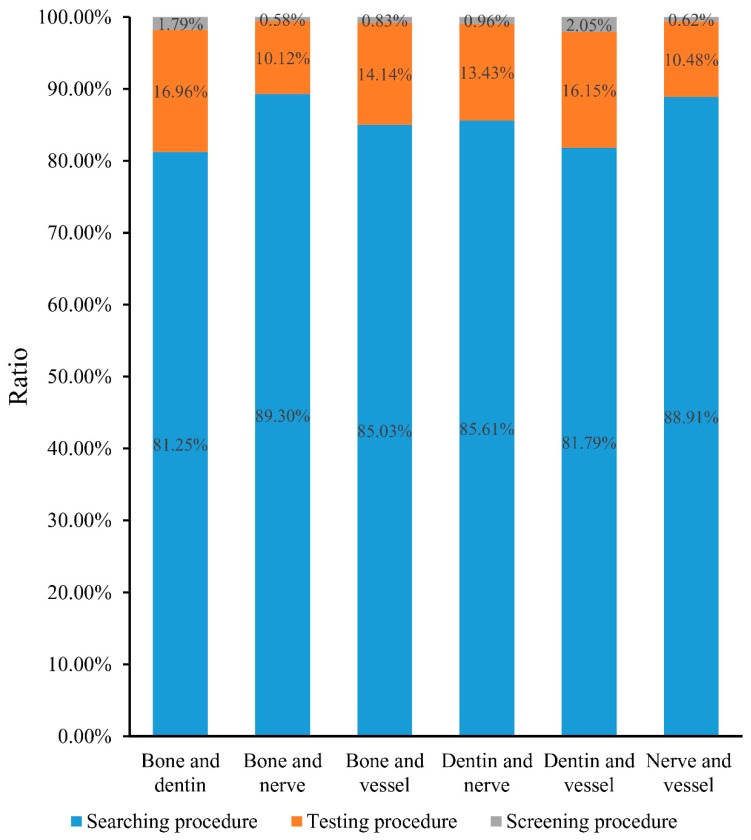
A bar chart to illustrate the ratio of shortest path genes obtained in one procedure and screened out in the following procedure. In detail, blue represents the ratio of shortest path genes that were obtained in the searching procedure and screened out in testing procedure; orange represents the ratio of shortest path genes that were obtained in testing procedure and screened out in screening procedure; and gray represents the ratio of shortest path genes that were obtained in the screening procedure.

*Candidate genes obtained in the testing procedure.* For the obtained shortest path genes, a permutation test was performed to examine whether each of them was a false discovery. To evaluate each shortest path gene quantitatively in this regard, a permutation FDR (cf. Equation (1)) was calculated; these values are also provided in Table S2. Then, shortest path genes with permutation FDRs less than 0.05 were selected as candidate genes. Obtained candidate genes for each pair of tissues are provided in Table S2, and numbers of these candidate genes is listed in column 3 of Table 3. Several shortest path genes were screened out, and the number of possible genes sharply decreased. The bar chart in Figure 2 intuitively illustrates the ratio of genes obtained in the searching procedure and screened out in the following procedure. More than 80% of shortest path genes were filtered by the testing procedure for each pair of tissues, which indicates that the testing procedure helped us discard a large number of false discoveries.

*Inferred genes obtained in the screening procedure.* Two rules were employed in the screening procedure. After applying them to the candidate genes obtained in testing procedure, a subset of candidate genes remained; these genes are provided in Table 4. The number of final inferred genes for each pair of tissues is listed in column 4 of Table 3; the number of inferred genes was no more than 8 in all cases, representing an appropriate scale of data that a biologist can analyze in depth. It can also be observed from Figure 2 that more than 10% of shortest path genes were discarded in this procedure, and less than 3% of shortest path genes remained. These results indicated that the screening procedure was highly effective for selecting essential genes.

**Figure 3:**
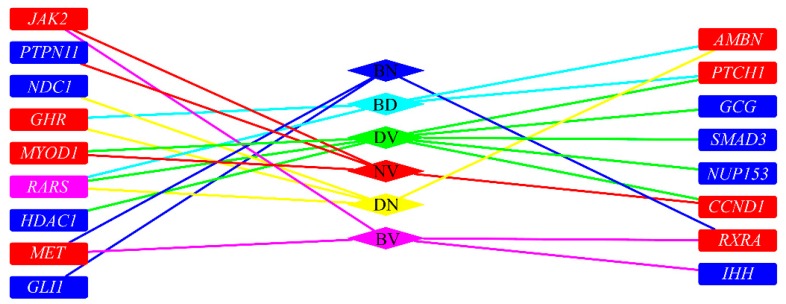
The relationship of inferred co-regeneration genes and pairs of tissues. BD represents bone and dentin, BV represents bone and vessels, BN represents bone and nerves, DN represents dentin and nerves, DV represents dentin and vessels, NV represents nerves and vessel. Blue/red/pink rectangles (representing inferred genes) represent the genes were inferred to one/two/three pairs of tissues. If a rectangle is adjacent to an edge whose color is the same as a diamond (representing a pair of tissues), the inferred gene is an inferred co-regeneration gene for these two tissues.

**Figure 4:**
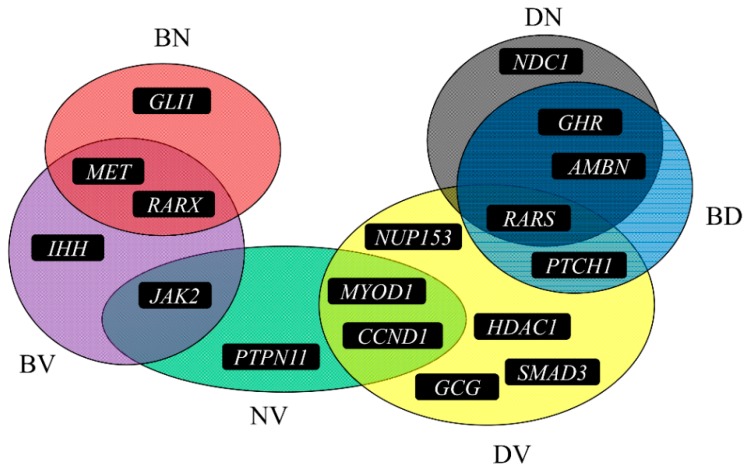
A Venn diagram to illustrate the six sets of inferred genes for six pairs of tissues. BD represents bone and dentin, BV represents bone and vessels, BN represents bone and nerves, DN represents dentin and nerves, DV represents dentin and vessels, NV represents nerves and vessel.

To illustrate inferred co-regeneration genes of each pair of tissues, a graph is plotted in Figure 3. It can be observed that seventeen genes were inferred to be co-regeneration genes for some pair of tissues. In detail, the gene, *RARS*, were deemed to be a co-regeneration gene for three pairs of tissues, eight genes (*MET*, *RARX*, *JAK2*, *MYOD1*, *CCND1*, *PTCH1*, *GHR*, *AMBN*) were deemed to be co-regeneration genes for two pairs of tissues, and rest eight genes (*GLI1*, *IHH*, *PTPN11*, *NUP153*, *HDAC1*, *SMAD3*, *GCG*, *NDC1*) were deemed to be co-regeneration genes for one pair of tissues. A clearer Venn diagram is illustrated in Figure 4. For four inferred genes of bone and dentin (*GHR*, *AMBN*, *RARS*, *PTCH1*), *GHR*, *RARS* and *AMBN* were deemed to contribute to co-regeneration of dentin and nerve, inducing they can also contribute to regeneration of nerve; *PTCH1* was considered to contribute to co-regeneration of dentin and vessel, suggesting it can contribute to regeneration of vessel. With similar arguments, for four inferred genes of nerve and vessel (*JAK2*, *PTPN11*, *MYOD1*, *CCND1*), *JACK2* was inferred to contribute to regeneration of bone, while *MYOD1* and *CCND1* were deemed to contribute to regeneration of dentin. In Section 4, the detailed analyses of these seventeen inferred genes are given.

## 4. Discussion

Based on a network-based method, seventeen genes, listed in Table 4, were identified that may contribute to co-regeneration of some pair of hard tissues (like bone and dentin) and environmentally associated soft tissues (nerve, vessels). According to recent publications, all such genes have been directly confirmed or strongly implicated as contributing to the regenerative processes in human bodies, validating the reliability of the results yielded by the network-based method. Detailed analyses of seventeen genes can be seen below.

### 4.1. Co-Regeneration Genes for Bone and Dentine

Our computational method identified four genes (*RARS*, *GHR*, *AMBN*, *PTCH1*) that contribute to co-regeneration of bone and dentin. Given the similar structure and origins of bone and dentin, all genes may contribute to regenerative processes in both bone and dentin and affect treatment of periodontal diseases in which both simultaneous bone regeneration and dental repair are necessary.

*RARS* (ENSP00000231572) may contribute to the co-regeneration of such two tissues. Generally, such gene encodes specific transfer RNA (tRNA) synthetases that participate in the aminoacylation of tRNA [32]. According to recent publications, a recent research on bone anabolism [33] confirmed that tRNA aminoacylation which *RARS* may definitely interact with contribute to the regeneration processes of osseous tissue. Apart from validated relationship with bone regeneration, such gene has also been confirmed to be an irreplaceable regulatory cellular component during the regenerative processes of dentin. In 2012, a specific study on Charcot-Marie-Tooth disease (CMT) [34] confirmed that abnormal biological functions of *RARS* may contribute to the initiation and progression of CMT, a unique disease involving grinding of teeth, validating the unique role of this gene during tooth regeneration [35]. Therefore, considering the complicated biological functions of such gene, it is quite reasonable to regard *RARS* as a potential co-regeneration gene for both bone and dentin. The following gene *GHR* (ENSP00000230882) has also been predicted to contribute to the co-regeneration of dentin and bone. According to recent publications, *GHR* encodes a functional transmembrane receptor for growth hormone as a member of the type I cytokine receptor family [36]. As for its specific functions during the co-regeneration of bone and dentin, as one of the osteotropic growth factor, a specific study on canine model [37] validates the potential regulatory role of *GHR* during the regeneration of bone. Furthermore, early in 2000, based on the Lewis rat, *GHR* has been confirmed to contribute to the formation of dentine companying a glucocorticosteroid treatment, validating the co-regeneration role of such gene in both two tissues [38]. The following inferred gene *AMBN* (ENSP00000313809) encodes a functional extracellular matrix protein in the dentin microenvironment, which has been confirmed to participate in the regeneration of the tooth root [39,40]. As for its functional roles during bone regeneration, it has been confirmed that such gene directly regulates the remodeling and repair of osseous tissues [41,42], verifying its specific co-regenerative biological functions. The last gene turns out to be *PTCH1* (ENSP00000332353). According to recent publications, such gene has been reported to encode a functional receptor for sonic hedgehog, a secreted development and tumorigenesis associated molecule [43,44]. As a functional component of hedgehog signaling pathway [45], *PTCH1* has been widely reported to participate in the regeneration of bone in human beings and various animal models [46,47]. Though only a few researches concentrated on the biological functions of this gene during dentin regeneration, it has been confirmed that just like *AMBN* we have analyzed above, *PTCH1* may also be critical for tooth root formation and further participate in the regeneration of dentin tissues [48].

### 4.2. Co-Regeneration Genes for Bone and Nerve

Apart from four genes that have been inferred and literature validated to be co-regeneration genes for bone and dentin, we also identified three functional co-regeneration genes (*MET*, *RXRA*, *GLI1*) for bone and nerve. *MET* (ENSP00000317272) as a member of the receptor tyrosine kinase family and a functional proto-oncogene, has been confirmed to contribute to various development and tumorigenesis associated biological processes [49]. As for its potential biological functions for the regeneration of bone and nerve, according to recent publications, *MET* contributes to the differentiation of mesenchymal stem cells (MSCs) [50]. Considering that both neurons and osteocytes can be derived from MSCs [51], it is quite reasonable to speculate *MET* as an irreplaceable regulator during bone and nerve co-regeneration [51,52]. As for a detailed explanation, in 2015, a specific study [53] on MSCs confirmed that this gene may directly participate in the regeneration of bone according to a clinical application, indicating that *MET* may be related to bone regeneration. Apart from that, *MET* has also been widely reported to participate in the regeneration and repair of nerve after functional injury. In 2013, a novel study [54] on nerve regeneration confirmed the irreplaceable regulatory role of such gene in primitive neural stem cells, implying its specific regenerative contributions [54]. *RXRA* (ENSP00000419692) as another inferred co-regenerative gene has been widely reported to encode a member of functional retinoic acid receptors [55]. As for its co-regenerative functions, in 2014, it has been reported that the methylation status of *RXRA*’s promotor is associated with the bone mineral content and osteogenesis, indicating that such gene may also contribute to the regeneration of bone [56]. Furthermore, in 2015, Natrajan et al. published a specific study [57] demonstrating that *RXRA* may reverse the age-related deficiencies in myelin debris phagocytosis and remyelination which are both specific functional component for nerve regeneration. *GLI1* (ENSP00000228682) encoding a member of the Kruppel family of zinc finger proteins, has also been inferred as a bone-nerve co-regeneration gene. According to recent publications, *GLI1* has been confirmed to regulate the regenerative capacity of osseous tissues by participating in hedgehog signaling pathway as we have analyzed above [58,59]. As for its potential role in nerve system, considering that hedgehog signaling pathway has also been confirmed to participate in peripheral nerve regeneration [60], as one unique component of such pathway, *GLI1* has also been reported to be downregulated during such regenerative processes [61].

### 4.3. Co-Regeneration Genes for Bone and Vessel

Four functional genes (*IHH*, *MET*, *JAK2*, *RXRA*) have been inferred to contribute to the co-regeneration of bone and vessel. *IHH* (ENSP00000295731) as a hedgehog pathway associated gene has been reported to be expressed and functioning in liver, kidney and skeletal system by regulating SOX9 [62,63]. As for its specific role for bone and vessel co-regeneration, expressed by MSCs, *IHH* has been confirmed to be overexpressed during the repair and regeneration of bone, indicating its specific role of bone regeneration [64]. At the same time, during the regeneration of bone, together with *GLI1* as we have analyzed above, our predicted gene *IHH* has also been confirmed to contribute to vascularization mediating by hedgehog signaling pathway [65]. As we have mentioned above, *MET* (ENSP00000317272) has been confirmed to participate in the co-regeneration of bone and nerve [51,52]. According to recent publications, such gene may further participate in the co-regeneration of bone and vessels. Participating in HGF/c-Met signaling pathway, this gene contributes to the differentiation of human vascular smooth muscle cells as well as osteoblasts, indicating its specific role during bone and vessel co-regeneration and the potential relationship between the generation of such two tissue subtypes [66]. *JAK2* (ENSP00000371067), as a specific protein tyrosine kinase encoding gene, has been generally reported to involve in various cytokine receptor associated biological processes [67,68]. In 2011, a review [69] on the genetic contribution on the regeneration of vascular walls confirmed that JAK/STAT pathway which *JAK2* directly participate in the vessel regeneration processes around the bone tissue, confirming our inference. As for its specific functions during bone regeneration, 4-methoxydalbergione, as a functional chemical, has been reported to suppress the growth and regeneration of human osteoblast proliferation and differentiation by down-regulating JAK2/STAT3 pathway, validating the potential role of *JAK2* during the regeneration of bone [70]. Therefore, *JAK2* may definitely contribute to the co-regeneration of bone and vessel. *RXRA* (ENSP00000419692) as we have analyzed above has been confirmed to contribute to the co-regeneration of bone and nerve. Furthermore, such gene has also been predicted to contribute to the co-regeneration of bone and vessel. In 2004 a specific report [71] confirmed that the coupled development of osseous tissues and vascular tissues in the medaka fish are regulated by *RXRA*. Therefore, combined with related studies that demonstrating the functions of *RXRA* [72,73], it is quite reasonable to speculate that *RXRA* may act similarly in human beings.

### 4.4. Co-Regeneration Genes for Dentin and Nerve

Similar with bone, dentin, as another subtype of “hard” tissue, has also been identified to have co-regenerative regulatory genes shared with surrounding tissues like nerves and vessels. Four genes (*NCD1*, *AMBN*, *RARS*, *GHR*) were inferred to be co-regeneration genes for dentin and nerve. *NDC1* (ENSP00000360483), as the component of the nuclear pore complex (NPC), has been widely reported to contribute to the de novo assembly and insertion of NPC in the nuclear envelope [74]. Early in 1985, a specific study [75] on the human dental pulp confirmed that *NDC1* contributes to the generation of nerve fibres in tooth during the dentin development, confirming the co-regenerative functions of such gene. The following gene *AMBN* (ENSP00000313809) has already been analyzed above as a co-regenerative gene for bone and dentin. During *AMBN*-mediated dentin development, such gene has also been identified to participate in the development of surrounding nerves [76]. In 2008, a specific study [76] on dental tissues confirmed that as the downstream of a specific neuron development associated factor neurotrophin-4, *AMBN* may also participate in nerve development associated biological processes. *RARS* (ENSP00000231572), as a functional co-regeneration associated gene has already been analyzed in Section 4.1, can be confirmed its potential role during the co-regeneration of bone and dentin. Here, it is further inferred as a co-regenerative gene for dentin and nerve. As we have analyzed above, such gene has been confirmed to contribute to the regeneration of dentin. In 2015, a specific study on dopaminergic neuronal differentiation confirmed that *RARS* can participate in the regulation of trophoblast stem cells [77]. Considering that trophoblast stem cells have been validated to contribute to dentin generation [78], it is quite reasonable to summarize such gene as a co-regenerative gene for both dentin and nerve. Just like *RARS*, another inferred gene *GHR* (ENSP00000230882), which has been proved to contribute to the co-regeneration of bone and dentin, has further been inferred to participate in the regeneration of nerve. During the development of dentin, *GHR* has been confirmed to participate in the formation of surrounding nerve tissues [79,80].

### 4.5. Co-Regeneration Genes for Dentin and Vessel

For dentin and vessel, we screened out eight inferred genes (*RARS*, *HDAC1*, *NUP153*, *GCG*, *CCND1*, *SMAD3*, *PTCH1*, *MYOD1*) that definitely contribute to such biological processes validated by recent publications. The detailed analysis can be seen below. *RARS* (ENSP00000231572) and *PTCH1* (ENSP00000332353) are two functional genes that contribute to the co-regenerative processes, which have been analyzed above. *RARS* has been confirmed to participate in the co-regeneration of bone and dentin as well as dentin and nerve. As for its specific function during the co-regeneration of dentin and vessel, in 2001, a unique study [81] on the ligand of *RARS* encoding protein has been confirmed to contribute to the generation of orordental tissues and surrounding vessel, validating such co-regenerative functions. Similarly, *PTCH1*, as an inferred co-regeneration gene of bone and dentin, has also been validated to contribute to the regeneration of dentin and vessels based on recent publications. Such gene encodes a receptor for sonic hedgehog as we have analyzed above participating in the hedgehog signaling pathway [43]. Considering that hedgehog signaling pathway has been confirmed to contribute to the co-regeneration of dentin and vessel [43], it is quite reasonable to regard *PTCH1* as a co-regenerative gene for dentin and vessels. Apart from such two analyzed genes, we also obtained six additional genes for the co-regeneration of dentin and vessel. *HDAC1* (ENSP00000362649) encodes a functional component of the histone deacetylase complex, acting as a key regulator for the gene expression regulation. Interacting with *HDAC6*, this gene has also been confirmed to participate in the pathogenesis of CMT, indicating its potential contribution to dentin regeneration [82]. During its regulation on dentin, various publications have also confirmed its specific role for angiogenesis, indicating its co-regenerative role [83]. *NUP153* (ENSP00000262077), as another nuclear pore complex associated gene, has been inferred to contribute to the co-regeneration of dentin and vessel. According to recent publications, *NUP153* has only been reported to participate in the regulation on the generation of novel vessel [84]. As a functional component of the nuclear pore complexes, considering that nuclear pore complexes have been reported to contribute to the generation of dentin [75], it is reasonable to summarize that NUP153 is definitely a co-regenerative gene for both dentin and vessel [84]. *GCG* (ENSP00000387662) has been widely reported to contribute to glucose metabolism and homeostasis [85,86]. According to recent publications, *GCG* has been confirmed to enhance the dentin bio modification potentials, implying its potential functions during dentin regeneration [87]. Apart from such dentin regulating functions, *GCG* has further been validated on mouse model to contribute to angiogenesis, validating its co-regenerative roles [88]. *CCND1* (ENSP00000227507) has been widely reported to act as a regulator of progression through G1 phase during the cell cycle [89,90]. Regulating the β-catenin signaling pathway in the dental periodontal ligament cells, *CCND1* has been confirmed to contribute to the generation of dentin tissues [91]. As for its co-regenerative contribution to vessel, during the regulation of periodontal ligament cells, another publications confirmed that *CCND1* also participates in the blood wall remodeling processes [92]. The next gene *SMAD3* (ENSP00000332973) has been widely reported to act as an intracellular signal transducer and transcriptional modulator activated by transforming growth factor beta (TGF-β) [93,94]. Contributing to BMPs regulating biological pathways [95], such gene has been reported to participate in the regeneration of dentin pulp cells and the re-modulation of neighborhood vascular wall cells, confirming it being a co-regeneration gene [96,97]. *MYOD1* (ENSP00000250003) has been confirmed to participate in the PPARβ-associated pathways and further contributes to angiogenesis in various tissues [98]. As for its contribution to dentin regeneration, a recent study [99] on familial hypodontia confirmed that the abnormal function of this gene may induce a pathological regenerative pattern of dentin tissues.

### 4.6. Co-Regeneration Genes for Nerve and Vessel

Four genes (*MOYOD1*, *PTPN11*, *CCND1*, *JAK2*) were inferred by the network-based method as the co-regeneration genes of nerve and vessel. As we have analyzed above, *MYOD1* (ENSP00000250003) is definitely involve in the angiogenesis processes. Moreover, MYOD1 has been reported to contribute to regeneration of the nervous system by regulating parallel differentiation of embryonic stem cells, supporting a dual regulatory function that may involve regenerative processes of both nerves and vessels [100]. *PTPN11* (ENSP00000340944), encoding a functional protein tyrosine phosphatase with two SH2 domains, has been confirmed to be widely expressed in multiple tissue subtypes [101,102]. Also known as SHP2, such gene has been confirmed to contribute to the regeneration of nerve tissues by regulating Shp2/PI3K/Akt Pathway [103,104]. As for its contribution to angiogenesis, various publications confirmed that *PTPN11* contributes to the generation of vessel cells, confirming its role for regeneration of vessel [105]. As we have analyzed above, *CCND1* (ENSP00000227507) contributes to the co-regeneration of dentin and vessels. Further researches on such field revealed that during the co-regenerative processes, *CCND1* also mediated the generation of nervous tissues [106,107]. The last gene *JAK2* (ENSP00000371067) has already been confirmed to act as a co-regenerative factor contributing to the co-regeneration of bone and vessel. During such co-regenerative process, it has also been confirmed to participate in the generation of the nerve system. In 2015, a specific study [108] on neuron stem cells confirmed that the differentiation of neurons may be regulated by such gene.

As discussed above, all inferred genes have been confirmed to contribute to the co-regeneration of at least two tissues. These current findings indicate that the network-based method can identify novel co-regeneration genes of multiple tissues, providing novel materials for research on regeneration systems in bone, dentin, nerves and vessels.

### 4.7. Other Applications of the Network-Based Method

The network-based method built in this study can be applied in many similar questions to integrate multiple data and infer shared drivers. For example, a similar method has been used to identify the candidate drivers of lung adenocarcinoma by integrating dysfunctions of mutation, methylation, microRNA, and mRNA [5]. Another application was to find the hidden genes that facilitate the breast cancer metastasis to bone [13]. The cancer genes in breast and the cancer genes in bone were connected by shortest paths on the network. Overall, this method can be used for data integration on network and biological mechanism investigation for complex diseases or biological processes.

## 5. Conclusions

In the current study, a network-based method was employed to identify novel co-regeneration genes for bone, dentin, nerves and vessels. Based on regeneration genes for paired tissues, the shortest path algorithm was applied in a constructed network to detect possible co-regeneration genes. Testing and screening procedures were further employed to select essential candidate genes. Seventeen inferred genes were analyzed in depth, indicating that they may be novel co-regeneration genes. The new genes proposed in this study may provide new insights into the study of regeneration systems for these four tissues.

## Figures and Tables

**Table 1:** Key words for retrieving regeneration genes from AmiGo.

Category	Biological Process	GO Term ID
Bone regeneration	Bone regeneration	GO:1990523
	Bone mineralization	GO:0030282
	Bone marrow development	GO:0048539
	Bone remodeling	GO:0046849
	Bone morphogenesis	GO:0060349
Dentin regeneration	Odontoblast differentiation	GO:0071895
	Dentinogenesis	GO:0097187
	Structural constituent of tooth enamel	GO:0030345
	Regulation of tooth mineralization	GO:0070170
	Dentin secretion	GO:0070468
	Dentin mineralization	GO:0097188
	Tooth mineralization	GO:0034505
Vessel regeneration	Vascular wound healing	GO:0061042
	Blood vessel morphogenesis	GO:0048514
	Blood vessel remodeling	GO:0001974
	Blood vessel development	GO:0001568

**Table 2:** Number of regeneration genes for bone, dentin, vessels and nerves.

Category	Number of Regeneration Genes
Bone regeneration	144
Dentin regeneration	31
Vessel regeneration	336
Nerve regeneration	284

**Table 3:** Number of genes obtained in each procedure of the network-based method.

Paired Tissues	Searching Procedure (Inner Nodes of Shortest Paths)	Testing Procedure (Permutation FDR < 0.05)	Screening Procedure (Betweenness Ratio > 0.01 and Min–Max Interaction Score ≥ 400)
Bone and dentin	244	42	4
Bone and nerve	514	55	3
Bone and vessel	481	72	4
Dentin and nerve	417	60	4
Dentin and vessel	390	71	8
Nerve and vessel	649	72	4

**Table 4:** Inferred co-regeneration genes for paired tissues.

Pair of Tissues	Ensembl ID	Gene Symbol	Full Gene Name	Betweenness	Permutation FDR	Betweenness Ratio	Min-Max Interaction Score	Major Biological Functions
Bone and dentine	ENSP00000231572	*RARS*	Arginyl-TRNA Synthetase	122	<0.001	0.037	865	Catalyzes the attachment of specific amino acids to cognate tRNAs during protein synthesis. Modulates the secretion of AIMP1.
	ENSP00000230882	*GHR*	Growth Hormone Receptor	122	0.011	0.037	675	Receptor for pituitary gland growth hormone involved in regulating postnatal body growth, contributing to JAK2/STAT5 pathway.
	ENSP00000313809	*AMBN*	Ameloblastin	122	0.012	0.037	583	Involves in the mineralization and structural organization of enamel.
	ENSP00000332353	*PTCH1*	Patched 1	155	0.033	0.047	878	Acts as a receptor for multiple hedgehog signaling pathways. Associates with the smoothened protein (SMO) to transduce the hedgehog proteins signal.
Bone and nerves	ENSP00000317272	*MET*	MET Proto-Oncogene, Receptor Tyrosine Kinase/hepatocyte growth factor receptor	802	0.019	0.023	989	Receptor tyrosine kinase that transduces signals from the extracellular matrix into the cytoplasm by binding to hepatocyte growth factor, regulating proliferation, scattering, morphogenesis and survival.
	ENSP00000419692	*RXRA*	Retinoid X Receptor Alpha	459	0.026	0.013	964	Receptor for retinoic acid. Binds as heterodimers to its target response elements in response to their ligands, all-trans or 9-cis retinoic acid, and regulates gene expression in various biological processes.
	ENSP00000228682	*GLI1*	GLI Family Zinc Finger 1	407	0.044	0.012	978	Regulates the transcription of specific genes during normal development. Plays a role in development of multiple tissues. Mediates SHH signaling.
Bone and vessels	ENSP00000295731	*IHH*	Indian Hedgehog	486	0.014	0.015	912	Intercellular signal essential for a variety of patterning events during development. Binds to the patched (PTC) receptor, which functions in association with smoothened (SMO), to activate the transcription of target genes.
	ENSP00000317272	*MET*	MET Proto-Oncogene, Receptor Tyrosine Kinase/hepatocyte growth factor receptor	634	0.018	0.02	984	Receptor tyrosine kinase that transduces signals from the extracellular matrix into the cytoplasm by binding to hepatocyte growth factor, regulating proliferation, scattering, morphogenesis and survival.
	ENSP00000371067	*JAK2*	Janus Kinase 2	853	0.021	0.027	994	Non-receptor tyrosine kinase involving in various processes such as cell growth, development, differentiation or histone modifications. Mediates essential signaling events in both innate and adaptive immunity.
	ENSP00000419692	*RXRA*	Retinoid X Receptor Alpha	353	0.026	0.011	964	Receptor for retinoic acid. Binds as heterodimers to its target response elements in response to their ligands, all-trans or 9-cis retinoic acid, and regulates gene expression in various biological processes.
Dentin and nerves	ENSP00000360483	*NDC1*	NDC1 Transmembrane Nucleoporin	280	0.004	0.037	466	Component of the nuclear pore complex (NPC), contributing to de novo assembly and insertion of NPC in the nuclear envelope. Required for NPC and nuclear envelope assembly.
	ENSP00000313809	*AMBN*	Ameloblastin	280	0.012	0.037	819	Involves in the mineralization and structural organization of enamel.
	ENSP00000231572	*RARS*	Arginyl-TRNA Synthetase	281	0.015	0.037	865	Catalyzes the attachment of specific amino acids to cognate tRNAs during protein synthesis. Modulates the secretion of AIMP1.
	ENSP00000230882	*GHR*	Growth Hormone Receptor	281	0.04	0.037	675	Receptor for pituitary gland growth hormone involved in regulating postnatal body growth, contributing to J AK2/STAT5 pathway.
Dentin and vessels	ENSP00000231572	*RARS*	Arginyl-TRNA Synthetase	258	0.003	0.037	865	Catalyzes the attachment of specific amino acids to cognate tRNAs during protein synthesis. Modulates the secretion of AIMP1.
	ENSP00000362649	*HDAC1*	Histone Deacetylase 1	112	0.01	0.016	993	Responsible for the deacetylation of lysine residues on the N-terminal part of the core histones. Gives a tag for epigenetic repression and plays an important role in transcriptional regulation, cell cycle progression and developmental events.
	ENSP00000262077	*NUP153*	Nucleoporin 153	251	0.024	0.036	456	Component of the nuclear pore complex (NPC), a complex required for the trafficking across the nuclear envelope. Functions as a scaffolding element in the nuclear phase of the NPC essential for normal nucleocytoplasmic transport of proteins and mRNAs.
	ENSP00000387662	*GCG*	Glucagon	76	0.026	0.011	896	Plays a key role in glucose metabolism and homeostasis. Regulates blood glucose. Raises plasma glucose levels in response to insulin-induced hypoglycemia. Plays an important role in initiating and maintaining hyperglycemic conditions in diabetes.
	ENSP00000227507	*CCND1*	Cyclin D1	263	0.03	0.038	946	Regulatory component of the cyclin D1-CDK4 (DC) complex that phosphorylates and inhibits members of the retinoblastoma (RB) protein family including RB1 and regulates the cell-cycle during G(1)/S transition.
	ENSP00000332973	*SMAD3*	SMAD Family Member 3	490	0.038	0.07	875	Receptor-regulated SMAD (R-SMAD) that is an intracellular signal transducer and transcriptional modulator activated by TGF-β (transforming growth factor) and activin type 1 receptor kinases. Binds the TRE element in the promoter region of many genes that are regulated by TGF-β and, on formation of the SMAD3/SMAD4 complex, activates transcription.
	ENSP00000332353	*PTCH1*	Patched 1	131	0.044	0.019	878	Acts as a receptor for multiple hedgehog signaling pathways. Associates with the smoothened protein (SMO) to transduce the hedgehog proteins signal.
	ENSP00000250003	*MYOD1*	Myogenic Differentiation 1	208	0.048	0.0299	848	Promotes transcription of muscle-specific target genes in muscle differentiation. Together with MYF5 and MYOG, co-occupies muscle-specific gene promoter core region during myogenesis. Induces fibroblasts to differentiate into myoblasts. Interacts with and is inhibited by the twist protein.
Nerves and vessels	ENSP00000250003	*MYOD1*	Myogenic Differentiation 1	2465	0.005	0.034	999	Promotes transcription of muscle-specific target genes in muscle differentiation. Together with MYF5 and MYOG, co-occupies muscle-specific gene promoter core region during myogenesis. Induces fibroblasts to differentiate into myoblasts. Interacts with and is inhibited by the twist protein.
	ENSP00000340944	*PTPN11*	Protein Tyrosine Phosphatase, Non-Receptor Type 11	1350	0.01	0.019	999	Involved in intracellular signal transduction in response to PDGF, EGF, insulin.
	ENSP00000227507	*CCND1*	Cyclin D1	3568	0.022	0.049	991	Regulatory component of the cyclin D1-CDK4 (DC) complex that phosphorylates and inhibits members of the retinoblastoma (RB) protein family including RB1 and regulates the cell-cycle during G(1)/S transition.
	ENSP00000371067	*JAK2*	Janus Kinase 2	2332	0.031	0.032	999	Non-receptor tyrosine kinase involving in various processes such as cell growth, development, differentiation or histone modifications. Mediates essential signaling events in both innate and adaptive immunity.

## Supplementary Materials

The following are available online at www.mdpi.com/2073-4425/8/10/252. Table S1: Genes for bone regeneration, dentin regeneration, vessel regeneration and nerve regeneration, Table S2: Genes obtained by the network-based method and their measurements.
