# Supplementary material for: Network-Based Method for Identifying Co-Regeneration Genes in Bone, Dentin, Nerve and Vessel Tissues
Source: Genes (Basel). 2017 Oct 2;8(10):252. doi: 10.3390/genes8100252 (PMC5664102; doi:10.3390/genes8100252)
Supplement: Supplementary file 1 [file genes-08-00252-s001.zip › Table_S1.docx]

**Table S1.** Genes for bone regeneration, dentin regeneration, vessel regeneration and nerve regeneration

1. Genes for bone regeneration

| **Gene symbol** | **Ensembl ID** |
| --- | --- |
| PTGS2 | ENSP00000356438 |
| CDX1 | ENSP00000231656 |
| CHSY1 | ENSP00000254190 |
| GLG1 | ENSP00000205061 |
| PKDCC | ENSP00000294964 |
| BDNF | ENSP00000414303 |
| RARG | ENSP00000332695 |
| DHRS3 | ENSP00000365397 |
| BRK-3 | - |
| SLC34A1 | ENSP00000321424 |
| EDN1 | ENSP00000368683 |
| HRH2 | ENSP00000366506 |
| IFITM5 | ENSP00000372059 |
| SP5 | ENSP00000364430 |
| ENPP1 | ENSP00000354238 |
| CP450CC24 | - |
| BMP4 | ENSP00000245451 |
| MEF2CA | - |
| FGFR2 | ENSP00000410294 |
| FGFR3 | ENSP00000260795 |
| BMP7 | ENSP00000379204 |
| RSPO2 | ENSP00000276659 |
| KAZALD1 | ENSP00000359219 |
| BMP2 | ENSP00000368104 |
| TCF7L2 | ENSP00000358404 |
| GSK3B | ENSP00000324806 |
| RUNX2 | ENSP00000352514 |
| BMPR2 | ENSP00000363708 |
| LXN | ENSP00000264265 |
| EIF2AK3 | ENSP00000307235 |
| LGR4 | ENSP00000368516 |
| NGF | ENSP00000358525 |
| MMP13 | ENSP00000260302 |
| ANO6 | ENSP00000391417 |
| RASSF2 | ENSP00000368684 |
| DML | - |
| CARM1 | ENSP00000325690 |
| HIF1A | ENSP00000338018 |
| CSF3 | ENSP00000225474 |
| SKI | ENSP00000367797 |
| APOA1 | ENSP00000236850 |
| MTSS1 | ENSP00000322804 |
| LEP | ENSP00000312652 |
| CHD7 | ENSP00000392028 |
| THBS2 | ENSP00000355751 |
| MST1 | - |
| CYP26B1 | ENSP00000001146 |
| SHOX | ENSP00000370990 |
| FBXL15 | ENSP00000224862 |
| CYP27B1 | ENSP00000228606 |
| HRH1 | - |
| DLX5 | ENSP00000222598 |
| PTHLHA | - |
| SIRT1 | ENSP00000212015 |
| IGF1 | ENSP00000302665 |
| T[BRACHYURY] | - |
| WNT11 | ENSP00000325526 |
| PAX1 | ENSP00000381499 |
| SHH | ENSP00000297261 |
| NELL1 | ENSP00000298925 |
| PHEX | ENSP00000368682 |
| ATP2B1A | - |
| EGLN1 | ENSP00000355601 |
| GNAS | ENSP00000360141 |
| RIPPLY1 | ENSP00000276173 |
| MEPE | ENSP00000354341 |
| RIPPLY2 | ENSP00000358703 |
| VEGFA | ENSP00000361125 |
| NOV | ENSP00000259526 |
| ACP5 | ENSP00000218758 |
| GLA | ENSP00000218516 |
| EPHA2 | ENSP00000351209 |
| COL27A1B | - |
| COL27A1A | - |
| IL1B | ENSP00000263341 |
| TEK | ENSP00000383977 |
| ASPN | ENSP00000364694 |
| GPNMB | ENSP00000371420 |
| FGF18 | ENSP00000274625 |
| CITED2 | ENSP00000356623 |
| ENTPD5A | - |
| HAS2 | ENSP00000306991 |
| MST1RB | - |
| DUOX2 | - |
| SBNO2 | ENSP00000354733 |
| EFNA2 | ENSP00000215368 |
| MCAM | ENSP00000264036 |
| COL5A3 | ENSP00000264828 |
| ASGR2 | ENSP00000347140 |
| LTF | ENSP00000231751 |
| O55006 | - |
| SFRP2 | ENSP00000274063 |
| CYP24A1 | ENSP00000216862 |
| LRP6 | ENSP00000261349 |
| LRP5 | ENSP00000294304 |
| PAPPA2 | ENSP00000356634 |
| LOC24906 | - |
| ALOX15 | ENSP00000293761 |
| IL6 | ENSP00000258743 |
| IL7 | ENSP00000263851 |
| SPP1 | ENSP00000378517 |
| KDR | ENSP00000263923 |
| PTH1R | ENSP00000321999 |
| RECK | ENSP00000367202 |
| ERCC2 | ENSP00000375809 |
| BMPR1B | ENSP00000264568 |
| SOX9 | ENSP00000245479 |
| CER1 | ENSP00000370297 |
| MITF | ENSP00000295600 |
| MHDAALI18 | - |
| ELF4 | ENSP00000311280 |
| KLF10 | ENSP00000285407 |
| AXIN2 | ENSP00000302625 |
| IFT80 | ENSP00000312778 |
| CLEC3B | ENSP00000296130 |
| IBSP | ENSP00000226284 |
| PTHLH | ENSP00000379213 |
| PHOSPHO1 | ENSP00000406909 |
| MSX1 | ENSP00000372170 |
| MSX2 | ENSP00000239243 |
| LTBP3 | ENSP00000301873 |
| TRAF6 | - |
| PTN | ENSP00000341170 |
| SBDS | ENSP00000246868 |
| MMP9 | ENSP00000361405 |
| OSR2 | ENSP00000297565 |
| TPH1 | ENSP00000250018 |
| GPC3 | ENSP00000359854 |
| STP | - |
| CALCA | ENSP00000331746 |
| HGF | ENSP00000222390 |
| PAPPA | ENSP00000330658 |
| FAM53B | ENSP00000338532 |
| ACTN3 | - |
| PTPRV | - |
| POSTN | ENSP00000369071 |
| NOTCH2 | ENSP00000256646 |
| TFAP2A | ENSP00000368924 |
| HTR1B | ENSP00000358963 |
| TAC1 | ENSP00000321106 |
| WNT16 | ENSP00000222462 |
| MMP2 | ENSP00000219070 |
| DNM3OS | - |
| SATB2 | ENSP00000260926 |

1. Genes for dentin regeneration

| **Gene symbol** | **Ensembl ID** |
| --- | --- |
| STIM1 | ENSP00000300737 |
| ANKH | ENSP00000284268 |
| FOXO1 | ENSP00000368880 |
| TBX1 | ENSP00000331791 |
| HTRA1 | ENSP00000357980 |
| AMELX | ENSP00000370088 |
| PVRL1 | ENSP00000264025 |
| TFAP2A | ENSP00000368924 |
| ASPN | ENSP00000364694 |
| SLC34A1 | ENSP00000321424 |
| KDM6B | ENSP00000254846 |
| MSX2 | ENSP00000239243 |
| FGF10 | ENSP00000264664 |
| DLX3 | - |
| TGFBR1 | ENSP00000364133 |
| HSPB1 | ENSP00000248553 |
| SP7 | ENSP00000302812 |
| BCOR | ENSP00000367705 |
| PPARA | ENSP00000262735 |
| LEF1 | ENSP00000265165 |
| ATP2B1A | - |
| HACD1 | - |
| DICER1 | ENSP00000343745 |
| CNNM4 | ENSP00000366275 |
| FAM20A | - |
| FAM20C | ENSP00000322323 |
| WNT6 | ENSP00000233948 |
| AMTN | ENSP00000341013 |
| COL1A1 | ENSP00000225964 |
| WDR72 | ENSP00000353699 |
| ALPL | ENSP00000363965 |

1. Genes for vessel regeneration

| **Gene symbol** | **Ensembl ID** |
| --- | --- |
| CNTRL | ENSP00000238341 |
| MYH10 | ENSP00000269243 |
| SESTD1 | ENSP00000415332 |
| HECTD1 | ENSP00000382269 |
| NOTCH2 | ENSP00000256646 |
| SUFU | ENSP00000358918 |
| MIN | - |
| EFNB2A | - |
| TBX20 | ENSP00000386170 |
| EFNB2B | - |
| CD34 | ENSP00000310036 |
| SPRED1 | ENSP00000299084 |
| BGN | ENSP00000327336 |
| PCSK5 | ENSP00000365943 |
| DHCR7 | ENSP00000347717 |
| PRICKLE1 | ENSP00000345064 |
| CDX4 | ENSP00000362613 |
| GIT1 | ENSP00000378338 |
| MEIS1 | ENSP00000272369 |
| BR | - |
| RAPGEF3 | ENSP00000395708 |
| MCAMB | - |
| EPHA2 | ENSP00000351209 |
| CTTNL | - |
| DCTN5 | ENSP00000300087 |
| AXL | ENSP00000301178 |
| PPAP2B | - |
| TWSG1A | - |
| TWSG1B | - |
| RBPJ | ENSP00000345206 |
| CRKL | ENSP00000346300 |
| AP2B1 | ENSP00000314414 |
| PTGER4 | ENSP00000302846 |
| MAP3K3 | ENSP00000354927 |
| BIRC5A | - |
| MIB1 | ENSP00000261537 |
| PLEKHH1 | ENSP00000330278 |
| CHM | ENSP00000350386 |
| CTS8 | - |
| BMP10L | - |
| SIX1 | ENSP00000247182 |
| FOXF1 | ENSP00000262426 |
| SMAD5 | - |
| EDN1 | ENSP00000368683 |
| PLXND1 | ENSP00000317128 |
| CDH2 | ENSP00000269141 |
| WNT2 | ENSP00000265441 |
| PROP1 | ENSP00000311290 |
| ARHGEF7B | - |
| DEAR1 | - |
| CDX2 | ENSP00000370408 |
| DBH | ENSP00000376776 |
| STRA6 | ENSP00000326085 |
| CXCR4 | ENSP00000386884 |
| RBPJB | - |
| RBPJA | - |
| ILK | ENSP00000299421 |
| ESX1 | ENSP00000361669 |
| ADAMTS6 | ENSP00000370443 |
| HS6ST1 | ENSP00000259241 |
| JAK2A | - |
| SEC24B | ENSP00000265175 |
| GIPC1 | ENSP00000340698 |
| MDM2 | ENSP00000417281 |
| SETD2 | ENSP00000386759 |
| FGFR1 | ENSP00000380280 |
| HPSE | ENSP00000308107 |
| TBX2 | ENSP00000240328 |
| ADRA1B | ENSP00000306662 |
| MFSD7B | - |
| SOCS3 | ENSP00000330341 |
| DZIP1 | ENSP00000257312 |
| SYK | ENSP00000364898 |
| ARID1A | ENSP00000320485 |
| COL1A2 | ENSP00000297268 |
| TCF7L2 | ENSP00000358404 |
| BAK1 | ENSP00000363591 |
| AHR | ENSP00000242057 |
| ROBO1 | - |
| RUNX1 | ENSP00000300305 |
| ANGPTL3 | ENSP00000360170 |
| CYP26A1 | ENSP00000224356 |
| PKD2 | ENSP00000237596 |
| PRRX1 | ENSP00000356734 |
| CSRP3 | ENSP00000265968 |
| PRRX2 | ENSP00000361547 |
| PKD1 | ENSP00000262304 |
| MFAP2 | ENSP00000364685 |
| DLC | - |
| EPAS1 | ENSP00000263734 |
| NFATC3 | ENSP00000300659 |
| MEIS1B | - |
| SNX5 | ENSP00000366988 |
| ITGA4 | ENSP00000380227 |
| NOS2 | ENSP00000327251 |
| NDNF | ENSP00000369014 |
| ITGA7 | ENSP00000257879 |
| NSDHL | ENSP00000359297 |
| LIF | ENSP00000249075 |
| SMARCA4 | ENSP00000350720 |
| DNM2 | ENSP00000352721 |
| COL3A1 | ENSP00000304408 |
| FLI1A | - |
| NF1A | - |
| NPRL3 | ENSP00000382834 |
| FLI1B | - |
| SOX18 | ENSP00000341815 |
| APOE | ENSP00000252486 |
| APOB | ENSP00000233242 |
| GATAD2A | ENSP00000351552 |
| CHD7 | ENSP00000392028 |
| RASA1 | ENSP00000274376 |
| NOTCH1A | - |
| MKL2 | ENSP00000339086 |
| THBS1 | ENSP00000260356 |
| MIR126A | - |
| AKT1 | ENSP00000270202 |
| NFE2 | ENSP00000312436 |
| NF1 | ENSP00000351015 |
| MTS | - |
| ANGPT2 | ENSP00000314897 |
| BMPR2 | ENSP00000363708 |
| ANGPT1 | ENSP00000297450 |
| NCOA6 | ENSP00000351894 |
| VEGFB | ENSP00000311127 |
| KDRL | - |
| FIGF | ENSP00000297904 |
| ACVRL1 | - |
| NOTCH1B | - |
| NKX2.5 | - |
| ENG | ENSP00000362299 |
| UNC5B | ENSP00000334329 |
| C1QL4L | - |
| COL1A1 | ENSP00000225964 |
| FOXS1 | ENSP00000365145 |
| CCM2 | ENSP00000370503 |
| SHB | ENSP00000366936 |
| PAX6 | ENSP00000368401 |
| VEGFA | ENSP00000361125 |
| COL4A1 | ENSP00000364979 |
| FGF8 | ENSP00000321797 |
| PTENB | - |
| ADIPOR2 | ENSP00000349616 |
| PTENA | - |
| DYNC2H1 | ENSP00000381167 |
| ZMIZ1 | ENSP00000334474 |
| GJA1 | ENSP00000282561 |
| ATP7A | ENSP00000345728 |
| ETV2 | - |
| PSN | - |
| GJA5 | ENSP00000271348 |
| CXXC1B | - |
| APLNR-A | - |
| ITGAV | ENSP00000261023 |
| MEF2C | ENSP00000396219 |
| CBS | ENSP00000344460 |
| VEGFAA | - |
| PRDM1 | ENSP00000358092 |
| IGF1 | ENSP00000302665 |
| NDST1 | ENSP00000261797 |
| HES1 | ENSP00000232424 |
| AGTR1A | - |
| AMOT | ENSP00000361027 |
| FOXC2 | ENSP00000326371 |
| FOXC1 | ENSP00000370256 |
| LUZP1 | ENSP00000363752 |
| EFNB2 | ENSP00000245323 |
| PDGFRB | ENSP00000261799 |
| EPHX2 | ENSP00000369843 |
| VEGFC | ENSP00000280193 |
| 2410089E03RIK | - |
| NEDD4 | ENSP00000345530 |
| SMAD6 | ENSP00000288840 |
| SMAD7 | ENSP00000262158 |
| GATA2A | - |
| SH3GL3B | - |
| MAP2K1 | ENSP00000302486 |
| MAP3K7 | ENSP00000358335 |
| SMO | ENSP00000249373 |
| SPRY2 | ENSP00000366306 |
| CCR2 | ENSP00000292301 |
| TBX1 | ENSP00000331791 |
| TBX6 | ENSP00000279386 |
| NOS3 | ENSP00000297494 |
| NF1B | - |
| FLT4 | ENSP00000261937 |
| RAPGEF2 | ENSP00000264431 |
| HPGD | ENSP00000296522 |
| FLT1 | ENSP00000282397 |
| SNX17 | ENSP00000233575 |
| HSP90AB1 | ENSP00000325875 |
| CYP1B1 | ENSP00000260630 |
| FZD5 | ENSP00000354607 |
| FOXH1 | ENSP00000366534 |
| ZBTB14 | ENSP00000349503 |
| JMJD6 | ENSP00000394085 |
| DLX3 | - |
| LTBP1 | ENSP00000346467 |
| HOXA13 | ENSP00000222753 |
| TLL1 | ENSP00000061240 |
| NR2F2 | - |
| HSPA12B | ENSP00000254963 |
| MAPK1 | ENSP00000215832 |
| FOSL1 | ENSP00000310170 |
| TNNT2A | - |
| RBM15 | ENSP00000358799 |
| CITED2 | ENSP00000356623 |
| CALCRLA | - |
| FGF10 | ENSP00000264664 |
| PTHLHA | - |
| HAS2 | ENSP00000306991 |
| PRP | - |
| TGFBR3 | ENSP00000212355 |
| LAMA1 | ENSP00000374309 |
| TGFBR1 | ENSP00000364133 |
| LAMA4 | ENSP00000230538 |
| CCBE1 | ENSP00000404464 |
| SYNB | - |
| MTMR8 | ENSP00000363985 |
| MCAM | ENSP00000264036 |
| PDGFBA | - |
| COL5A1 | ENSP00000360882 |
| DLL4 | ENSP00000249749 |
| HIF1A | ENSP00000338018 |
| SERPINF2 | ENSP00000321853 |
| GJA4 | ENSP00000343676 |
| MIR27B | - |
| HEY2 | ENSP00000357348 |
| PROX1 | ENSP00000261454 |
| NOL3 | ENSP00000268605 |
| HEY1 | ENSP00000338272 |
| LRP2 | ENSP00000263816 |
| SHH | ENSP00000297261 |
| SEMA3C | ENSP00000265361 |
| LRP1 | ENSP00000243077 |
| LLGL2 | ENSP00000376333 |
| LRP5 | ENSP00000294304 |
| NAA35 | ENSP00000354972 |
| LMO2 | ENSP00000257818 |
| NTN4 | ENSP00000340998 |
| FOXO1 | ENSP00000368880 |
| CFC1 | ENSP00000259216 |
| PLG | ENSP00000308938 |
| FUZ | ENSP00000313309 |
| PLCD1 | - |
| HOXA3 | ENSP00000324884 |
| HOXA1 | ENSP00000343246 |
| FZD4 | - |
| ELN | ENSP00000252034 |
| NRP1 | ENSP00000265371 |
| FOXC1B | - |
| GDF6A | - |
| AMOTL1 | ENSP00000387739 |
| AGGF1 | ENSP00000316109 |
| TGFB2 | ENSP00000355896 |
| PTK2 | ENSP00000341189 |
| FBXW8 | ENSP00000310686 |
| PTK7 | ENSP00000230419 |
| STAB2 | ENSP00000373539 |
| PTH1RA | - |
| BMPER | ENSP00000297161 |
| JUNB | ENSP00000303315 |
| ACVR2B | ENSP00000340361 |
| BAX | ENSP00000293288 |
| AIMP1 | ENSP00000378191 |
| SPINT1 | ENSP00000342098 |
| AGT | ENSP00000355627 |
| PLCG1 | ENSP00000244007 |
| BMP10 | ENSP00000295379 |
| MYOCD | ENSP00000341835 |
| HAND2 | ENSP00000352565 |
| PITX2 | ENSP00000304169 |
| SRF | ENSP00000265354 |
| LOX | ENSP00000231004 |
| PDE2A | ENSP00000334910 |
| PDCD10A | - |
| SOX7 | ENSP00000301921 |
| HSP70L | - |
| PDCD10B | - |
| STK25B | - |
| KIF7 | ENSP00000377934 |
| TAL1 | ENSP00000294339 |
| GDF2 | ENSP00000249598 |
| PAK2A | - |
| TFAP2B | ENSP00000377265 |
| TBX3 | ENSP00000257566 |
| MEGF8 | ENSP00000334219 |
| PTPRJA | - |
| GLI3 | - |
| ETS1 | ENSP00000376436 |
| MED14 | ENSP00000323720 |
| ALDH1A2 | ENSP00000249750 |
| ATG5 | ENSP00000343313 |
| OVOL2 | ENSP00000278780 |
| JAG1 | ENSP00000254958 |
| EYA1 | ENSP00000342626 |
| MYO6A | - |
| PDGFB | ENSP00000330382 |
| NTN1A | - |
| CYR61 | ENSP00000398736 |
| HEG1 | ENSP00000311502 |
| SPHK1 | ENSP00000313681 |
| LOXL1 | ENSP00000261921 |
| EPHB2A | - |
| PTPRJB.1 | - |
| SLV | - |
| PSEN1 | ENSP00000326366 |
| OSR1 | ENSP00000272223 |
| RASA1A | - |
| GPC3 | ENSP00000359854 |
| RAPGEF1 | ENSP00000361264 |
| EPHB4A | - |
| AGTR1B | - |
| KRIT1 | ENSP00000344668 |
| TGM2 | ENSP00000355330 |
| MMP21 | ENSP00000357798 |
| FOXN1 | ENSP00000226247 |
| PLCD3 | ENSP00000313731 |
| SPHK2 | ENSP00000245222 |
| TIE1 | ENSP00000361554 |
| CFI | ENSP00000378130 |
| TAB1 | ENSP00000216160 |
| UNC45A | ENSP00000407487 |
| NOTCH1 | ENSP00000277541 |
| ITGB8 | ENSP00000222573 |
| NOTCH3 | ENSP00000263388 |
| SEMA3AA | - |
| PTPRB | ENSP00000334928 |
| FOXC1A | - |
| PAK2B | - |
| BMP4 | ENSP00000245451 |
| KAT6A | ENSP00000265713 |
| CRIM1 | ENSP00000280527 |
| FOLR1 | ENSP00000308137 |
| WNT11 | ENSP00000325526 |
| ANGPT2B | - |

1. Genes for nerve regeneration

| Gene symbol | Ensembl ID |
| --- | --- |
| LIF | ENSP00000249075 |
| AGER | ENSP00000364217 |
| MIR138-1 | - |
| AKT1 | ENSP00000270202 |
| AKT2 | ENSP00000375892 |
| L1CAM | ENSP00000359074 |
| SCN9A | ENSP00000386306 |
| FAM168B | ENSP00000374565 |
| BDNF | ENSP00000414303 |
| CCL13 | ENSP00000225844 |
| SEMA4D | ENSP00000343418 |
| LGALS1 | ENSP00000215909 |
| CAV1 | ENSP00000339191 |
| RARB | ENSP00000332296 |
| NCAN | ENSP00000252575 |
| GRIA3 | ENSP00000360302 |
| NDST1 | ENSP00000261797 |
| GHRL | ENSP00000335074 |
| MYCBP2 | ENSP00000349892 |
| NTRK2 | ENSP00000277120 |
| MICAL1 | ENSP00000351664 |
| MICAL2 | ENSP00000256194 |
| RTN4R | ENSP00000043402 |
| RELN | ENSP00000392423 |
| ITGB4 | ENSP00000200181 |
| SLC12A2 | ENSP00000262461 |
| THY1 | ENSP00000284240 |
| CDH2 | ENSP00000269141 |
| CDKN1B | ENSP00000228872 |
| MYT1 | ENSP00000327465 |
| ERBB2 | ENSP00000269571 |
| SEMA3A | ENSP00000265362 |
| IGF1R | ENSP00000268035 |
| CDKN2A | ENSP00000355153 |
| ERBB4 | ENSP00000342235 |
| GIP | ENSP00000350005 |
| IFNG | ENSP00000229135 |
| NFIL3 | ENSP00000297689 |
| TGIF1 | ENSP00000327959 |
| TF | ENSP00000264998 |
| FLRT3 | ENSP00000339912 |
| LPL | ENSP00000309757 |
| DOCK3 | ENSP00000266037 |
| IFRD1 | ENSP00000005558 |
| GATA6 | ENSP00000269216 |
| NEUROD6 | ENSP00000297142 |
| MDM2 | ENSP00000417281 |
| SEMA7A | ENSP00000261918 |
| FGFR1 | ENSP00000380280 |
| MAPK14 | ENSP00000229794 |
| FN1 | ENSP00000346839 |
| SOCS3 | ENSP00000330341 |
| MBP | ENSP00000348273 |
| ROBO2 | ENSP00000417164 |
| PRPH | ENSP00000257860 |
| GSK3B | ENSP00000324806 |
| TLR2 | ENSP00000260010 |
| PTEN | ENSP00000361021 |
| SYP | ENSP00000263233 |
| MAG | - |
| DIXDC1 | ENSP00000394352 |
| AKAP12 | ENSP00000253332 |
| MYO5A | ENSP00000382177 |
| GIPC1 | ENSP00000340698 |
| ELAVL4 | ENSP00000349594 |
| IFT43 | ENSP00000238628 |
| NGF | ENSP00000358525 |
| OCM | ENSP00000242104 |
| TRPC4 | ENSP00000369003 |
| RASD1 | ENSP00000225688 |
| EPHB3 | ENSP00000332118 |
| NRSN1 | ENSP00000367752 |
| CDKN1A | ENSP00000244741 |
| RAPGEF3 | ENSP00000395708 |
| ITGA4 | ENSP00000380227 |
| KAT2B | ENSP00000263754 |
| ITGA6 | ENSP00000386896 |
| ITGA7 | ENSP00000257879 |
| ULK1 | ENSP00000324560 |
| RNH1 | ENSP00000346402 |
| CHL1 | ENSP00000256509 |
| NDRG1 | ENSP00000319977 |
| TNFRSF12A | ENSP00000326737 |
| IL1B | ENSP00000263341 |
| TNFRSF19 | ENSP00000371693 |
| HN1 | ENSP00000348316 |
| APOD | ENSP00000345179 |
| APOE | ENSP00000252486 |
| LILRB3 | - |
| GAP43 | ENSP00000377372 |
| TLR3 | ENSP00000296795 |
| TUBA1C | ENSP00000301072 |
| SOX11 | ENSP00000322568 |
| JUN | ENSP00000360266 |
| PPARGC1A | ENSP00000264867 |
| SOX17 | ENSP00000297316 |
| PLEKHB1 | ENSP00000346127 |
| STK25 | ENSP00000325748 |
| CIT | ENSP00000261833 |
| PIK3R1 | ENSP00000274335 |
| MIR124-1 | - |
| B4GALT1 | ENSP00000369055 |
| REG3G | ENSP00000272324 |
| B4GALT5 | ENSP00000360776 |
| MT1A | ENSP00000290705 |
| NRTN | ENSP00000302648 |
| REG3A | ENSP00000304311 |
| NEFL | - |
| SIRT1 | ENSP00000212015 |
| IGF1 | ENSP00000302665 |
| DPYSL2 | ENSP00000309539 |
| GFRA3 | ENSP00000274721 |
| TP63 | ENSP00000264731 |
| EFEMP1 | ENSP00000347596 |
| MAP1B | ENSP00000296755 |
| PPARG | ENSP00000287820 |
| PAX6 | ENSP00000368401 |
| VEGFA | ENSP00000361125 |
| CASP2 | ENSP00000312664 |
| SHH | ENSP00000297261 |
| TTR | ENSP00000237014 |
| RHOA | ENSP00000400175 |
| CALU | ENSP00000249364 |
| KIF3C | ENSP00000264712 |
| EPOR | ENSP00000222139 |
| PMP22 | ENSP00000308937 |
| TP53 | ENSP00000269305 |
| POSTN | ENSP00000369071 |
| GPC1 | ENSP00000264039 |
| SDC1 | ENSP00000254351 |
| VAV3 | ENSP00000359073 |
| ST8SIA4 | ENSP00000231461 |
| PSAP | ENSP00000378394 |
| CSPG4 | ENSP00000312506 |
| PXDN | ENSP00000252804 |
| FLOT2 | ENSP00000378368 |
| UBE2B | ENSP00000265339 |
| APOA5 | ENSP00000227665 |
| GAL | ENSP00000265643 |
| IL17RD | ENSP00000296318 |
| MPZ | ENSP00000353634 |
| CREBBP | ENSP00000262367 |
| MIRLET7A1 | - |
| WNT3A | ENSP00000284523 |
| EFNB3 | ENSP00000226091 |
| PDGFRA | ENSP00000257290 |
| HDAC5 | ENSP00000225983 |
| HDAC6 | ENSP00000334061 |
| EPHA4 | ENSP00000281821 |
| SMAD1 | ENSP00000305769 |
| RET | ENSP00000347942 |
| SMO | ENSP00000249373 |
| FABP5 | ENSP00000297258 |
| MAPK10 | ENSP00000352157 |
| ADCYAP1 | - |
| CEND1 | ENSP00000328336 |
| NCAM1 | ENSP00000318472 |
| SKP2 | ENSP00000274255 |
| RGMA | ENSP00000330005 |
| MDK | ENSP00000352852 |
| TICAM2 | ENSP00000386341 |
| SET | ENSP00000361777 |
| STAT3 | ENSP00000264657 |
| PDLIM1 | ENSP00000360305 |
| SPDYA | ENSP00000335628 |
| MIR206 | - |
| FGF2 | ENSP00000264498 |
| MAPK3 | ENSP00000263025 |
| MICAL3 | ENSP00000416015 ENSP00000414846 |
| HNRNPK | ENSP00000365439 |
| ICAM1 | ENSP00000264832 |
| SPTAN1 | ENSP00000361824 |
| C6 | ENSP00000263413 |
| ERN1 | ENSP00000401445 |
| ARTN | ENSP00000391998 |
| BCAN | ENSP00000331210 |
| NTF3 | ENSP00000397297 |
| NTF4 | ENSP00000391622 ENSP00000301411 |
| IL17A | ENSP00000344192 |
| EGFR | ENSP00000275493 |
| TLR4 | ENSP00000363089 |
| MCAM | ENSP00000264036 |
| FERMT1 | ENSP00000217289 |
| MTOR | ENSP00000354558 |
| RIT1 | ENSP00000357306 |
| PLCD1 | - |
| LGALS3 | ENSP00000254301 |
| CLU | ENSP00000315130 |
| AKR1B1 | ENSP00000285930 |
| EP300 | ENSP00000263253 |
| TSPO | ENSP00000328973 |
| LRP2 | ENSP00000263816 |
| FOXO3 | ENSP00000339527 |
| NDE1 | ENSP00000345892 |
| NEUROD1 | ENSP00000295108 |
| OMG | ENSP00000247271 |
| NTN1 | ENSP00000173229 |
| PROK2 | ENSP00000295619 |
| ROCK1 | ENSP00000382697 |
| ID2 | ENSP00000234091 |
| GJC3 | ENSP00000325775 |
| CDH4 | ENSP00000353656 |
| IL6 | ENSP00000258743 |
| MAPK1 | ENSP00000215832 |
| PALLD | ENSP00000261509 |
| STX12 | ENSP00000363054 |
| SPP1 | ENSP00000378517 |
| ARG1 | ENSP00000357066 |
| CADM1 | ENSP00000329797 |
| NEU3 | ENSP00000294064 |
| TGFB1 | ENSP00000221930 |
| VAV2 | ENSP00000360916 |
| TRIM71 | ENSP00000373272 |
| ERBB3 | ENSP00000267101 |
| DHFR | ENSP00000396308 |
| PLAUR | ENSP00000339328 |
| LRP1 | ENSP00000243077 |
| VCAN | ENSP00000265077 |
| BAX | ENSP00000293288 |
| PTPRZ1 | ENSP00000377047 |
| CTNNB1 | ENSP00000344456 |
| APOA4 | ENSP00000350425 |
| NGFR | ENSP00000172229 |
| SPRR1A | ENSP00000307340 |
| CHST3 | ENSP00000362207 |
| FCGR3A | ENSP00000356946 |
| GIPR | ENSP00000263281 |
| CEBPB | ENSP00000305422 |
| RHO | ENSP00000296271 |
| CRABP2 | ENSP00000357204 |
| CEBPD | ENSP00000386165 |
| GJB1 | ENSP00000354900 |
| MT3 | ENSP00000200691 |
| NEFH | ENSP00000311997 |
| APOA1 | ENSP00000236850 |
| SMAD2 | ENSP00000262160 |
| KLF4 | ENSP00000363804 |
| SLIT3 | ENSP00000332164 |
| SLIT2 | ENSP00000273739 |
| SLIT1 | ENSP00000266058 |
| NRG1 | ENSP00000349275 |
| ZBP1 | ENSP00000360215 |
| GFAP | ENSP00000253408 |
| KLF9 | ENSP00000366330 |
| BRAF | ENSP00000288602 |
| ITGB1 | ENSP00000364094 |
| CNTF | ENSP00000355370 |
| MATN2 | ENSP00000254898 |
| RTN3 | ENSP00000344106 |
| RAC1 | ENSP00000348461 |
| RTN4 | ENSP00000337838 |
| NINJ1 | ENSP00000364595 |
| PLAT | ENSP00000220809 |
| CRYBB2 | - |
| GORAB | ENSP00000356737 |
| CNTN1 | ENSP00000325660 |
| SKIL | ENSP00000259119 |
| TNC | ENSP00000265131 |
| NDEL1 | ENSP00000333982 |
| P2RY2 | ENSP00000310305 |
| ZDHHC23 | ENSP00000330485 |
| NRP1 | ENSP00000265371 |
| NRP2 | ENSP00000353582 |
| CASP6 | ENSP00000265164 |
| VAMP3 | ENSP00000054666 |
| VAMP2 | ENSP00000314214 |
| PTPRS | ENSP00000349932 |
| SPAST | ENSP00000320885 |
| PXN | ENSP00000228307 |
| MOG | ENSP00000390785 ENSP00000397101 ENSP00000259891 ENSP00000391898 ENSP00000395005 ENSP00000390632 ENSP00000366095 |
| ADM | ENSP00000278175 |
| NOTCH1 | ENSP00000277541 |
| ROCK2 | ENSP00000317985 |
| ACKR3 | - |
| MAP3K12 | ENSP00000267079 |
| FCGR3B | ENSP00000294800 |
| GDNF | ENSP00000317145 |
| MMP9 | ENSP00000361405 |
| CARTPT | ENSP00000296777 |
| CALCB | ENSP00000346017 |
| KCNK3 | ENSP00000306275 |
| MMP2 | ENSP00000219070 |
| ATF3 | ENSP00000344352 |
| BCL2 | ENSP00000329623 |
