# Supplementary material for: Network-Based Method for Identifying Co-Regeneration Genes in Bone, Dentin, Nerve and Vessel Tissues
Source: Genes (Basel). 2017 Oct 2;8(10):252. doi: 10.3390/genes8100252 (PMC5664102; doi:10.3390/genes8100252)
Supplement: Supplementary file 1 [file genes-08-00252-s001.zip › Table_S2.docx]

**Table S2.** Genes obtained by the network-based method and their measurements.

- Betweenness is defined as the number of shortest paths containing a gene.
- Permutation FDR is defined as the proportion of the randomly produced set pairs for which betweenness was greater than that of actual pair (cf. Eq. 1). A high permutation FDR means the corresponding gene can be produced by numerous gene set pairs, suggesting it is not specific for the actual gene set pair.
- Betweenness ratio is defined as the ratio of number of shortest paths containing the gene and the total number of possible shortest paths (c. Eq. 3). This measurement can evaluate the importance of a candidate gene. If a candidate gene was assigned a high betweenness ratio, it is more likely to be a co-regeneration gene.
- Min-Max interaction score is defined as the minimum of the maximum interaction score between the gene and genes of two tissues (cf. Eq. 4). A candidate gene with a high Min-Max interaction score is more likely to be a co-regeneration gene because it is highly related to at least one gene of two tissues.

1. 244 candidate co-regeneration genes of bone and dentine

| **Ensembl ID** | **Gene symbol** | **Betweenness** | **Permutation FDR** | **Betweenness ratio** | **Min-Max interaction score** |
| --- | --- | --- | --- | --- | --- |
| ENSP00000231572 | RARS | 122 | <0.001 | 0.037037 | 865 |
| ENSP00000355890 | EPRS | 121 | <0.001 | 0.036733 | 0 |
| ENSP00000360483 | TMEM48 | 121 | 0.001 | 0.036733 | 0 |
| ENSP00000295119 | NUP35 | 121 | 0.001 | 0.036733 | 0 |
| ENSP00000310668 | NUP93 | 121 | 0.003 | 0.036733 | 0 |
| ENSP00000325448 | KARS | 121 | 0.004 | 0.036733 | 0 |
| ENSP00000223029 | AIMP2 | 121 | 0.005 | 0.036733 | 0 |
| ENSP00000370526 | ARSE | 1 | 0.006 | 0.000304 | 0 |
| ENSP00000216225 | RBX1 | 41 | 0.008 | 0.012447 | 193 |
| ENSP00000261267 | LYZ | 27 | 0.009 | 0.008197 | 669 |
| ENSP00000396127 | RAN | 141 | 0.01 | 0.042805 | 374 |
| ENSP00000348577 | RANGAP1 | 141 | 0.01 | 0.042805 | 0 |
| ENSP00000230882 | GHR | 122 | 0.011 | 0.037037 | 675 |
| ENSP00000339428 | SOCS2 | 6 | 0.011 | 0.001821 | 0 |
| ENSP00000311697 | FGF5 | 27 | 0.011 | 0.008197 | 273 |
| ENSP00000260228 | MMP20 | 122 | 0.011 | 0.037037 | 157 |
| ENSP00000313809 | AMBN | 122 | 0.012 | 0.037037 | 583 |
| ENSP00000299335 | COX11 | 122 | 0.013 | 0.037037 | 303 |
| ENSP00000255390 | SCO1 | 122 | 0.013 | 0.037037 | 0 |
| ENSP00000035307 | CHPF2 | 26 | 0.014 | 0.007893 | 0 |
| ENSP00000331736 | SELE | 27 | 0.018 | 0.008197 | 430 |
| ENSP00000361658 | NUP188 | 27 | 0.019 | 0.008197 | 0 |
| ENSP00000299855 | MMP3 | 27 | 0.02 | 0.008197 | 424 |
| ENSP00000357392 | EFNA1 | 27 | 0.02 | 0.008197 | 160 |
| ENSP00000305416 | S1PR1 | 1 | 0.021 | 0.000304 | 353 |
| ENSP00000362649 | HDAC1 | 30 | 0.022 | 0.009107 | 967 |
| ENSP00000351605 | FZD6 | 27 | 0.025 | 0.008197 | 878 |
| ENSP00000269593 | IGFBP4 | 27 | 0.025 | 0.008197 | 260 |
| ENSP00000262435 | SMURF2 | 3 | 0.027 | 0.000911 | 933 |
| ENSP00000384675 | SOS1 | 9 | 0.03 | 0.002732 | 299 |
| ENSP00000368174 | MCM8 | 26 | 0.031 | 0.007893 | 540 |
| ENSP00000332353 | PTCH1 | 155 | 0.033 | 0.047055 | 878 |
| ENSP00000264914 | ARSB | 1 | 0.035 | 0.000304 | 201 |
| ENSP00000271688 | CERS2 | 27 | 0.037 | 0.008197 | 318 |
| ENSP00000342392 | MESP2 | 54 | 0.038 | 0.016393 | 269 |
| ENSP00000285968 | NUP205 | 4 | 0.039 | 0.001214 | 0 |
| ENSP00000256078 | KRAS | 24 | 0.043 | 0.007286 | 433 |
| ENSP00000168712 | FGF4 | 3 | 0.043 | 0.000911 | 920 |
| ENSP00000260605 | DYNC2LI1 | 27 | 0.044 | 0.008197 | 0 |
| ENSP00000233813 | IGFBP5 | 27 | 0.046 | 0.008197 | 340 |
| ENSP00000290158 | KPNB1 | 117 | 0.047 | 0.035519 | 0 |
| ENSP00000262077 | NUP153 | 117 | 0.047 | 0.035519 | 229 |
| ENSP00000229179 | NUP107 | 4 | 0.05 | 0.001214 | 0 |
| ENSP00000339992 | MYB | 27 | 0.051 | 0.008197 | 965 |
| ENSP00000355261 | SMG5 | 2 | 0.052 | 0.000607 | 0 |
| ENSP00000162749 | TNFRSF1A | 136 | 0.055 | 0.041287 | 347 |
| ENSP00000316054 | DVL3 | 26 | 0.055 | 0.007893 | 288 |
| ENSP00000189444 | NFKB2 | 15 | 0.057 | 0.004554 | 168 |
| ENSP00000164227 | BCL3 | 27 | 0.057 | 0.008197 | 229 |
| ENSP00000322788 | MMP1 | 1 | 0.058 | 0.000304 | 414 |
| ENSP00000361066 | NCOA3 | 31 | 0.058 | 0.009411 | 912 |
| ENSP00000350878 | S1PR3 | 26 | 0.058 | 0.007893 | 388 |
| ENSP00000351908 | MAP3K5 | 26 | 0.059 | 0.007893 | 219 |
| ENSP00000292408 | FGFR4 | 27 | 0.062 | 0.008197 | 925 |
| ENSP00000372793 | LTA | 17 | 0.063 | 0.005161 | 227 |
| ENSP00000218388 | TIMP1 | 148 | 0.066 | 0.04493 | 505 |
| ENSP00000324897 | UBE2I | 142 | 0.066 | 0.043109 | 644 |
| ENSP00000222725 | LFNG | 54 | 0.066 | 0.016393 | 285 |
| ENSP00000332973 | SMAD3 | 360 | 0.067 | 0.10929 | 875 |
| ENSP00000316244 | HTR1A | 27 | 0.068 | 0.008197 | 0 |
| ENSP00000337103 | CHAT | 27 | 0.071 | 0.008197 | 307 |
| ENSP00000294172 | NXF1 | 29 | 0.079 | 0.008804 | 258 |
| ENSP00000247668 | TRAF2 | 153 | 0.079 | 0.046448 | 654 |
| ENSP00000316032 | NUP98 | 1 | 0.09 | 0.000304 | 227 |
| ENSP00000287934 | FZD1 | 120 | 0.09 | 0.03643 | 896 |
| ENSP00000352400 | NUP214 | 24 | 0.091 | 0.007286 | 210 |
| ENSP00000334122 | FGF3 | 26 | 0.092 | 0.007893 | 906 |
| ENSP00000376076 | SUMO1 | 6 | 0.095 | 0.001821 | 619 |
| ENSP00000268058 | PML | 2 | 0.096 | 0.000607 | 963 |
| ENSP00000378529 | FZR1 | 1 | 0.098 | 0.000304 | 0 |
| ENSP00000306920 | GLB1 | 13 | 0.1 | 0.003947 | 516 |
| ENSP00000373614 | SELPLG | 27 | 0.1 | 0.008197 | 340 |
| ENSP00000419692 | RXRA | 82 | 0.101 | 0.024894 | 964 |
| ENSP00000293549 | WNT1 | 26 | 0.102 | 0.007893 | 541 |
| ENSP00000308208 | MMP14 | 27 | 0.102 | 0.008197 | 816 |
| ENSP00000276420 | DOK2 | 18 | 0.102 | 0.005464 | 0 |
| ENSP00000339151 | IKBKB | 6 | 0.112 | 0.001821 | 749 |
| ENSP00000295731 | IHH | 50 | 0.114 | 0.015179 | 900 |
| ENSP00000228682 | GLI1 | 27 | 0.117 | 0.008197 | 412 |
| ENSP00000229307 | NANOG | 24 | 0.12 | 0.007286 | 563 |
| ENSP00000227507 | CCND1 | 121 | 0.121 | 0.036733 | 946 |
| ENSP00000305692 | GAA | 14 | 0.122 | 0.00425 | 833 |
| ENSP00000226574 | NFKB1 | 12 | 0.126 | 0.003643 | 813 |
| ENSP00000317272 | MET | 54 | 0.126 | 0.016393 | 842 |
| ENSP00000344115 | CDH5 | 2 | 0.13 | 0.000607 | 346 |
| ENSP00000383623 | MLLT4 | 122 | 0.131 | 0.037037 | 394 |
| ENSP00000278385 | CD44 | 41 | 0.134 | 0.012447 | 936 |
| ENSP00000302150 | PRL | 31 | 0.134 | 0.009411 | 842 |
| ENSP00000302728 | GUSB | 13 | 0.135 | 0.003947 | 427 |
| ENSP00000363822 | AR | 47 | 0.147 | 0.014268 | 902 |
| ENSP00000354876 | MT-CO2 | 122 | 0.147 | 0.037037 | 410 |
| ENSP00000369442 | KL | 13 | 0.151 | 0.003947 | 439 |
| ENSP00000262643 | CCNE1 | 1 | 0.158 | 0.000304 | 206 |
| ENSP00000330393 | LEPR | 1 | 0.159 | 0.000304 | 867 |
| ENSP00000338799 | IL6ST | 27 | 0.163 | 0.008197 | 900 |
| ENSP00000363071 | DES | 27 | 0.165 | 0.008197 | 466 |
| ENSP00000386259 | NEB | 27 | 0.165 | 0.008197 | 207 |
| ENSP00000254066 | RARA | 2 | 0.172 | 0.000607 | 902 |
| ENSP00000339007 | GRB2 | 95 | 0.178 | 0.02884 | 374 |
| ENSP00000262768 | TIMP2 | 27 | 0.183 | 0.008197 | 366 |
| ENSP00000360286 | RAE1 | 27 | 0.187 | 0.008197 | 218 |
| ENSP00000398698 | TNF | 121 | 0.191 | 0.036733 | 942 |
| ENSP00000216797 | NFKBIA | 1 | 0.201 | 0.000304 | 867 |
| ENSP00000368438 | PCNA | 26 | 0.205 | 0.007893 | 565 |
| ENSP00000361418 | IPO13 | 8 | 0.209 | 0.002429 | 0 |
| ENSP00000170630 | IL4R | 19 | 0.214 | 0.005768 | 249 |
| ENSP00000329357 | SP1 | 3 | 0.215 | 0.000911 | 922 |
| ENSP00000267163 | RB1 | 19 | 0.219 | 0.005768 | 653 |
| ENSP00000262238 | YY1 | 13 | 0.22 | 0.003947 | 943 |
| ENSP00000228837 | FGF6 | 195 | 0.22 | 0.059199 | 908 |
| ENSP00000351665 | CLIP1 | 27 | 0.226 | 0.008197 | 333 |
| ENSP00000354791 | DCTN1 | 27 | 0.226 | 0.008197 | 229 |
| ENSP00000348827 | THRB | 1 | 0.235 | 0.000304 | 905 |
| ENSP00000359206 | BTRC | 4 | 0.246 | 0.001214 | 319 |
| ENSP00000276201 | UPF3B | 3 | 0.26 | 0.000911 | 0 |
| ENSP00000232014 | BCL6 | 122 | 0.261 | 0.037037 | 871 |
| ENSP00000263686 | SELP | 27 | 0.262 | 0.008197 | 396 |
| ENSP00000264554 | SHC2 | 142 | 0.262 | 0.043109 | 260 |
| ENSP00000350616 | DDC | 27 | 0.262 | 0.008197 | 306 |
| ENSP00000287497 | ITGAM | 27 | 0.269 | 0.008197 | 306 |
| ENSP00000237837 | FGF23 | 67 | 0.271 | 0.02034 | 913 |
| ENSP00000216223 | IL2RB | 8 | 0.282 | 0.002429 | 291 |
| ENSP00000367207 | MYC | 4 | 0.283 | 0.001214 | 851 |
| ENSP00000370571 | TH | 27 | 0.288 | 0.008197 | 419 |
| ENSP00000364000 | COL5A2 | 27 | 0.288 | 0.008197 | 914 |
| ENSP00000262803 | UPF1 | 2 | 0.294 | 0.000607 | 205 |
| ENSP00000356070 | MAPKAPK2 | 122 | 0.295 | 0.037037 | 460 |
| ENSP00000344468 | SDC3 | 27 | 0.295 | 0.008197 | 800 |
| ENSP00000344818 | UBC | 1629 | 0.3 | 0.494536 | 999 |
| ENSP00000259371 | DAB2IP | 26 | 0.304 | 0.007893 | 0 |
| ENSP00000266987 | TARBP2 | 1 | 0.311 | 0.000304 | 507 |
| ENSP00000361850 | PLAU | 2 | 0.314 | 0.000607 | 380 |
| ENSP00000256443 | CDK7 | 27 | 0.314 | 0.008197 | 0 |
| ENSP00000306245 | FOS | 107 | 0.316 | 0.032483 | 565 |
| ENSP00000369050 | CYP1A1 | 53 | 0.319 | 0.01609 | 918 |
| ENSP00000256474 | VHL | 339 | 0.319 | 0.102914 | 165 |
| ENSP00000311430 | RPL4 | 27 | 0.326 | 0.008197 | 567 |
| ENSP00000256857 | GRP | 3 | 0.33 | 0.000911 | 290 |
| ENSP00000239223 | DUSP1 | 6 | 0.334 | 0.001821 | 344 |
| ENSP00000309845 | HRAS | 130 | 0.337 | 0.039466 | 467 |
| ENSP00000287598 | BUB1B | 8 | 0.34 | 0.002429 | 0 |
| ENSP00000256442 | CCNB1 | 205 | 0.349 | 0.062234 | 749 |
| ENSP00000233057 | EIF2AK2 | 1 | 0.352 | 0.000304 | 170 |
| ENSP00000296871 | CSF2 | 1 | 0.354 | 0.000304 | 561 |
| ENSP00000384053 | CSF2RB | 1 | 0.354 | 0.000304 | 0 |
| ENSP00000296581 | LSM6 | 1 | 0.355 | 0.000304 | 0 |
| ENSP00000361423 | ABL1 | 1 | 0.371 | 0.000304 | 319 |
| ENSP00000354554 | MT-CYB | 122 | 0.374 | 0.037037 | 344 |
| ENSP00000293379 | ITGA5 | 293 | 0.375 | 0.08895 | 919 |
| ENSP00000206249 | ESR1 | 330 | 0.376 | 0.100182 | 958 |
| ENSP00000354961 | MT-ND4 | 122 | 0.378 | 0.037037 | 274 |
| ENSP00000367408 | CASK | 21 | 0.379 | 0.006375 | 307 |
| ENSP00000280665 | DCP1B | 2 | 0.379 | 0.000607 | 0 |
| ENSP00000231487 | SKP1 | 61 | 0.38 | 0.018519 | 605 |
| ENSP00000341551 | SMAD4 | 242 | 0.388 | 0.073467 | 995 |
| ENSP00000336790 | ATF4 | 26 | 0.393 | 0.007893 | 894 |
| ENSP00000343204 | JAK1 | 8 | 0.404 | 0.002429 | 902 |
| ENSP00000256383 | EIF2S1 | 1 | 0.408 | 0.000304 | 245 |
| ENSP00000351407 | ARNT | 51 | 0.41 | 0.015483 | 0 |
| ENSP00000352980 | HIST1H4A | 3 | 0.411 | 0.000911 | 348 |
| ENSP00000226218 | SEBOX | 12 | 0.412 | 0.003643 | 499 |
| ENSP00000278616 | ATM | 1 | 0.416 | 0.000304 | 659 |
| ENSP00000320940 | NCOA1 | 119 | 0.416 | 0.036126 | 995 |
| ENSP00000348708 | UPF2 | 5 | 0.418 | 0.001518 | 0 |
| ENSP00000209728 | CDC6 | 26 | 0.423 | 0.007893 | 0 |
| ENSP00000340944 | PTPN11 | 57 | 0.429 | 0.017304 | 281 |
| ENSP00000371067 | JAK2 | 169 | 0.441 | 0.051305 | 379 |
| ENSP00000284811 | TCEB1 | 6 | 0.447 | 0.001821 | 0 |
| ENSP00000264110 | ATF2 | 116 | 0.456 | 0.035216 | 379 |
| ENSP00000362820 | SRSF3 | 2 | 0.476 | 0.000607 | 0 |
| ENSP00000354394 | STAT1 | 22 | 0.479 | 0.006679 | 808 |
| ENSP00000295897 | ALB | 26 | 0.481 | 0.007893 | 879 |
| ENSP00000003084 | CFTR | 192 | 0.488 | 0.058288 | 429 |
| ENSP00000310596 | LSM1 | 2 | 0.492 | 0.000607 | 210 |
| ENSP00000300093 | PLK1 | 27 | 0.495 | 0.008197 | 305 |
| ENSP00000307046 | SDC2 | 21 | 0.507 | 0.006375 | 805 |
| ENSP00000398597 | EXOSC6 | 12 | 0.511 | 0.003643 | 0 |
| ENSP00000348551 | NCOR2 | 122 | 0.531 | 0.037037 | 919 |
| ENSP00000371432 | PRLR | 30 | 0.54 | 0.009107 | 268 |
| ENSP00000303242 | ITGB2 | 27 | 0.541 | 0.008197 | 340 |
| ENSP00000353483 | MAPK8 | 37 | 0.544 | 0.011233 | 858 |
| ENSP00000308450 | CDC20 | 24 | 0.545 | 0.007286 | 213 |
| ENSP00000367316 | ITGA8 | 27 | 0.547 | 0.008197 | 800 |
| ENSP00000351486 | NTRK1 | 80 | 0.557 | 0.024287 | 0 |
| ENSP00000296585 | ITGA2 | 288 | 0.56 | 0.087432 | 852 |
| ENSP00000350877 | SRSF2 | 2 | 0.566 | 0.000607 | 0 |
| ENSP00000304895 | IRS1 | 44 | 0.567 | 0.013358 | 859 |
| ENSP00000265023 | KNG1 | 24 | 0.597 | 0.007286 | 379 |
| ENSP00000264951 | XRN1 | 110 | 0.599 | 0.033394 | 221 |
| ENSP00000263967 | PIK3CA | 23 | 0.6 | 0.006982 | 500 |
| ENSP00000220592 | AGO2 | 121 | 0.6 | 0.036733 | 521 |
| ENSP00000308533 | GEMIN2 | 121 | 0.609 | 0.036733 | 229 |
| ENSP00000265171 | EGF | 4 | 0.612 | 0.001214 | 540 |
| ENSP00000358716 | DDX20 | 121 | 0.612 | 0.036733 | 278 |
| ENSP00000268712 | NCOR1 | 38 | 0.613 | 0.011536 | 989 |
| ENSP00000162330 | BCAR1 | 14 | 0.616 | 0.00425 | 229 |
| ENSP00000251849 | RAF1 | 105 | 0.628 | 0.031876 | 366 |
| ENSP00000300134 | STAT6 | 19 | 0.632 | 0.005768 | 900 |
| ENSP00000363868 | ABCA1 | 1 | 0.634 | 0.000304 | 990 |
| ENSP00000266970 | CDK2 | 70 | 0.635 | 0.021251 | 893 |
| ENSP00000414634 | LSM2 | 3 | 0.637 | 0.000911 | 0 |
| ENSP00000359345 | RPL5 | 27 | 0.651 | 0.008197 | 195 |
| ENSP00000313950 | AURKB | 27 | 0.654 | 0.008197 | 243 |
| ENSP00000338934 | EZR | 51 | 0.661 | 0.015483 | 430 |
| ENSP00000229022 | VDR | 86 | 0.674 | 0.026108 | 909 |
| ENSP00000264033 | CBL | 398 | 0.676 | 0.120826 | 249 |
| ENSP00000303830 | INSR | 54 | 0.677 | 0.016393 | 729 |
| ENSP00000335153 | HSP90AA1 | 91 | 0.677 | 0.027626 | 666 |
| ENSP00000339109 | ANAPC1 | 7 | 0.682 | 0.002125 | 0 |
| ENSP00000223095 | SERPINE1 | 10 | 0.69 | 0.003036 | 833 |
| ENSP00000302967 | HDAC3 | 4 | 0.693 | 0.001214 | 914 |
| ENSP00000262613 | SLC9A3R1 | 195 | 0.697 | 0.059199 | 892 |
| ENSP00000300413 | SNRPD1 | 121 | 0.71 | 0.036733 | 0 |
| ENSP00000300574 | CRK | 3 | 0.72 | 0.000911 | 307 |
| ENSP00000366135 | EXOSC10 | 132 | 0.726 | 0.040073 | 277 |
| ENSP00000252622 | LSM7 | 11 | 0.729 | 0.003339 | 0 |
| ENSP00000249299 | NAA38 | 2 | 0.731 | 0.000607 | 0 |
| ENSP00000342374 | SNRPD2 | 12 | 0.752 | 0.003643 | 0 |
| ENSP00000291552 | U2AF1 | 2 | 0.754 | 0.000607 | 0 |
| ENSP00000350941 | SRC | 180 | 0.759 | 0.054645 | 856 |
| ENSP00000240185 | TARDBP | 127 | 0.78 | 0.038555 | 177 |
| ENSP00000262320 | AXIN1 | 222 | 0.784 | 0.067395 | 902 |
| ENSP00000252102 | NDUFA2 | 122 | 0.795 | 0.037037 | 0 |
| ENSP00000337825 | LCK | 2 | 0.802 | 0.000607 | 380 |
| ENSP00000227378 | HSPA8 | 53 | 0.805 | 0.01609 | 619 |
| ENSP00000215829 | SNRPD3 | 11 | 0.807 | 0.003339 | 0 |
| ENSP00000361626 | YBX1 | 1 | 0.808 | 0.000304 | 860 |
| ENSP00000360683 | PTPN1 | 10 | 0.814 | 0.003036 | 347 |
| ENSP00000358997 | IRAK1 | 27 | 0.817 | 0.008197 | 278 |
| ENSP00000348554 | CDC16 | 1 | 0.83 | 0.000304 | 0 |
| ENSP00000370473 | IGFBP3 | 60 | 0.842 | 0.018215 | 729 |
| ENSP00000401303 | SHC1 | 76 | 0.844 | 0.023072 | 924 |
| ENSP00000384273 | RELA | 10 | 0.847 | 0.003036 | 800 |
| ENSP00000314491 | SRRT | 53 | 0.848 | 0.01609 | 155 |
| ENSP00000319169 | PRMT5 | 15 | 0.889 | 0.004554 | 0 |
| ENSP00000263309 | CLNS1A | 15 | 0.889 | 0.004554 | 0 |
| ENSP00000235090 | WDR77 | 15 | 0.913 | 0.004554 | 0 |
| ENSP00000378165 | ZNF207 | 15 | 0.915 | 0.004554 | 0 |
| ENSP00000357858 | BUB3 | 15 | 0.923 | 0.004554 | 0 |
| ENSP00000221494 | SF3A2 | 142 | 0.944 | 0.043109 | 0 |
| ENSP00000233946 | IL1R1 | 27 | 0.966 | 0.008197 | 240 |
| ENSP00000379625 | MYD88 | 27 | 0.969 | 0.008197 | 235 |
| ENSP00000307863 | U2AF2 | 137 | 0.969 | 0.041591 | 0 |
| ENSP00000313829 | KHDRBS1 | 84 | 0.983 | 0.025501 | 0 |

1. 514 candidate co-regeneration genes of bone and nerve

| **Ensembl ID** | **Gene symbol** | **Betweenness** | **Permutation FDR** | **Betweenness ratio** | **Min-Max interaction score** |
| --- | --- | --- | --- | --- | --- |
| ENSP00000384675 | SOS1 | 50 | <0.001 | 0.001458 | 928 |
| ENSP00000263354 | NAPA | 122 | 0.001 | 0.003559 | 185 |
| ENSP00000329384 | IL22 | 122 | 0.001 | 0.003559 | 540 |
| ENSP00000354895 | IFT140 | 1 | 0.001 | 2.92E-05 | 229 |
| ENSP00000321239 | RCHY1 | 122 | 0.002 | 0.003559 | 204 |
| ENSP00000345512 | SEMA6A | 1 | 0.003 | 2.92E-05 | 269 |
| ENSP00000376609 | GRK5 | 1 | 0.003 | 2.92E-05 | 919 |
| ENSP00000314444 | WDR35 | 1 | 0.006 | 2.92E-05 | 359 |
| ENSP00000162749 | TNFRSF1A | 208 | 0.009 | 0.006067 | 931 |
| ENSP00000358081 | BAG3 | 122 | 0.01 | 0.003559 | 800 |
| ENSP00000261267 | LYZ | 281 | 0.011 | 0.008197 | 951 |
| ENSP00000328511 | KCNA4 | 122 | 0.011 | 0.003559 | 208 |
| ENSP00000356000 | PLXNA2 | 1 | 0.011 | 2.92E-05 | 369 |
| ENSP00000302564 | BCL2L1 | 114 | 0.013 | 0.003325 | 892 |
| ENSP00000357711 | S100A7 | 122 | 0.013 | 0.003559 | 821 |
| ENSP00000323194 | PLXNA4 | 122 | 0.015 | 0.003559 | 386 |
| ENSP00000296695 | SPINK1 | 122 | 0.016 | 0.003559 | 271 |
| ENSP00000337014 | HFE2 | 30 | 0.016 | 0.000875 | 905 |
| ENSP00000355001 | POU3F3 | 122 | 0.016 | 0.003559 | 283 |
| ENSP00000279593 | GRIN2B | 122 | 0.017 | 0.003559 | 659 |
| ENSP00000317272 | MET | 802 | 0.019 | 0.023394 | 989 |
| ENSP00000342656 | EXT2 | 1 | 0.019 | 2.92E-05 | 926 |
| ENSP00000326227 | GANC | 1 | 0.02 | 2.92E-05 | 204 |
| ENSP00000035307 | CHPF2 | 276 | 0.021 | 0.008051 | 899 |
| ENSP00000325120 | PGR | 122 | 0.021 | 0.003559 | 923 |
| ENSP00000336762 | ANG | 122 | 0.023 | 0.003559 | 669 |
| ENSP00000308741 | CLOCK | 122 | 0.024 | 0.003559 | 919 |
| ENSP00000269593 | IGFBP4 | 281 | 0.025 | 0.008197 | 999 |
| ENSP00000283228 | PTPRR | 122 | 0.025 | 0.003559 | 257 |
| ENSP00000356346 | PTPRC | 81 | 0.025 | 0.002363 | 754 |
| ENSP00000419692 | RXRA | 459 | 0.026 | 0.013389 | 964 |
| ENSP00000370526 | ARSE | 3 | 0.028 | 8.75E-05 | 468 |
| ENSP00000287598 | BUB1B | 51 | 0.029 | 0.001488 | 227 |
| ENSP00000361658 | NUP188 | 281 | 0.029 | 0.008197 | 187 |
| ENSP00000354280 | PRSS3 | 122 | 0.031 | 0.003559 | 837 |
| ENSP00000368174 | MCM8 | 278 | 0.031 | 0.008109 | 653 |
| ENSP00000387286 | RAB1A | 122 | 0.031 | 0.003559 | 545 |
| ENSP00000266085 | TIMP3 | 122 | 0.033 | 0.003559 | 962 |
| ENSP00000263409 | LIFR | 122 | 0.036 | 0.003559 | 649 |
| ENSP00000376684 | EPHB6 | 122 | 0.038 | 0.003559 | 651 |
| ENSP00000250894 | MAPK8IP3 | 122 | 0.039 | 0.003559 | 0 |
| ENSP00000291442 | NR2F6 | 122 | 0.039 | 0.003559 | 926 |
| ENSP00000361066 | NCOA3 | 86 | 0.04 | 0.002509 | 999 |
| ENSP00000256078 | KRAS | 239 | 0.043 | 0.006972 | 941 |
| ENSP00000261207 | PPP1R12A | 122 | 0.043 | 0.003559 | 348 |
| ENSP00000316845 | ARHGEF4 | 122 | 0.043 | 0.003559 | 221 |
| ENSP00000204961 | EFNB1 | 122 | 0.044 | 0.003559 | 574 |
| ENSP00000227495 | ST3GAL4 | 1 | 0.044 | 2.92E-05 | 274 |
| ENSP00000228682 | GLI1 | 407 | 0.044 | 0.011872 | 978 |
| ENSP00000394794 | PTPN13 | 122 | 0.044 | 0.003559 | 224 |
| ENSP00000233813 | IGFBP5 | 281 | 0.046 | 0.008197 | 999 |
| ENSP00000367462 | OLAH | 122 | 0.046 | 0.003559 | 0 |
| ENSP00000350878 | S1PR3 | 263 | 0.047 | 0.007672 | 899 |
| ENSP00000331736 | SELE | 281 | 0.048 | 0.008197 | 970 |
| ENSP00000216124 | ARSA | 1 | 0.049 | 2.92E-05 | 899 |
| ENSP00000323065 | GADD45GIP1 | 122 | 0.05 | 0.003559 | 0 |
| ENSP00000311697 | FGF5 | 281 | 0.051 | 0.008197 | 966 |
| ENSP00000358617 | PHTF1 | 1 | 0.051 | 2.92E-05 | 0 |
| ENSP00000406878 | PSMB8 | 122 | 0.051 | 0.003559 | 190 |
| ENSP00000314508 | GBA | 1 | 0.052 | 2.92E-05 | 515 |
| ENSP00000253925 | PPFIA1 | 122 | 0.053 | 0.003559 | 175 |
| ENSP00000351605 | FZD6 | 281 | 0.054 | 0.008197 | 899 |
| ENSP00000356918 | STX7 | 122 | 0.054 | 0.003559 | 236 |
| ENSP00000261733 | ALDH2 | 2 | 0.057 | 5.83E-05 | 290 |
| ENSP00000329357 | SP1 | 23 | 0.057 | 0.000671 | 988 |
| ENSP00000316244 | HTR1A | 275 | 0.058 | 0.008022 | 960 |
| ENSP00000333001 | RBM8A | 2 | 0.058 | 5.83E-05 | 270 |
| ENSP00000252945 | CYP2E1 | 7 | 0.06 | 0.000204 | 912 |
| ENSP00000374357 | ARNTL | 122 | 0.061 | 0.003559 | 919 |
| ENSP00000275874 | RAB19 | 122 | 0.064 | 0.003559 | 204 |
| ENSP00000320758 | NOS1 | 244 | 0.064 | 0.007117 | 682 |
| ENSP00000417864 | ANP32A | 122 | 0.064 | 0.003559 | 0 |
| ENSP00000266987 | TARBP2 | 1 | 0.066 | 2.92E-05 | 424 |
| ENSP00000296181 | ITGB5 | 6 | 0.066 | 0.000175 | 809 |
| ENSP00000354560 | KIFAP3 | 5 | 0.066 | 0.000146 | 245 |
| ENSP00000200453 | PPP1R15A | 105 | 0.067 | 0.003063 | 899 |
| ENSP00000263864 | VAMP8 | 122 | 0.067 | 0.003559 | 249 |
| ENSP00000251772 | PLXNA1 | 4 | 0.068 | 0.000117 | 946 |
| ENSP00000258962 | SRSF1 | 62 | 0.068 | 0.001809 | 251 |
| ENSP00000299855 | MMP3 | 280 | 0.068 | 0.008168 | 933 |
| ENSP00000347169 | NUMB | 3 | 0.068 | 8.75E-05 | 333 |
| ENSP00000362994 | TRAF1 | 122 | 0.069 | 0.003559 | 427 |
| ENSP00000008527 | CRY1 | 122 | 0.07 | 0.003559 | 535 |
| ENSP00000264554 | SHC2 | 562 | 0.07 | 0.016393 | 993 |
| ENSP00000371973 | SAP18 | 4 | 0.071 | 0.000117 | 899 |
| ENSP00000254657 | PER2 | 122 | 0.072 | 0.003559 | 574 |
| ENSP00000374372 | SPTB | 120 | 0.072 | 0.0035 | 340 |
| ENSP00000342392 | MESP2 | 561 | 0.073 | 0.016364 | 613 |
| ENSP00000264426 | GRIA2 | 5 | 0.078 | 0.000146 | 432 |
| ENSP00000003084 | CFTR | 2052 | 0.079 | 0.059856 | 897 |
| ENSP00000164227 | BCL3 | 281 | 0.081 | 0.008197 | 630 |
| ENSP00000305603 | FUT3 | 1 | 0.083 | 2.92E-05 | 346 |
| ENSP00000314520 | KCNA2 | 122 | 0.084 | 0.003559 | 891 |
| ENSP00000357753 | IVL | 122 | 0.086 | 0.003559 | 308 |
| ENSP00000393725 | GFRA1 | 364 | 0.086 | 0.010618 | 813 |
| ENSP00000256857 | GRP | 15 | 0.088 | 0.000438 | 919 |
| ENSP00000376177 | CALCRL | 122 | 0.089 | 0.003559 | 977 |
| ENSP00000260605 | DYNC2LI1 | 280 | 0.09 | 0.008168 | 166 |
| ENSP00000250003 | MYOD1 | 122 | 0.092 | 0.003559 | 970 |
| ENSP00000282091 | PTH | 7 | 0.093 | 0.000204 | 962 |
| ENSP00000247668 | TRAF2 | 716 | 0.095 | 0.020886 | 768 |
| ENSP00000246032 | STK35 | 122 | 0.099 | 0.003559 | 353 |
| ENSP00000261464 | TRAF5 | 122 | 0.099 | 0.003559 | 241 |
| ENSP00000375921 | PAX3 | 244 | 0.1 | 0.007117 | 833 |
| ENSP00000293549 | WNT1 | 280 | 0.102 | 0.008168 | 960 |
| ENSP00000349437 | IGF2R | 27 | 0.102 | 0.000788 | 609 |
| ENSP00000357292 | UBQLN4 | 1 | 0.102 | 2.92E-05 | 812 |
| ENSP00000363822 | AR | 47 | 0.103 | 0.001371 | 902 |
| ENSP00000299106 | JAM3 | 122 | 0.104 | 0.003559 | 200 |
| ENSP00000364864 | KIF3B | 4 | 0.104 | 0.000117 | 340 |
| ENSP00000391069 | SRPK1 | 60 | 0.104 | 0.00175 | 184 |
| ENSP00000209668 | ADH1A | 2 | 0.107 | 5.83E-05 | 308 |
| ENSP00000297785 | ALDH1A1 | 2 | 0.107 | 5.83E-05 | 829 |
| ENSP00000368020 | KIF3A | 117 | 0.109 | 0.003413 | 800 |
| ENSP00000311113 | JUP | 1 | 0.11 | 2.92E-05 | 977 |
| ENSP00000249647 | SNAP23 | 122 | 0.111 | 0.003559 | 305 |
| ENSP00000227752 | IL10RA | 122 | 0.113 | 0.003559 | 469 |
| ENSP00000287641 | SST | 2 | 0.113 | 5.83E-05 | 970 |
| ENSP00000357927 | BNIPL | 37 | 0.113 | 0.001079 | 619 |
| ENSP00000044462 | PSMA4 | 121 | 0.114 | 0.00353 | 307 |
| ENSP00000309968 | ADAM17 | 119 | 0.114 | 0.003471 | 921 |
| ENSP00000354130 | SOX10 | 244 | 0.114 | 0.007117 | 911 |
| ENSP00000224237 | VIM | 122 | 0.115 | 0.003559 | 543 |
| ENSP00000283195 | RANBP2 | 116 | 0.115 | 0.003384 | 525 |
| ENSP00000315644 | TYMS | 122 | 0.115 | 0.003559 | 480 |
| ENSP00000308533 | GEMIN2 | 120 | 0.116 | 0.0035 | 265 |
| ENSP00000358716 | DDX20 | 120 | 0.116 | 0.0035 | 278 |
| ENSP00000412237 | IL10 | 122 | 0.116 | 0.003559 | 969 |
| ENSP00000290200 | IL10RB | 122 | 0.117 | 0.003559 | 212 |
| ENSP00000257904 | CDK4 | 22 | 0.118 | 0.000642 | 839 |
| ENSP00000278385 | CD44 | 470 | 0.119 | 0.01371 | 966 |
| ENSP00000360525 | MAGOH | 4 | 0.121 | 0.000117 | 0 |
| ENSP00000357656 | FYN | 950 | 0.123 | 0.027711 | 984 |
| ENSP00000339992 | MYB | 281 | 0.124 | 0.008197 | 974 |
| ENSP00000375986 | MAP3K4 | 122 | 0.126 | 0.003559 | 619 |
| ENSP00000343204 | JAK1 | 248 | 0.127 | 0.007234 | 963 |
| ENSP00000223642 | C5 | 1 | 0.128 | 2.92E-05 | 899 |
| ENSP00000352929 | CSNK1E | 122 | 0.128 | 0.003559 | 956 |
| ENSP00000219548 | STUB1 | 301 | 0.129 | 0.00878 | 621 |
| ENSP00000238081 | YWHAQ | 1 | 0.13 | 2.92E-05 | 866 |
| ENSP00000381066 | MAP2K7 | 122 | 0.13 | 0.003559 | 802 |
| ENSP00000265333 | VDAC1 | 112 | 0.131 | 0.003267 | 229 |
| ENSP00000222812 | STX1A | 609 | 0.134 | 0.017764 | 287 |
| ENSP00000226730 | IL2 | 28 | 0.134 | 0.000817 | 990 |
| ENSP00000308461 | RND1 | 2 | 0.134 | 5.83E-05 | 265 |
| ENSP00000310491 | ARHGAP1 | 37 | 0.135 | 0.001079 | 229 |
| ENSP00000259633 | CD72 | 40 | 0.136 | 0.001167 | 208 |
| ENSP00000316054 | DVL3 | 281 | 0.136 | 0.008197 | 969 |
| ENSP00000369050 | CYP1A1 | 552 | 0.14 | 0.016102 | 907 |
| ENSP00000310596 | LSM1 | 7 | 0.143 | 0.000204 | 210 |
| ENSP00000327647 | CRADD | 122 | 0.143 | 0.003559 | 196 |
| ENSP00000396127 | RAN | 616 | 0.143 | 0.017969 | 374 |
| ENSP00000264914 | ARSB | 2 | 0.145 | 5.83E-05 | 287 |
| ENSP00000245544 | NUP85 | 122 | 0.146 | 0.003559 | 0 |
| ENSP00000363071 | DES | 400 | 0.148 | 0.011668 | 466 |
| ENSP00000386259 | NEB | 400 | 0.148 | 0.011668 | 463 |
| ENSP00000296440 | PLXNB1 | 1 | 0.149 | 2.92E-05 | 405 |
| ENSP00000227667 | APOC3 | 123 | 0.15 | 0.003588 | 998 |
| ENSP00000338799 | IL6ST | 621 | 0.152 | 0.018114 | 999 |
| ENSP00000276420 | DOK2 | 242 | 0.153 | 0.007059 | 906 |
| ENSP00000236671 | CTSD | 121 | 0.154 | 0.00353 | 412 |
| ENSP00000271688 | CERS2 | 280 | 0.154 | 0.008168 | 899 |
| ENSP00000327583 | RANBP1 | 1 | 0.154 | 2.92E-05 | 176 |
| ENSP00000162330 | BCAR1 | 299 | 0.155 | 0.008722 | 925 |
| ENSP00000348577 | RANGAP1 | 620 | 0.156 | 0.018085 | 263 |
| ENSP00000294172 | NXF1 | 523 | 0.157 | 0.015256 | 307 |
| ENSP00000216225 | RBX1 | 341 | 0.161 | 0.009947 | 959 |
| ENSP00000340820 | MAPT | 241 | 0.161 | 0.00703 | 998 |
| ENSP00000391901 | PHF1 | 79 | 0.163 | 0.002304 | 329 |
| ENSP00000355537 | ACTN2 | 122 | 0.165 | 0.003559 | 229 |
| ENSP00000262477 | RABEP1 | 103 | 0.166 | 0.003004 | 362 |
| ENSP00000305692 | GAA | 126 | 0.166 | 0.003675 | 430 |
| ENSP00000372793 | LTA | 208 | 0.167 | 0.006067 | 647 |
| ENSP00000233946 | IL1R1 | 397 | 0.169 | 0.01158 | 999 |
| ENSP00000257430 | APC | 122 | 0.169 | 0.003559 | 991 |
| ENSP00000305416 | S1PR1 | 6 | 0.169 | 0.000175 | 985 |
| ENSP00000337103 | CHAT | 281 | 0.169 | 0.008197 | 955 |
| ENSP00000408910 | DCTN2 | 1 | 0.169 | 2.92E-05 | 587 |
| ENSP00000362082 | CCND3 | 244 | 0.171 | 0.007117 | 829 |
| ENSP00000284957 | RABGEF1 | 99 | 0.173 | 0.002888 | 281 |
| ENSP00000295731 | IHH | 488 | 0.173 | 0.014235 | 838 |
| ENSP00000292408 | FGFR4 | 281 | 0.174 | 0.008197 | 978 |
| ENSP00000299293 | FRS2 | 16 | 0.174 | 0.000467 | 983 |
| ENSP00000241014 | MAPK8IP1 | 122 | 0.178 | 0.003559 | 324 |
| ENSP00000360286 | RAE1 | 402 | 0.178 | 0.011726 | 358 |
| ENSP00000230449 | EXOC2 | 1 | 0.179 | 2.92E-05 | 0 |
| ENSP00000337224 | LRAT | 8 | 0.179 | 0.000233 | 534 |
| ENSP00000242480 | EGR2 | 244 | 0.18 | 0.007117 | 817 |
| ENSP00000355651 | RAB4A | 4 | 0.182 | 0.000117 | 0 |
| ENSP00000387662 | GCG | 254 | 0.183 | 0.007409 | 984 |
| ENSP00000351407 | ARNT | 535 | 0.184 | 0.015606 | 984 |
| ENSP00000189444 | NFKB2 | 202 | 0.185 | 0.005892 | 940 |
| ENSP00000233057 | EIF2AK2 | 1 | 0.185 | 2.92E-05 | 170 |
| ENSP00000303325 | TACR3 | 3 | 0.186 | 8.75E-05 | 948 |
| ENSP00000326550 | TACC3 | 2 | 0.186 | 5.83E-05 | 502 |
| ENSP00000340944 | PTPN11 | 808 | 0.187 | 0.023569 | 993 |
| ENSP00000222725 | LFNG | 561 | 0.188 | 0.016364 | 860 |
| ENSP00000262238 | YY1 | 178 | 0.188 | 0.005192 | 943 |
| ENSP00000294724 | AGL | 1 | 0.188 | 2.92E-05 | 257 |
| ENSP00000352400 | NUP214 | 507 | 0.191 | 0.014789 | 219 |
| ENSP00000332353 | PTCH1 | 1912 | 0.195 | 0.055773 | 998 |
| ENSP00000331358 | GAST | 8 | 0.2 | 0.000233 | 939 |
| ENSP00000345571 | E2F1 | 15 | 0.2 | 0.000438 | 986 |
| ENSP00000252997 | GATA5 | 122 | 0.201 | 0.003559 | 816 |
| ENSP00000005257 | RALA | 1 | 0.202 | 2.92E-05 | 348 |
| ENSP00000296785 | ANKRA2 | 2 | 0.204 | 5.83E-05 | 207 |
| ENSP00000304669 | CTNNA1 | 122 | 0.204 | 0.003559 | 906 |
| ENSP00000342793 | PLD1 | 1 | 0.205 | 2.92E-05 | 826 |
| ENSP00000216223 | IL2RB | 117 | 0.206 | 0.003413 | 424 |
| ENSP00000267814 | SORD | 112 | 0.206 | 0.003267 | 221 |
| ENSP00000369442 | KL | 150 | 0.206 | 0.004375 | 611 |
| ENSP00000332468 | TRAF3 | 121 | 0.207 | 0.00353 | 866 |
| ENSP00000359345 | RPL5 | 281 | 0.208 | 0.008197 | 373 |
| ENSP00000289779 | F11R | 1 | 0.209 | 2.92E-05 | 345 |
| ENSP00000170630 | IL4R | 184 | 0.21 | 0.005367 | 906 |
| ENSP00000289902 | FCER1G | 244 | 0.21 | 0.007117 | 389 |
| ENSP00000348708 | UPF2 | 17 | 0.212 | 0.000496 | 228 |
| ENSP00000362649 | HDAC1 | 405 | 0.212 | 0.011814 | 967 |
| ENSP00000349465 | PICK1 | 5 | 0.213 | 0.000146 | 283 |
| ENSP00000306920 | GLB1 | 152 | 0.214 | 0.004434 | 904 |
| ENSP00000355261 | SMG5 | 8 | 0.214 | 0.000233 | 276 |
| ENSP00000225831 | CCL2 | 10 | 0.215 | 0.000292 | 976 |
| ENSP00000368438 | PCNA | 773 | 0.215 | 0.022548 | 900 |
| ENSP00000229307 | NANOG | 281 | 0.219 | 0.008197 | 877 |
| ENSP00000268058 | PML | 22 | 0.222 | 0.000642 | 964 |
| ENSP00000338934 | EZR | 1239 | 0.222 | 0.036141 | 874 |
| ENSP00000348551 | NCOR2 | 10 | 0.222 | 0.000292 | 919 |
| ENSP00000308208 | MMP14 | 278 | 0.224 | 0.008109 | 978 |
| ENSP00000262340 | RPE65 | 8 | 0.225 | 0.000233 | 542 |
| ENSP00000350877 | SRSF2 | 6 | 0.228 | 0.000175 | 243 |
| ENSP00000354394 | STAT1 | 319 | 0.228 | 0.009305 | 994 |
| ENSP00000296581 | LSM6 | 1 | 0.23 | 2.92E-05 | 0 |
| ENSP00000351273 | CASP8 | 122 | 0.232 | 0.003559 | 670 |
| ENSP00000373614 | SELPLG | 281 | 0.234 | 0.008197 | 649 |
| ENSP00000302728 | GUSB | 150 | 0.235 | 0.004375 | 317 |
| ENSP00000261908 | NEO1 | 142 | 0.236 | 0.004142 | 297 |
| ENSP00000366135 | EXOSC10 | 276 | 0.237 | 0.008051 | 270 |
| ENSP00000282588 | ITGA1 | 122 | 0.238 | 0.003559 | 918 |
| ENSP00000352516 | DNMT1 | 5 | 0.239 | 0.000146 | 844 |
| ENSP00000248244 | TICAM1 | 122 | 0.24 | 0.003559 | 208 |
| ENSP00000360519 | RBP4 | 8 | 0.24 | 0.000233 | 661 |
| ENSP00000265709 | ANK1 | 120 | 0.242 | 0.0035 | 374 |
| ENSP00000320180 | GHRHR | 1 | 0.247 | 2.92E-05 | 903 |
| ENSP00000311430 | RPL4 | 281 | 0.248 | 0.008197 | 527 |
| ENSP00000344115 | CDH5 | 46 | 0.248 | 0.001342 | 998 |
| ENSP00000262613 | SLC9A3R1 | 1477 | 0.249 | 0.043084 | 957 |
| ENSP00000320147 | EZH2 | 79 | 0.249 | 0.002304 | 943 |
| ENSP00000363763 | EPHB2 | 38 | 0.25 | 0.001108 | 729 |
| ENSP00000220592 | AGO2 | 120 | 0.254 | 0.0035 | 521 |
| ENSP00000226574 | NFKB1 | 79 | 0.254 | 0.002304 | 988 |
| ENSP00000302961 | HSPA4 | 150 | 0.254 | 0.004375 | 993 |
| ENSP00000306245 | FOS | 1350 | 0.257 | 0.039379 | 994 |
| ENSP00000261769 | CDH1 | 10 | 0.258 | 0.000292 | 927 |
| ENSP00000318585 | BACE1 | 122 | 0.26 | 0.003559 | 825 |
| ENSP00000330393 | LEPR | 131 | 0.26 | 0.003821 | 998 |
| ENSP00000228837 | FGF6 | 837 | 0.261 | 0.024415 | 999 |
| ENSP00000357392 | EFNA1 | 399 | 0.262 | 0.011639 | 999 |
| ENSP00000284981 | APP | 122 | 0.263 | 0.003559 | 968 |
| ENSP00000341344 | GGA1 | 126 | 0.265 | 0.003675 | 196 |
| ENSP00000376076 | SUMO1 | 42 | 0.266 | 0.001225 | 943 |
| ENSP00000351908 | MAP3K5 | 280 | 0.267 | 0.008168 | 306 |
| ENSP00000218388 | TIMP1 | 292 | 0.269 | 0.008518 | 996 |
| ENSP00000327850 | NFATC1 | 244 | 0.269 | 0.007117 | 922 |
| ENSP00000309103 | BAD | 6 | 0.27 | 0.000175 | 919 |
| ENSP00000411698 | USO1 | 122 | 0.271 | 0.003559 | 161 |
| ENSP00000243776 | CHPF | 4 | 0.274 | 0.000117 | 922 |
| ENSP00000264926 | RAD18 | 122 | 0.276 | 0.003559 | 495 |
| ENSP00000414634 | LSM2 | 13 | 0.276 | 0.000379 | 0 |
| ENSP00000263033 | SYTL4 | 121 | 0.277 | 0.00353 | 0 |
| ENSP00000262803 | UPF1 | 8 | 0.28 | 0.000233 | 232 |
| ENSP00000206249 | ESR1 | 3076 | 0.284 | 0.089726 | 994 |
| ENSP00000334122 | FGF3 | 281 | 0.285 | 0.008197 | 999 |
| ENSP00000342374 | SNRPD2 | 13 | 0.287 | 0.000379 | 241 |
| ENSP00000301838 | FADD | 2 | 0.293 | 5.83E-05 | 379 |
| ENSP00000281537 | TJP1 | 1 | 0.295 | 2.92E-05 | 908 |
| ENSP00000309845 | HRAS | 330 | 0.297 | 0.009626 | 989 |
| ENSP00000413035 | RBFOX2 | 2 | 0.297 | 5.83E-05 | 317 |
| ENSP00000337761 | RAB27A | 121 | 0.298 | 0.00353 | 824 |
| ENSP00000348786 | RAP1A | 122 | 0.299 | 0.003559 | 340 |
| ENSP00000398597 | EXOSC6 | 4 | 0.3 | 0.000117 | 0 |
| ENSP00000262629 | TYROBP | 29 | 0.302 | 0.000846 | 630 |
| ENSP00000361850 | PLAU | 149 | 0.303 | 0.004346 | 974 |
| ENSP00000259371 | DAB2IP | 280 | 0.304 | 0.008168 | 483 |
| ENSP00000269349 | EIF4A3 | 2 | 0.304 | 5.83E-05 | 0 |
| ENSP00000262768 | TIMP2 | 278 | 0.307 | 0.008109 | 999 |
| ENSP00000300413 | SNRPD1 | 120 | 0.307 | 0.0035 | 326 |
| ENSP00000291700 | S100B | 122 | 0.308 | 0.003559 | 844 |
| ENSP00000416097 | GOLGA2 | 122 | 0.311 | 0.003559 | 184 |
| ENSP00000356713 | IFNGR1 | 122 | 0.313 | 0.003559 | 413 |
| ENSP00000227507 | CCND1 | 1726 | 0.314 | 0.050347 | 992 |
| ENSP00000361818 | SDC4 | 244 | 0.316 | 0.007117 | 918 |
| ENSP00000378529 | FZR1 | 2 | 0.317 | 5.83E-05 | 0 |
| ENSP00000240185 | TARDBP | 250 | 0.321 | 0.007292 | 177 |
| ENSP00000261205 | SYT1 | 121 | 0.321 | 0.00353 | 621 |
| ENSP00000385269 | ELAVL1 | 2 | 0.321 | 5.83E-05 | 975 |
| ENSP00000299402 | APBB1 | 25 | 0.322 | 0.000729 | 419 |
| ENSP00000254066 | RARA | 30 | 0.323 | 0.000875 | 902 |
| ENSP00000338297 | IGF2 | 27 | 0.326 | 0.000788 | 966 |
| ENSP00000417404 | HFE | 32 | 0.329 | 0.000933 | 425 |
| ENSP00000320940 | NCOA1 | 1293 | 0.331 | 0.037717 | 995 |
| ENSP00000324897 | UBE2I | 753 | 0.331 | 0.021965 | 876 |
| ENSP00000316879 | EIF4G1 | 2 | 0.332 | 5.83E-05 | 374 |
| ENSP00000332643 | NDN | 15 | 0.332 | 0.000438 | 934 |
| ENSP00000300935 | RAB8A | 1 | 0.333 | 2.92E-05 | 204 |
| ENSP00000303522 | TACR1 | 1 | 0.336 | 2.92E-05 | 905 |
| ENSP00000291552 | U2AF1 | 5 | 0.338 | 0.000146 | 316 |
| ENSP00000350512 | COPS5 | 122 | 0.338 | 0.003559 | 987 |
| ENSP00000269485 | TNFRSF11A | 122 | 0.339 | 0.003559 | 813 |
| ENSP00000302150 | PRL | 699 | 0.339 | 0.02039 | 991 |
| ENSP00000264708 | POMC | 9 | 0.346 | 0.000263 | 973 |
| ENSP00000284384 | PRKCA | 128 | 0.346 | 0.003734 | 848 |
| ENSP00000360025 | GADD45A | 244 | 0.346 | 0.007117 | 946 |
| ENSP00000343040 | HMGB1 | 244 | 0.348 | 0.007117 | 540 |
| ENSP00000276414 | GNRH1 | 119 | 0.351 | 0.003471 | 953 |
| ENSP00000260762 | EXOC6 | 1 | 0.353 | 2.92E-05 | 263 |
| ENSP00000344468 | SDC3 | 400 | 0.355 | 0.011668 | 985 |
| ENSP00000300651 | MED1 | 57 | 0.364 | 0.001663 | 462 |
| ENSP00000301019 | CDT1 | 3 | 0.364 | 8.75E-05 | 271 |
| ENSP00000209728 | CDC6 | 308 | 0.366 | 0.008984 | 581 |
| ENSP00000269260 | ARRB2 | 122 | 0.368 | 0.003559 | 428 |
| ENSP00000229022 | VDR | 758 | 0.372 | 0.022111 | 978 |
| ENSP00000377141 | ARRB1 | 121 | 0.373 | 0.00353 | 769 |
| ENSP00000407431 | HLA-C | 32 | 0.375 | 0.000933 | 241 |
| ENSP00000278568 | PAK1 | 1 | 0.376 | 2.92E-05 | 857 |
| ENSP00000315859 | RNPS1 | 10 | 0.38 | 0.000292 | 0 |
| ENSP00000227378 | HSPA8 | 177 | 0.382 | 0.005163 | 619 |
| ENSP00000302955 | RRM2 | 10 | 0.389 | 0.000292 | 824 |
| ENSP00000316042 | HNRNPA0 | 48 | 0.389 | 0.0014 | 187 |
| ENSP00000313752 | SSNA1 | 122 | 0.39 | 0.003559 | 0 |
| ENSP00000340858 | B2M | 32 | 0.391 | 0.000933 | 929 |
| ENSP00000288986 | NCK1 | 1 | 0.394 | 2.92E-05 | 983 |
| ENSP00000216911 | AURKA | 124 | 0.395 | 0.003617 | 965 |
| ENSP00000389140 | DCC | 330 | 0.395 | 0.009626 | 608 |
| ENSP00000317714 | STX4 | 122 | 0.398 | 0.003559 | 256 |
| ENSP00000052754 | DCN | 476 | 0.399 | 0.013885 | 945 |
| ENSP00000308450 | CDC20 | 446 | 0.4 | 0.01301 | 621 |
| ENSP00000353224 | TFRC | 32 | 0.403 | 0.000933 | 927 |
| ENSP00000355325 | PSMB5 | 121 | 0.403 | 0.00353 | 190 |
| ENSP00000262643 | CCNE1 | 25 | 0.404 | 0.000729 | 861 |
| ENSP00000249299 | NAA38 | 2 | 0.407 | 5.83E-05 | 0 |
| ENSP00000237837 | FGF23 | 712 | 0.408 | 0.020769 | 988 |
| ENSP00000287727 | ZFYVE9 | 115 | 0.408 | 0.003355 | 424 |
| ENSP00000365380 | FOXP3 | 488 | 0.408 | 0.014235 | 948 |
| ENSP00000252622 | LSM7 | 10 | 0.414 | 0.000292 | 204 |
| ENSP00000296871 | CSF2 | 50 | 0.414 | 0.001458 | 972 |
| ENSP00000311032 | CASP3 | 2 | 0.414 | 5.83E-05 | 991 |
| ENSP00000389934 | EXOC5 | 1 | 0.415 | 2.92E-05 | 0 |
| ENSP00000344818 | UBC | 17611 | 0.42 | 0.51371 | 999 |
| ENSP00000256383 | EIF2S1 | 1 | 0.421 | 2.92E-05 | 913 |
| ENSP00000354586 | GLI2 | 15 | 0.429 | 0.000438 | 997 |
| ENSP00000384053 | CSF2RB | 50 | 0.431 | 0.001458 | 621 |
| ENSP00000362820 | SRSF3 | 6 | 0.433 | 0.000175 | 270 |
| ENSP00000262435 | SMURF2 | 25 | 0.434 | 0.000729 | 933 |
| ENSP00000222254 | PIK3R2 | 2 | 0.435 | 5.83E-05 | 904 |
| ENSP00000335153 | HSP90AA1 | 810 | 0.436 | 0.023628 | 999 |
| ENSP00000366013 | GNB2L1 | 30 | 0.437 | 0.000875 | 997 |
| ENSP00000267163 | RB1 | 127 | 0.438 | 0.003705 | 972 |
| ENSP00000237527 | GHRH | 1 | 0.439 | 2.92E-05 | 951 |
| ENSP00000371067 | JAK2 | 1312 | 0.439 | 0.038271 | 994 |
| ENSP00000363921 | PARD3 | 118 | 0.441 | 0.003442 | 462 |
| ENSP00000260130 | SDCBP | 121 | 0.443 | 0.00353 | 540 |
| ENSP00000262633 | RBM42 | 2 | 0.444 | 5.83E-05 | 200 |
| ENSP00000300161 | YWHAB | 130 | 0.444 | 0.003792 | 905 |
| ENSP00000341551 | SMAD4 | 1445 | 0.444 | 0.04215 | 999 |
| ENSP00000313950 | AURKB | 400 | 0.449 | 0.011668 | 404 |
| ENSP00000347198 | SRGAP1 | 487 | 0.449 | 0.014206 | 246 |
| ENSP00000274026 | CCNA2 | 6 | 0.45 | 0.000175 | 426 |
| ENSP00000222256 | RAB3A | 121 | 0.451 | 0.00353 | 318 |
| ENSP00000242152 | NPY | 121 | 0.451 | 0.00353 | 980 |
| ENSP00000266000 | DAXX | 5 | 0.454 | 0.000146 | 621 |
| ENSP00000354791 | DCTN1 | 280 | 0.455 | 0.008168 | 620 |
| ENSP00000350941 | SRC | 2574 | 0.456 | 0.075083 | 995 |
| ENSP00000329967 | TBK1 | 122 | 0.459 | 0.003559 | 379 |
| ENSP00000382004 | CTNND1 | 20 | 0.46 | 0.000583 | 936 |
| ENSP00000361418 | IPO13 | 61 | 0.461 | 0.001779 | 200 |
| ENSP00000351665 | CLIP1 | 279 | 0.463 | 0.008138 | 412 |
| ENSP00000332973 | SMAD3 | 1719 | 0.468 | 0.050143 | 999 |
| ENSP00000418447 | PPP2CA | 133 | 0.472 | 0.00388 | 863 |
| ENSP00000284811 | TCEB1 | 30 | 0.479 | 0.000875 | 978 |
| ENSP00000391592 | PTPN6 | 45 | 0.479 | 0.001313 | 996 |
| ENSP00000370343 | IRF4 | 4 | 0.48 | 0.000117 | 814 |
| ENSP00000400591 | SNRPE | 4 | 0.48 | 0.000117 | 0 |
| ENSP00000371432 | PRLR | 689 | 0.481 | 0.020098 | 542 |
| ENSP00000221494 | SF3A2 | 517 | 0.486 | 0.015081 | 944 |
| ENSP00000278616 | ATM | 13 | 0.495 | 0.000379 | 800 |
| ENSP00000280892 | EIF4E | 2 | 0.496 | 5.83E-05 | 430 |
| ENSP00000351486 | NTRK1 | 2226 | 0.499 | 0.064932 | 999 |
| ENSP00000370571 | TH | 281 | 0.512 | 0.008197 | 947 |
| ENSP00000363998 | ITCH | 242 | 0.518 | 0.007059 | 800 |
| ENSP00000350616 | DDC | 281 | 0.521 | 0.008197 | 379 |
| ENSP00000307046 | SDC2 | 375 | 0.522 | 0.010939 | 939 |
| ENSP00000232014 | BCL6 | 4 | 0.523 | 0.000117 | 871 |
| ENSP00000359988 | SRSF11 | 6 | 0.523 | 0.000175 | 283 |
| ENSP00000303706 | CDC25A | 5 | 0.524 | 0.000146 | 303 |
| ENSP00000262320 | AXIN1 | 1480 | 0.527 | 0.043171 | 999 |
| ENSP00000367207 | MYC | 150 | 0.531 | 0.004375 | 998 |
| ENSP00000358541 | SIKE1 | 9 | 0.532 | 0.000263 | 0 |
| ENSP00000358022 | MCL1 | 225 | 0.533 | 0.006563 | 906 |
| ENSP00000263753 | SGOL1 | 116 | 0.537 | 0.003384 | 0 |
| ENSP00000315955 | FOXA2 | 130 | 0.538 | 0.003792 | 937 |
| ENSP00000351163 | COL11A1 | 2 | 0.542 | 5.83E-05 | 900 |
| ENSP00000364000 | COL5A2 | 281 | 0.547 | 0.008197 | 914 |
| ENSP00000401303 | SHC1 | 2141 | 0.548 | 0.062453 | 994 |
| ENSP00000356087 | IKBKE | 9 | 0.549 | 0.000263 | 260 |
| ENSP00000362900 | SRSF4 | 1 | 0.549 | 2.92E-05 | 0 |
| ENSP00000335544 | CCKBR | 8 | 0.55 | 0.000233 | 909 |
| ENSP00000379625 | MYD88 | 518 | 0.551 | 0.01511 | 990 |
| ENSP00000335657 | CCK | 8 | 0.552 | 0.000233 | 967 |
| ENSP00000342215 | KIR2DL3 | 32 | 0.554 | 0.000933 | 227 |
| ENSP00000281708 | FBXW7 | 26 | 0.555 | 0.000758 | 899 |
| ENSP00000302530 | BUB1 | 25 | 0.555 | 0.000729 | 228 |
| ENSP00000263967 | PIK3CA | 96 | 0.557 | 0.0028 | 972 |
| ENSP00000350003 | CCR3 | 112 | 0.558 | 0.003267 | 899 |
| ENSP00000352980 | HIST1H4A | 13 | 0.562 | 0.000379 | 901 |
| ENSP00000358997 | IRAK1 | 992 | 0.562 | 0.028936 | 938 |
| ENSP00000256474 | VHL | 3009 | 0.566 | 0.087772 | 998 |
| ENSP00000271628 | SF3B4 | 120 | 0.566 | 0.0035 | 346 |
| ENSP00000215829 | SNRPD3 | 41 | 0.567 | 0.001196 | 269 |
| ENSP00000338983 | MUC1 | 122 | 0.575 | 0.003559 | 867 |
| ENSP00000276201 | UPF3B | 17 | 0.58 | 0.000496 | 296 |
| ENSP00000219476 | TSC2 | 70 | 0.583 | 0.002042 | 998 |
| ENSP00000337825 | LCK | 207 | 0.587 | 0.006038 | 913 |
| ENSP00000227758 | BIRC2 | 122 | 0.589 | 0.003559 | 353 |
| ENSP00000314491 | SRRT | 177 | 0.597 | 0.005163 | 155 |
| ENSP00000263686 | SELP | 402 | 0.602 | 0.011726 | 906 |
| ENSP00000340330 | KAT5 | 25 | 0.603 | 0.000729 | 992 |
| ENSP00000292644 | PSMC2 | 121 | 0.61 | 0.00353 | 438 |
| ENSP00000265023 | KNG1 | 262 | 0.613 | 0.007642 | 953 |
| ENSP00000231509 | NR3C1 | 35 | 0.617 | 0.001021 | 917 |
| ENSP00000307863 | U2AF2 | 461 | 0.617 | 0.013447 | 367 |
| ENSP00000219255 | PARD6A | 117 | 0.618 | 0.003413 | 946 |
| ENSP00000339007 | GRB2 | 2320 | 0.618 | 0.067674 | 990 |
| ENSP00000256452 | IL5RA | 121 | 0.62 | 0.00353 | 375 |
| ENSP00000361423 | ABL1 | 13 | 0.621 | 0.000379 | 832 |
| ENSP00000297518 | CDK5 | 241 | 0.622 | 0.00703 | 646 |
| ENSP00000379330 | NFATC2 | 48 | 0.626 | 0.0014 | 917 |
| ENSP00000296585 | ITGA2 | 558 | 0.627 | 0.016277 | 852 |
| ENSP00000309503 | YWHAZ | 4 | 0.628 | 0.000117 | 784 |
| ENSP00000256442 | CCNB1 | 3069 | 0.636 | 0.089522 | 925 |
| ENSP00000300574 | CRK | 330 | 0.647 | 0.009626 | 953 |
| ENSP00000245960 | CDC25B | 10 | 0.651 | 0.000292 | 308 |
| ENSP00000287497 | ITGAM | 522 | 0.658 | 0.015227 | 945 |
| ENSP00000295897 | ALB | 1049 | 0.663 | 0.030599 | 994 |
| ENSP00000263309 | CLNS1A | 59 | 0.664 | 0.001721 | 0 |
| ENSP00000251849 | RAF1 | 675 | 0.668 | 0.01969 | 400 |
| ENSP00000300093 | PLK1 | 487 | 0.673 | 0.014206 | 429 |
| ENSP00000319169 | PRMT5 | 59 | 0.673 | 0.001721 | 633 |
| ENSP00000301633 | BIRC5 | 9 | 0.674 | 0.000263 | 927 |
| ENSP00000300134 | STAT6 | 185 | 0.681 | 0.005396 | 913 |
| ENSP00000336790 | ATF4 | 280 | 0.684 | 0.008168 | 995 |
| ENSP00000326031 | PPP1CA | 2 | 0.691 | 5.83E-05 | 654 |
| ENSP00000295797 | PRKCI | 78 | 0.7 | 0.002275 | 899 |
| ENSP00000378165 | ZNF207 | 57 | 0.7 | 0.001663 | 0 |
| ENSP00000314458 | CDC42 | 629 | 0.712 | 0.018348 | 937 |
| ENSP00000011653 | CD4 | 113 | 0.718 | 0.003296 | 855 |
| ENSP00000361626 | YBX1 | 10 | 0.72 | 0.000292 | 860 |
| ENSP00000310127 | IRF3 | 20 | 0.721 | 0.000583 | 462 |
| ENSP00000359206 | BTRC | 38 | 0.726 | 0.001108 | 930 |
| ENSP00000339109 | ANAPC1 | 21 | 0.727 | 0.000613 | 0 |
| ENSP00000318861 | SF3B2 | 1 | 0.729 | 2.92E-05 | 201 |
| ENSP00000046794 | LCP2 | 186 | 0.735 | 0.005426 | 844 |
| ENSP00000268712 | NCOR1 | 64 | 0.741 | 0.001867 | 989 |
| ENSP00000357858 | BUB3 | 57 | 0.744 | 0.001663 | 300 |
| ENSP00000367408 | CASK | 256 | 0.745 | 0.007467 | 387 |
| ENSP00000264033 | CBL | 7043 | 0.755 | 0.205443 | 996 |
| ENSP00000312999 | GNAI2 | 3 | 0.755 | 8.75E-05 | 917 |
| ENSP00000302269 | VAV1 | 27 | 0.758 | 0.000788 | 933 |
| ENSP00000252444 | LDLR | 31 | 0.772 | 0.000904 | 894 |
| ENSP00000223095 | SERPINE1 | 297 | 0.774 | 0.008663 | 987 |
| ENSP00000333194 | RGS19 | 3 | 0.779 | 8.75E-05 | 902 |
| ENSP00000303242 | ITGB2 | 633 | 0.78 | 0.018465 | 962 |
| ENSP00000349467 | CALM1 | 122 | 0.78 | 0.003559 | 688 |
| ENSP00000259808 | RIPK1 | 366 | 0.781 | 0.010676 | 374 |
| ENSP00000326804 | CUL1 | 8 | 0.783 | 0.000233 | 959 |
| ENSP00000367316 | ITGA8 | 632 | 0.787 | 0.018435 | 820 |
| ENSP00000293379 | ITGA5 | 1617 | 0.789 | 0.047168 | 919 |
| ENSP00000261461 | PPP2R5A | 130 | 0.79 | 0.003792 | 924 |
| ENSP00000293272 | CCL5 | 111 | 0.792 | 0.003238 | 927 |
| ENSP00000304895 | IRS1 | 559 | 0.793 | 0.016306 | 996 |
| ENSP00000311677 | PPP1R8 | 2 | 0.793 | 5.83E-05 | 0 |
| ENSP00000256443 | CDK7 | 281 | 0.801 | 0.008197 | 981 |
| ENSP00000239223 | DUSP1 | 6 | 0.806 | 0.000175 | 816 |
| ENSP00000374455 | SQSTM1 | 78 | 0.808 | 0.002275 | 913 |
| ENSP00000360683 | PTPN1 | 154 | 0.812 | 0.004492 | 992 |
| ENSP00000260363 | KIF23 | 2 | 0.817 | 5.83E-05 | 279 |
| ENSP00000321656 | CDC25C | 7 | 0.821 | 0.000204 | 229 |
| ENSP00000324804 | PPP2R1A | 158 | 0.829 | 0.004609 | 817 |
| ENSP00000348986 | INS-IGF2 | 276 | 0.838 | 0.008051 | 982 |
| ENSP00000264246 | CD80 | 64 | 0.843 | 0.001867 | 609 |
| ENSP00000313829 | KHDRBS1 | 449 | 0.846 | 0.013097 | 374 |
| ENSP00000226218 | SEBOX | 87 | 0.853 | 0.002538 | 983 |
| ENSP00000324890 | CD28 | 64 | 0.856 | 0.001867 | 619 |
| ENSP00000384273 | RELA | 88 | 0.856 | 0.002567 | 978 |
| ENSP00000348554 | CDC16 | 2 | 0.867 | 5.83E-05 | 0 |
| ENSP00000303830 | INSR | 810 | 0.875 | 0.023628 | 997 |
| ENSP00000223023 | WASL | 621 | 0.878 | 0.018114 | 256 |
| ENSP00000304592 | FASN | 122 | 0.881 | 0.003559 | 905 |
| ENSP00000265171 | EGF | 339 | 0.882 | 0.009889 | 954 |
| ENSP00000292303 | CCR5 | 111 | 0.884 | 0.003238 | 899 |
| ENSP00000302967 | HDAC3 | 126 | 0.884 | 0.003675 | 991 |
| ENSP00000235090 | WDR77 | 59 | 0.896 | 0.001721 | 196 |
| ENSP00000303939 | CTLA4 | 412 | 0.899 | 0.012018 | 562 |
| ENSP00000371532 | VLDLR | 138 | 0.9 | 0.004025 | 837 |
| ENSP00000303634 | LRP8 | 8 | 0.904 | 0.000233 | 203 |
| ENSP00000266970 | CDK2 | 1021 | 0.91 | 0.029782 | 893 |
| ENSP00000353483 | MAPK8 | 281 | 0.931 | 0.008197 | 940 |
| ENSP00000370473 | IGFBP3 | 908 | 0.932 | 0.026486 | 999 |
| ENSP00000268182 | IQGAP1 | 8 | 0.934 | 0.000233 | 972 |
| ENSP00000269321 | ARHGDIA | 75 | 0.941 | 0.002188 | 924 |
| ENSP00000242577 | DYNLL1 | 93 | 0.957 | 0.002713 | 842 |
| ENSP00000315702 | MOB4 | 93 | 0.962 | 0.002713 | 230 |
| ENSP00000231487 | SKP1 | 543 | 0.968 | 0.015839 | 987 |
| ENSP00000264110 | ATF2 | 116 | 0.99 | 0.003384 | 379 |
| ENSP00000329411 | IRF7 | 3 | 0.994 | 8.75E-05 | 809 |

1. 481 candidate co-regeneration genes of bone and vessel

| **Ensembl ID** | **Gene symbol** | **Betweenness** | **Permutation FDR** | **Betweenness ratio** | **Min-Max interaction score** |
| --- | --- | --- | --- | --- | --- |
| ENSP00000309572 | TERT | 122 | 0.001 | 0.003876 | 923 |
| ENSP00000287139 | NODAL | 1 | 0.003 | 3.18E-05 | 937 |
| ENSP00000384675 | SOS1 | 44 | 0.004 | 0.001398 | 928 |
| ENSP00000262340 | RPE65 | 5 | 0.005 | 0.000159 | 274 |
| ENSP00000360519 | RBP4 | 126 | 0.005 | 0.004003 | 661 |
| ENSP00000261267 | LYZ | 258 | 0.006 | 0.008197 | 748 |
| ENSP00000376609 | GRK5 | 1 | 0.007 | 3.18E-05 | 910 |
| ENSP00000035307 | CHPF2 | 256 | 0.009 | 0.008133 | 899 |
| ENSP00000320709 | ADIPOQ | 122 | 0.009 | 0.003876 | 954 |
| ENSP00000371152 | ASAH1 | 1 | 0.009 | 3.18E-05 | 173 |
| ENSP00000318868 | SHMT1 | 122 | 0.01 | 0.003876 | 363 |
| ENSP00000262188 | SMARCD3 | 121 | 0.012 | 0.003844 | 900 |
| ENSP00000342656 | EXT2 | 1 | 0.012 | 3.18E-05 | 926 |
| ENSP00000295731 | IHH | 486 | 0.014 | 0.01544 | 912 |
| ENSP00000359532 | NTSR1 | 1 | 0.014 | 3.18E-05 | 899 |
| ENSP00000258962 | SRSF1 | 2 | 0.016 | 6.35E-05 | 251 |
| ENSP00000377492 | HMMR | 121 | 0.016 | 0.003844 | 411 |
| ENSP00000337224 | LRAT | 6 | 0.017 | 0.000191 | 928 |
| ENSP00000354476 | SREBF2 | 244 | 0.017 | 0.007752 | 903 |
| ENSP00000384573 | DAZ1 | 1 | 0.017 | 3.18E-05 | 0 |
| ENSP00000317272 | MET | 634 | 0.018 | 0.020142 | 984 |
| ENSP00000256458 | IRAK2 | 122 | 0.019 | 0.003876 | 913 |
| ENSP00000344782 | GFI1B | 1 | 0.019 | 3.18E-05 | 538 |
| ENSP00000361658 | NUP188 | 258 | 0.019 | 0.008197 | 380 |
| ENSP00000339992 | MYB | 258 | 0.02 | 0.008197 | 953 |
| ENSP00000371067 | JAK2 | 853 | 0.021 | 0.0271 | 994 |
| ENSP00000322142 | ING5 | 122 | 0.022 | 0.003876 | 407 |
| ENSP00000362166 | MEAF6 | 122 | 0.022 | 0.003876 | 193 |
| ENSP00000327246 | VIPR1 | 115 | 0.023 | 0.003654 | 899 |
| ENSP00000156825 | MBD3 | 122 | 0.024 | 0.003876 | 360 |
| ENSP00000300403 | TPX2 | 111 | 0.024 | 0.003526 | 205 |
| ENSP00000316042 | HNRNPA0 | 110 | 0.025 | 0.003495 | 171 |
| ENSP00000342905 | ADNP | 122 | 0.025 | 0.003876 | 159 |
| ENSP00000351605 | FZD6 | 258 | 0.026 | 0.008197 | 906 |
| ENSP00000361066 | NCOA3 | 78 | 0.026 | 0.002478 | 950 |
| ENSP00000419692 | RXRA | 353 | 0.026 | 0.011215 | 964 |
| ENSP00000303864 | OR8I2 | 122 | 0.029 | 0.003876 | 0 |
| ENSP00000162749 | TNFRSF1A | 291 | 0.03 | 0.009245 | 931 |
| ENSP00000229390 | SRSF9 | 122 | 0.03 | 0.003876 | 0 |
| ENSP00000302564 | BCL2L1 | 2 | 0.03 | 6.35E-05 | 892 |
| ENSP00000244520 | SNRPC | 118 | 0.031 | 0.003749 | 363 |
| ENSP00000296181 | ITGB5 | 6 | 0.031 | 0.000191 | 809 |
| ENSP00000337014 | HFE2 | 20 | 0.031 | 0.000635 | 938 |
| ENSP00000368174 | MCM8 | 255 | 0.031 | 0.008101 | 551 |
| ENSP00000370526 | ARSE | 2 | 0.031 | 6.35E-05 | 374 |
| ENSP00000245919 | FOSB | 79 | 0.033 | 0.00251 | 928 |
| ENSP00000247225 | SGPP1 | 121 | 0.034 | 0.003844 | 193 |
| ENSP00000269397 | CBX4 | 120 | 0.034 | 0.003812 | 391 |
| ENSP00000312122 | SEC13 | 122 | 0.034 | 0.003876 | 156 |
| ENSP00000413720 | CDKN1C | 122 | 0.035 | 0.003876 | 846 |
| ENSP00000222399 | LAMB1 | 118 | 0.036 | 0.003749 | 271 |
| ENSP00000269886 | SH3GL1 | 117 | 0.036 | 0.003717 | 800 |
| ENSP00000316032 | NUP98 | 122 | 0.036 | 0.003876 | 227 |
| ENSP00000334003 | INTU | 122 | 0.036 | 0.003876 | 380 |
| ENSP00000276571 | CRH | 4 | 0.037 | 0.000127 | 932 |
| ENSP00000312697 | DMAP1 | 243 | 0.037 | 0.00772 | 193 |
| ENSP00000264634 | WNT5A | 1 | 0.039 | 3.18E-05 | 989 |
| ENSP00000338127 | TESK1 | 121 | 0.039 | 0.003844 | 241 |
| ENSP00000315644 | TYMS | 122 | 0.041 | 0.003876 | 480 |
| ENSP00000386741 | CHN1 | 122 | 0.041 | 0.003876 | 286 |
| ENSP00000263269 | GRIN2D | 1 | 0.042 | 3.18E-05 | 406 |
| ENSP00000338477 | HNRNPF | 110 | 0.042 | 0.003495 | 204 |
| ENSP00000286332 | TAB2 | 121 | 0.043 | 0.003844 | 905 |
| ENSP00000355245 | PAX9 | 122 | 0.043 | 0.003876 | 906 |
| ENSP00000317337 | CD300LB | 114 | 0.045 | 0.003622 | 187 |
| ENSP00000299855 | MMP3 | 258 | 0.046 | 0.008197 | 933 |
| ENSP00000302150 | PRL | 293 | 0.046 | 0.009309 | 965 |
| ENSP00000354360 | LAMC3 | 122 | 0.047 | 0.003876 | 0 |
| ENSP00000247843 | YEATS4 | 122 | 0.049 | 0.003876 | 274 |
| ENSP00000269593 | IGFBP4 | 258 | 0.049 | 0.008197 | 999 |
| ENSP00000331736 | SELE | 258 | 0.049 | 0.008197 | 927 |
| ENSP00000373340 | BRPF1 | 122 | 0.049 | 0.003876 | 0 |
| ENSP00000228682 | GLI1 | 374 | 0.05 | 0.011882 | 978 |
| ENSP00000287598 | BUB1B | 36 | 0.051 | 0.001144 | 227 |
| ENSP00000364802 | HSPA1A | 122 | 0.051 | 0.003876 | 624 |
| ENSP00000372224 | HGFAC | 122 | 0.051 | 0.003876 | 872 |
| ENSP00000311697 | FGF5 | 258 | 0.053 | 0.008197 | 966 |
| ENSP00000233813 | IGFBP5 | 258 | 0.054 | 0.008197 | 999 |
| ENSP00000350369 | MAFG | 122 | 0.054 | 0.003876 | 619 |
| ENSP00000316244 | HTR1A | 254 | 0.056 | 0.00807 | 260 |
| ENSP00000358309 | EPHA7 | 122 | 0.059 | 0.003876 | 850 |
| ENSP00000342392 | MESP2 | 515 | 0.06 | 0.016362 | 613 |
| ENSP00000359345 | RPL5 | 258 | 0.06 | 0.008197 | 373 |
| ENSP00000164227 | BCL3 | 258 | 0.061 | 0.008197 | 619 |
| ENSP00000247668 | TRAF2 | 655 | 0.062 | 0.02081 | 768 |
| ENSP00000327048 | MAF | 122 | 0.062 | 0.003876 | 680 |
| ENSP00000250003 | MYOD1 | 954 | 0.064 | 0.030309 | 970 |
| ENSP00000303212 | SEMA3E | 122 | 0.064 | 0.003876 | 552 |
| ENSP00000216181 | MYH9 | 122 | 0.065 | 0.003876 | 363 |
| ENSP00000331831 | GAS6 | 15 | 0.065 | 0.000477 | 235 |
| ENSP00000353720 | CES1 | 1 | 0.067 | 3.18E-05 | 585 |
| ENSP00000236147 | SELL | 122 | 0.068 | 0.003876 | 896 |
| ENSP00000334008 | PARVA | 121 | 0.069 | 0.003844 | 266 |
| ENSP00000351163 | COL11A1 | 1 | 0.069 | 3.18E-05 | 905 |
| ENSP00000290921 | CTBP1 | 120 | 0.07 | 0.003812 | 942 |
| ENSP00000260605 | DYNC2LI1 | 378 | 0.076 | 0.012009 | 350 |
| ENSP00000329357 | SP1 | 343 | 0.076 | 0.010897 | 988 |
| ENSP00000353847 | WWTR1 | 122 | 0.076 | 0.003876 | 958 |
| ENSP00000378529 | FZR1 | 1 | 0.076 | 3.18E-05 | 0 |
| ENSP00000382373 | DAZL | 1 | 0.08 | 3.18E-05 | 340 |
| ENSP00000354621 | SMURF1 | 115 | 0.081 | 0.003654 | 961 |
| ENSP00000360672 | PARD6B | 41 | 0.082 | 0.001303 | 257 |
| ENSP00000361626 | YBX1 | 2 | 0.082 | 6.35E-05 | 860 |
| ENSP00000251772 | PLXNA1 | 4 | 0.083 | 0.000127 | 946 |
| ENSP00000365851 | BMI1 | 120 | 0.085 | 0.003812 | 603 |
| ENSP00000281928 | MED13L | 121 | 0.087 | 0.003844 | 899 |
| ENSP00000217086 | SALL4 | 1 | 0.09 | 3.18E-05 | 814 |
| ENSP00000375863 | HNRNPUL1 | 122 | 0.09 | 0.003876 | 275 |
| ENSP00000220751 | RIPK2 | 122 | 0.091 | 0.003876 | 914 |
| ENSP00000263464 | BIRC3 | 121 | 0.091 | 0.003844 | 831 |
| ENSP00000337103 | CHAT | 258 | 0.095 | 0.008197 | 850 |
| ENSP00000222725 | LFNG | 515 | 0.096 | 0.016362 | 860 |
| ENSP00000259089 | BLK | 119 | 0.097 | 0.003781 | 904 |
| ENSP00000298130 | SPTSSA | 122 | 0.097 | 0.003876 | 176 |
| ENSP00000261507 | MSMO1 | 122 | 0.099 | 0.003876 | 593 |
| ENSP00000189444 | NFKB2 | 157 | 0.101 | 0.004988 | 473 |
| ENSP00000240922 | NAA50 | 122 | 0.101 | 0.003876 | 290 |
| ENSP00000360286 | RAE1 | 379 | 0.102 | 0.012041 | 358 |
| ENSP00000285949 | CYP26C1 | 116 | 0.104 | 0.003685 | 340 |
| ENSP00000268058 | PML | 121 | 0.105 | 0.003844 | 964 |
| ENSP00000356213 | VIP | 122 | 0.105 | 0.003876 | 878 |
| ENSP00000335153 | HSP90AA1 | 988 | 0.106 | 0.031389 | 999 |
| ENSP00000400717 | GNA13 | 121 | 0.107 | 0.003844 | 563 |
| ENSP00000261304 | GALC | 1 | 0.108 | 3.18E-05 | 424 |
| ENSP00000282091 | PTH | 14 | 0.108 | 0.000445 | 962 |
| ENSP00000320940 | NCOA1 | 1159 | 0.11 | 0.036822 | 979 |
| ENSP00000268171 | FURIN | 1 | 0.112 | 3.18E-05 | 938 |
| ENSP00000328777 | EFNA5 | 122 | 0.114 | 0.003876 | 997 |
| ENSP00000279146 | AIP | 122 | 0.115 | 0.003876 | 362 |
| ENSP00000295897 | ALB | 620 | 0.117 | 0.019698 | 980 |
| ENSP00000261908 | NEO1 | 132 | 0.118 | 0.004194 | 297 |
| ENSP00000216225 | RBX1 | 367 | 0.119 | 0.01166 | 959 |
| ENSP00000384442 | CDK11A | 122 | 0.119 | 0.003876 | 402 |
| ENSP00000222382 | CYP3A43 | 3 | 0.122 | 9.53E-05 | 927 |
| ENSP00000256078 | KRAS | 230 | 0.122 | 0.007307 | 926 |
| ENSP00000251968 | TSG101 | 88 | 0.123 | 0.002796 | 195 |
| ENSP00000361162 | TOE1 | 4 | 0.123 | 0.000127 | 0 |
| ENSP00000401980 | MAVS | 122 | 0.124 | 0.003876 | 305 |
| ENSP00000220584 | FDFT1 | 122 | 0.125 | 0.003876 | 899 |
| ENSP00000239938 | EGR1 | 122 | 0.125 | 0.003876 | 930 |
| ENSP00000309913 | TBX5 | 122 | 0.125 | 0.003876 | 888 |
| ENSP00000257904 | CDK4 | 145 | 0.126 | 0.004607 | 839 |
| ENSP00000380942 | ARHGEF12 | 121 | 0.126 | 0.003844 | 259 |
| ENSP00000402240 | KIAA1432 | 122 | 0.126 | 0.003876 | 193 |
| ENSP00000229769 | FANCE | 122 | 0.127 | 0.003876 | 193 |
| ENSP00000231572 | RARS | 1 | 0.128 | 3.18E-05 | 878 |
| ENSP00000254066 | RARA | 15 | 0.128 | 0.000477 | 902 |
| ENSP00000267163 | RB1 | 424 | 0.129 | 0.013471 | 972 |
| ENSP00000245907 | C3 | 122 | 0.131 | 0.003876 | 899 |
| ENSP00000387662 | GCG | 352 | 0.131 | 0.011183 | 986 |
| ENSP00000262554 | SPTLC1 | 122 | 0.133 | 0.003876 | 367 |
| ENSP00000376076 | SUMO1 | 29 | 0.133 | 0.000921 | 943 |
| ENSP00000327758 | NKX2-5 | 121 | 0.137 | 0.003844 | 600 |
| ENSP00000345571 | E2F1 | 6 | 0.137 | 0.000191 | 916 |
| ENSP00000332353 | PTCH1 | 2022 | 0.138 | 0.064239 | 998 |
| ENSP00000254719 | RPA1 | 88 | 0.141 | 0.002796 | 502 |
| ENSP00000354511 | COMT | 3 | 0.141 | 9.53E-05 | 609 |
| ENSP00000305416 | S1PR1 | 14 | 0.142 | 0.000445 | 985 |
| ENSP00000350878 | S1PR3 | 235 | 0.142 | 0.007466 | 899 |
| ENSP00000380252 | NFE2L2 | 122 | 0.142 | 0.003876 | 922 |
| ENSP00000227507 | CCND1 | 1320 | 0.143 | 0.041937 | 991 |
| ENSP00000354394 | STAT1 | 140 | 0.145 | 0.004448 | 994 |
| ENSP00000316854 | ATOX1 | 122 | 0.146 | 0.003876 | 0 |
| ENSP00000363868 | ABCA1 | 122 | 0.147 | 0.003876 | 954 |
| ENSP00000400365 | LAMA2 | 240 | 0.149 | 0.007625 | 314 |
| ENSP00000271688 | CERS2 | 258 | 0.15 | 0.008197 | 235 |
| ENSP00000350616 | DDC | 377 | 0.151 | 0.011977 | 963 |
| ENSP00000263208 | HIRA | 122 | 0.154 | 0.003876 | 303 |
| ENSP00000338072 | AVPR2 | 1 | 0.154 | 3.18E-05 | 901 |
| ENSP00000365858 | GATA1 | 1 | 0.154 | 3.18E-05 | 734 |
| ENSP00000335544 | CCKBR | 121 | 0.157 | 0.003844 | 902 |
| ENSP00000340330 | KAT5 | 243 | 0.157 | 0.00772 | 992 |
| ENSP00000348965 | DYNC1H1 | 121 | 0.158 | 0.003844 | 480 |
| ENSP00000001008 | FKBP4 | 122 | 0.159 | 0.003876 | 677 |
| ENSP00000335657 | CCK | 124 | 0.159 | 0.00394 | 914 |
| ENSP00000305692 | GAA | 139 | 0.161 | 0.004416 | 833 |
| ENSP00000231449 | IL4 | 122 | 0.162 | 0.003876 | 919 |
| ENSP00000360372 | CYP2C19 | 1 | 0.162 | 3.18E-05 | 949 |
| ENSP00000351905 | TGFBR2 | 122 | 0.163 | 0.003876 | 827 |
| ENSP00000204604 | CHRD | 122 | 0.166 | 0.003876 | 997 |
| ENSP00000281708 | FBXW7 | 72 | 0.169 | 0.002287 | 899 |
| ENSP00000302955 | RRM2 | 10 | 0.169 | 0.000318 | 824 |
| ENSP00000272190 | REN | 122 | 0.171 | 0.003876 | 947 |
| ENSP00000296785 | ANKRA2 | 2 | 0.173 | 6.35E-05 | 207 |
| ENSP00000308208 | MMP14 | 256 | 0.174 | 0.008133 | 916 |
| ENSP00000368438 | PCNA | 365 | 0.174 | 0.011596 | 900 |
| ENSP00000229307 | NANOG | 250 | 0.175 | 0.007943 | 877 |
| ENSP00000356024 | CR2 | 118 | 0.176 | 0.003749 | 373 |
| ENSP00000364336 | TBXA2R | 121 | 0.176 | 0.003844 | 928 |
| ENSP00000252444 | LDLR | 28 | 0.179 | 0.00089 | 894 |
| ENSP00000346294 | S100A4 | 122 | 0.179 | 0.003876 | 690 |
| ENSP00000358903 | CYP17A1 | 1 | 0.181 | 3.18E-05 | 281 |
| ENSP00000370571 | TH | 375 | 0.182 | 0.011914 | 888 |
| ENSP00000360247 | CYP2J2 | 119 | 0.183 | 0.003781 | 951 |
| ENSP00000306245 | FOS | 2595 | 0.184 | 0.082444 | 994 |
| ENSP00000296543 | NAA15 | 18 | 0.185 | 0.000572 | 279 |
| ENSP00000343619 | HOXA9 | 122 | 0.186 | 0.003876 | 800 |
| ENSP00000360683 | PTPN1 | 123 | 0.186 | 0.003908 | 992 |
| ENSP00000206249 | ESR1 | 3345 | 0.187 | 0.106271 | 994 |
| ENSP00000264914 | ARSB | 1 | 0.187 | 3.18E-05 | 287 |
| ENSP00000293549 | WNT1 | 255 | 0.188 | 0.008101 | 972 |
| ENSP00000332468 | TRAF3 | 120 | 0.189 | 0.003812 | 684 |
| ENSP00000299293 | FRS2 | 16 | 0.193 | 0.000508 | 983 |
| ENSP00000262238 | YY1 | 161 | 0.194 | 0.005115 | 943 |
| ENSP00000318297 | RUVBL1 | 243 | 0.194 | 0.00772 | 800 |
| ENSP00000342007 | CYP1A2 | 2 | 0.195 | 6.35E-05 | 917 |
| ENSP00000373614 | SELPLG | 379 | 0.195 | 0.012041 | 649 |
| ENSP00000266000 | DAXX | 124 | 0.198 | 0.00394 | 621 |
| ENSP00000362649 | HDAC1 | 681 | 0.2 | 0.021636 | 967 |
| ENSP00000295797 | PRKCI | 81 | 0.202 | 0.002573 | 899 |
| ENSP00000363071 | DES | 268 | 0.203 | 0.008514 | 466 |
| ENSP00000364000 | COL5A2 | 377 | 0.203 | 0.011977 | 914 |
| ENSP00000386259 | NEB | 268 | 0.203 | 0.008514 | 436 |
| ENSP00000371432 | PRLR | 289 | 0.205 | 0.009182 | 542 |
| ENSP00000256474 | VHL | 2980 | 0.206 | 0.094675 | 999 |
| ENSP00000355261 | SMG5 | 6 | 0.208 | 0.000191 | 0 |
| ENSP00000222005 | CDC37 | 16 | 0.209 | 0.000508 | 619 |
| ENSP00000226574 | NFKB1 | 101 | 0.209 | 0.003209 | 944 |
| ENSP00000352798 | COL18A1 | 116 | 0.211 | 0.003685 | 970 |
| ENSP00000345681 | GATA2 | 1 | 0.212 | 3.18E-05 | 987 |
| ENSP00000330393 | LEPR | 5 | 0.213 | 0.000159 | 998 |
| ENSP00000306920 | GLB1 | 118 | 0.214 | 0.003749 | 424 |
| ENSP00000264708 | POMC | 7 | 0.215 | 0.000222 | 921 |
| ENSP00000292408 | FGFR4 | 258 | 0.215 | 0.008197 | 969 |
| ENSP00000046794 | LCP2 | 51 | 0.217 | 0.00162 | 844 |
| ENSP00000217961 | STS | 1 | 0.217 | 3.18E-05 | 619 |
| ENSP00000316054 | DVL3 | 378 | 0.22 | 0.012009 | 969 |
| ENSP00000336790 | ATF4 | 258 | 0.224 | 0.008197 | 894 |
| ENSP00000295926 | CCNL1 | 119 | 0.226 | 0.003781 | 189 |
| ENSP00000282588 | ITGA1 | 10 | 0.227 | 0.000318 | 918 |
| ENSP00000367207 | MYC | 57 | 0.227 | 0.001811 | 991 |
| ENSP00000264335 | YWHAE | 122 | 0.231 | 0.003876 | 902 |
| ENSP00000352712 | DACH1 | 244 | 0.232 | 0.007752 | 426 |
| ENSP00000344115 | CDH5 | 218 | 0.233 | 0.006926 | 962 |
| ENSP00000168712 | FGF4 | 6 | 0.235 | 0.000191 | 989 |
| ENSP00000313950 | AURKB | 268 | 0.235 | 0.008514 | 404 |
| ENSP00000396127 | RAN | 367 | 0.236 | 0.01166 | 374 |
| ENSP00000294172 | NXF1 | 378 | 0.237 | 0.012009 | 307 |
| ENSP00000278385 | CD44 | 648 | 0.24 | 0.020587 | 936 |
| ENSP00000264156 | MCM6 | 1 | 0.243 | 3.18E-05 | 240 |
| ENSP00000355778 | H3F3A | 122 | 0.244 | 0.003876 | 958 |
| ENSP00000312435 | DAG1 | 240 | 0.246 | 0.007625 | 335 |
| ENSP00000331358 | GAST | 121 | 0.247 | 0.003844 | 914 |
| ENSP00000229595 | ASF1A | 122 | 0.248 | 0.003876 | 489 |
| ENSP00000361423 | ABL1 | 87 | 0.251 | 0.002764 | 832 |
| ENSP00000348108 | KHDRBS3 | 118 | 0.252 | 0.003749 | 469 |
| ENSP00000348577 | RANGAP1 | 367 | 0.252 | 0.01166 | 263 |
| ENSP00000246071 | SNRPB2 | 134 | 0.253 | 0.004257 | 216 |
| ENSP00000341551 | SMAD4 | 1864 | 0.254 | 0.05922 | 998 |
| ENSP00000311430 | RPL4 | 258 | 0.261 | 0.008197 | 422 |
| ENSP00000329411 | IRF7 | 7 | 0.261 | 0.000222 | 345 |
| ENSP00000302728 | GUSB | 118 | 0.263 | 0.003749 | 374 |
| ENSP00000315955 | FOXA2 | 4 | 0.263 | 0.000127 | 937 |
| ENSP00000340944 | PTPN11 | 633 | 0.263 | 0.020111 | 993 |
| ENSP00000326630 | ZFPM1 | 1 | 0.264 | 3.18E-05 | 419 |
| ENSP00000314458 | CDC42 | 63 | 0.265 | 0.002002 | 937 |
| ENSP00000372793 | LTA | 172 | 0.265 | 0.005464 | 269 |
| ENSP00000254480 | SMARCC1 | 121 | 0.267 | 0.003844 | 680 |
| ENSP00000255764 | MED10 | 121 | 0.269 | 0.003844 | 902 |
| ENSP00000389140 | DCC | 220 | 0.272 | 0.006989 | 608 |
| ENSP00000417763 | NAA10 | 18 | 0.272 | 0.000572 | 999 |
| ENSP00000313419 | CD19 | 118 | 0.273 | 0.003749 | 947 |
| ENSP00000228837 | FGF6 | 998 | 0.275 | 0.031707 | 999 |
| ENSP00000265056 | MCM2 | 119 | 0.277 | 0.003781 | 229 |
| ENSP00000287934 | FZD1 | 122 | 0.278 | 0.003876 | 979 |
| ENSP00000170630 | IL4R | 312 | 0.279 | 0.009912 | 904 |
| ENSP00000339007 | GRB2 | 1157 | 0.28 | 0.036758 | 990 |
| ENSP00000349467 | CALM1 | 111 | 0.291 | 0.003526 | 688 |
| ENSP00000256010 | NTS | 1 | 0.293 | 3.18E-05 | 913 |
| ENSP00000369442 | KL | 114 | 0.294 | 0.003622 | 599 |
| ENSP00000352400 | NUP214 | 369 | 0.295 | 0.011723 | 219 |
| ENSP00000309103 | BAD | 2 | 0.303 | 6.35E-05 | 919 |
| ENSP00000348786 | RAP1A | 244 | 0.305 | 0.007752 | 340 |
| ENSP00000276420 | DOK2 | 344 | 0.307 | 0.010929 | 989 |
| ENSP00000259371 | DAB2IP | 257 | 0.308 | 0.008165 | 356 |
| ENSP00000322788 | MMP1 | 4 | 0.312 | 0.000127 | 946 |
| ENSP00000344468 | SDC3 | 259 | 0.314 | 0.008228 | 929 |
| ENSP00000223129 | RPA3 | 88 | 0.318 | 0.002796 | 0 |
| ENSP00000265171 | EGF | 131 | 0.319 | 0.004162 | 954 |
| ENSP00000352980 | HIST1H4A | 46 | 0.319 | 0.001461 | 718 |
| ENSP00000225831 | CCL2 | 114 | 0.322 | 0.003622 | 976 |
| ENSP00000363822 | AR | 5 | 0.323 | 0.000159 | 902 |
| ENSP00000352516 | DNMT1 | 5 | 0.324 | 0.000159 | 844 |
| ENSP00000303522 | TACR1 | 1 | 0.325 | 3.18E-05 | 908 |
| ENSP00000332973 | SMAD3 | 1579 | 0.327 | 0.050165 | 946 |
| ENSP00000296585 | ITGA2 | 978 | 0.331 | 0.031071 | 852 |
| ENSP00000262643 | CCNE1 | 32 | 0.332 | 0.001017 | 861 |
| ENSP00000326550 | TACC3 | 1 | 0.332 | 3.18E-05 | 502 |
| ENSP00000334122 | FGF3 | 377 | 0.333 | 0.011977 | 999 |
| ENSP00000290663 | MED8 | 121 | 0.34 | 0.003844 | 899 |
| ENSP00000366466 | CTNNBIP1 | 117 | 0.344 | 0.003717 | 734 |
| ENSP00000262965 | TCF3 | 242 | 0.345 | 0.007688 | 502 |
| ENSP00000268296 | ITGAX | 3 | 0.346 | 9.53E-05 | 384 |
| ENSP00000391349 | DOM3Z | 5 | 0.348 | 0.000159 | 0 |
| ENSP00000309845 | HRAS | 345 | 0.351 | 0.010961 | 989 |
| ENSP00000362441 | ATRX | 122 | 0.351 | 0.003876 | 358 |
| ENSP00000351908 | MAP3K5 | 257 | 0.353 | 0.008165 | 306 |
| ENSP00000366013 | GNB2L1 | 25 | 0.359 | 0.000794 | 997 |
| ENSP00000262435 | SMURF2 | 27 | 0.361 | 0.000858 | 933 |
| ENSP00000264554 | SHC2 | 227 | 0.361 | 0.007212 | 967 |
| ENSP00000218388 | TIMP1 | 279 | 0.364 | 0.008864 | 962 |
| ENSP00000369889 | COL2A1 | 1 | 0.368 | 3.18E-05 | 932 |
| ENSP00000300134 | STAT6 | 312 | 0.369 | 0.009912 | 913 |
| ENSP00000304845 | UGT1A1 | 3 | 0.371 | 9.53E-05 | 902 |
| ENSP00000276201 | UPF3B | 8 | 0.373 | 0.000254 | 296 |
| ENSP00000323050 | RBBP8 | 62 | 0.375 | 0.00197 | 228 |
| ENSP00000260130 | SDCBP | 121 | 0.378 | 0.003844 | 540 |
| ENSP00000257770 | NT5E | 2 | 0.379 | 6.35E-05 | 899 |
| ENSP00000264606 | HDAC4 | 52 | 0.379 | 0.001652 | 974 |
| ENSP00000324648 | CYP2B6 | 3 | 0.383 | 9.53E-05 | 951 |
| ENSP00000278568 | PAK1 | 1 | 0.39 | 3.18E-05 | 857 |
| ENSP00000223023 | WASL | 63 | 0.392 | 0.002002 | 256 |
| ENSP00000340820 | MAPT | 120 | 0.395 | 0.003812 | 981 |
| ENSP00000357392 | EFNA1 | 253 | 0.395 | 0.008038 | 999 |
| ENSP00000231487 | SKP1 | 660 | 0.396 | 0.020968 | 987 |
| ENSP00000348827 | THRB | 1 | 0.403 | 3.18E-05 | 905 |
| ENSP00000262768 | TIMP2 | 255 | 0.406 | 0.008101 | 611 |
| ENSP00000262629 | TYROBP | 114 | 0.409 | 0.003622 | 630 |
| ENSP00000363763 | EPHB2 | 21 | 0.409 | 0.000667 | 729 |
| ENSP00000274026 | CCNA2 | 2 | 0.412 | 6.35E-05 | 426 |
| ENSP00000302269 | VAV1 | 145 | 0.412 | 0.004607 | 933 |
| ENSP00000307387 | PDCD6IP | 117 | 0.414 | 0.003717 | 0 |
| ENSP00000354791 | DCTN1 | 378 | 0.42 | 0.012009 | 620 |
| ENSP00000265023 | KNG1 | 362 | 0.422 | 0.011501 | 985 |
| ENSP00000363998 | ITCH | 121 | 0.424 | 0.003844 | 800 |
| ENSP00000338868 | PHF8 | 3 | 0.428 | 9.53E-05 | 550 |
| ENSP00000337088 | MEN1 | 122 | 0.431 | 0.003876 | 675 |
| ENSP00000288986 | NCK1 | 1 | 0.432 | 3.18E-05 | 983 |
| ENSP00000292303 | CCR5 | 33 | 0.432 | 0.001048 | 899 |
| ENSP00000287647 | FANCD2 | 122 | 0.439 | 0.003876 | 317 |
| ENSP00000370938 | CDK8 | 48 | 0.439 | 0.001525 | 900 |
| ENSP00000308450 | CDC20 | 324 | 0.44 | 0.010294 | 283 |
| ENSP00000345008 | FBLN5 | 122 | 0.44 | 0.003876 | 825 |
| ENSP00000301633 | BIRC5 | 4 | 0.441 | 0.000127 | 927 |
| ENSP00000302961 | HSPA4 | 136 | 0.443 | 0.004321 | 999 |
| ENSP00000215829 | SNRPD3 | 44 | 0.447 | 0.001398 | 269 |
| ENSP00000354586 | GLI2 | 3 | 0.449 | 9.53E-05 | 997 |
| ENSP00000311113 | JUP | 3 | 0.451 | 9.53E-05 | 977 |
| ENSP00000373952 | FANCA | 122 | 0.453 | 0.003876 | 269 |
| ENSP00000398597 | EXOSC6 | 5 | 0.453 | 0.000159 | 0 |
| ENSP00000216223 | IL2RB | 66 | 0.455 | 0.002097 | 424 |
| ENSP00000252818 | JUND | 122 | 0.455 | 0.003876 | 890 |
| ENSP00000237837 | FGF23 | 630 | 0.456 | 0.020015 | 988 |
| ENSP00000334458 | GATA4 | 241 | 0.456 | 0.007657 | 887 |
| ENSP00000351665 | CLIP1 | 377 | 0.459 | 0.011977 | 412 |
| ENSP00000405934 | ITPR1 | 111 | 0.461 | 0.003526 | 308 |
| ENSP00000400591 | SNRPE | 3 | 0.464 | 9.53E-05 | 0 |
| ENSP00000303706 | CDC25A | 1 | 0.466 | 3.18E-05 | 303 |
| ENSP00000229854 | MCM3 | 30 | 0.471 | 0.000953 | 619 |
| ENSP00000366396 | XRN2 | 2 | 0.483 | 6.35E-05 | 191 |
| ENSP00000282441 | YAP1 | 122 | 0.485 | 0.003876 | 899 |
| ENSP00000371973 | SAP18 | 2 | 0.5 | 6.35E-05 | 899 |
| ENSP00000381331 | HDAC2 | 206 | 0.506 | 0.006545 | 761 |
| ENSP00000256452 | IL5RA | 121 | 0.511 | 0.003844 | 375 |
| ENSP00000339151 | IKBKB | 13 | 0.514 | 0.000413 | 908 |
| ENSP00000263686 | SELP | 499 | 0.516 | 0.015853 | 906 |
| ENSP00000312995 | CLSPN | 1 | 0.519 | 3.18E-05 | 219 |
| ENSP00000223029 | AIMP2 | 121 | 0.522 | 0.003844 | 260 |
| ENSP00000382004 | CTNND1 | 40 | 0.522 | 0.001271 | 936 |
| ENSP00000361418 | IPO13 | 80 | 0.524 | 0.002542 | 200 |
| ENSP00000231509 | NR3C1 | 88 | 0.529 | 0.002796 | 917 |
| ENSP00000247161 | ELK1 | 12 | 0.53 | 0.000381 | 905 |
| ENSP00000364893 | ARHGEF7 | 89 | 0.533 | 0.002828 | 372 |
| ENSP00000292644 | PSMC2 | 141 | 0.534 | 0.00448 | 415 |
| ENSP00000227378 | HSPA8 | 175 | 0.536 | 0.00556 | 619 |
| ENSP00000162330 | BCAR1 | 148 | 0.543 | 0.004702 | 925 |
| ENSP00000374455 | SQSTM1 | 81 | 0.545 | 0.002573 | 913 |
| ENSP00000293379 | ITGA5 | 1789 | 0.547 | 0.056837 | 919 |
| ENSP00000222812 | STX1A | 122 | 0.556 | 0.003876 | 287 |
| ENSP00000398698 | TNF | 120 | 0.557 | 0.003812 | 963 |
| ENSP00000362820 | SRSF3 | 4 | 0.559 | 0.000127 | 188 |
| ENSP00000209728 | CDC6 | 257 | 0.565 | 0.008165 | 404 |
| ENSP00000284811 | TCEB1 | 25 | 0.576 | 0.000794 | 999 |
| ENSP00000324897 | UBE2I | 422 | 0.582 | 0.013407 | 876 |
| ENSP00000219548 | STUB1 | 194 | 0.587 | 0.006163 | 621 |
| ENSP00000332643 | NDN | 6 | 0.588 | 0.000191 | 923 |
| ENSP00000353483 | MAPK8 | 74 | 0.588 | 0.002351 | 940 |
| ENSP00000343204 | JAK1 | 69 | 0.589 | 0.002192 | 963 |
| ENSP00000350275 | HIST1H3A | 125 | 0.592 | 0.003971 | 900 |
| ENSP00000300161 | YWHAB | 5 | 0.594 | 0.000159 | 905 |
| ENSP00000297518 | CDK5 | 120 | 0.598 | 0.003812 | 646 |
| ENSP00000358022 | MCL1 | 174 | 0.602 | 0.005528 | 906 |
| ENSP00000216911 | AURKA | 112 | 0.603 | 0.003558 | 915 |
| ENSP00000229022 | VDR | 694 | 0.609 | 0.022049 | 933 |
| ENSP00000252622 | LSM7 | 12 | 0.61 | 0.000381 | 204 |
| ENSP00000367408 | CASK | 191 | 0.614 | 0.006068 | 387 |
| ENSP00000360798 | EPS15 | 122 | 0.625 | 0.003876 | 810 |
| ENSP00000377141 | ARRB1 | 121 | 0.626 | 0.003844 | 769 |
| ENSP00000248566 | SHFM1 | 141 | 0.635 | 0.00448 | 462 |
| ENSP00000261769 | CDH1 | 229 | 0.637 | 0.007275 | 927 |
| ENSP00000278616 | ATM | 58 | 0.637 | 0.001843 | 800 |
| ENSP00000245960 | CDC25B | 15 | 0.64 | 0.000477 | 308 |
| ENSP00000350877 | SRSF2 | 4 | 0.643 | 0.000127 | 243 |
| ENSP00000256857 | GRP | 17 | 0.648 | 0.00054 | 929 |
| ENSP00000350283 | BRCA1 | 137 | 0.648 | 0.004353 | 792 |
| ENSP00000337825 | LCK | 82 | 0.654 | 0.002605 | 913 |
| ENSP00000326031 | PPP1CA | 4 | 0.663 | 0.000127 | 654 |
| ENSP00000303830 | INSR | 514 | 0.666 | 0.01633 | 997 |
| ENSP00000219476 | TSC2 | 9 | 0.677 | 0.000286 | 998 |
| ENSP00000262803 | UPF1 | 6 | 0.683 | 0.000191 | 232 |
| ENSP00000256442 | CCNB1 | 2743 | 0.685 | 0.087146 | 895 |
| ENSP00000314491 | SRRT | 175 | 0.686 | 0.00556 | 155 |
| ENSP00000278916 | CHEK1 | 1 | 0.687 | 3.18E-05 | 659 |
| ENSP00000222256 | RAB3A | 122 | 0.689 | 0.003876 | 318 |
| ENSP00000311677 | PPP1R8 | 4 | 0.692 | 0.000127 | 0 |
| ENSP00000361850 | PLAU | 35 | 0.694 | 0.001112 | 966 |
| ENSP00000222254 | PIK3R2 | 9 | 0.695 | 0.000286 | 904 |
| ENSP00000359206 | BTRC | 40 | 0.699 | 0.001271 | 930 |
| ENSP00000248572 | GNGT1 | 122 | 0.704 | 0.003876 | 340 |
| ENSP00000310127 | IRF3 | 2 | 0.706 | 6.35E-05 | 462 |
| ENSP00000301019 | CDT1 | 1 | 0.712 | 3.18E-05 | 271 |
| ENSP00000263967 | PIK3CA | 87 | 0.714 | 0.002764 | 972 |
| ENSP00000351407 | ARNT | 1048 | 0.715 | 0.033295 | 999 |
| ENSP00000269321 | ARHGDIA | 25 | 0.724 | 0.000794 | 924 |
| ENSP00000312999 | GNAI2 | 2 | 0.733 | 6.35E-05 | 917 |
| ENSP00000314949 | POLR2A | 6 | 0.734 | 0.000191 | 937 |
| ENSP00000011653 | CD4 | 34 | 0.744 | 0.00108 | 855 |
| ENSP00000350941 | SRC | 2598 | 0.747 | 0.082539 | 995 |
| ENSP00000299543 | CTDP1 | 195 | 0.748 | 0.006195 | 899 |
| ENSP00000300093 | PLK1 | 498 | 0.749 | 0.015822 | 429 |
| ENSP00000307046 | SDC2 | 424 | 0.761 | 0.013471 | 939 |
| ENSP00000333194 | RGS19 | 2 | 0.766 | 6.35E-05 | 902 |
| ENSP00000261205 | SYT1 | 122 | 0.773 | 0.003876 | 369 |
| ENSP00000343274 | INTS8 | 141 | 0.78 | 0.00448 | 204 |
| ENSP00000226218 | SEBOX | 114 | 0.786 | 0.003622 | 983 |
| ENSP00000339109 | ANAPC1 | 19 | 0.791 | 0.000604 | 0 |
| ENSP00000348551 | NCOR2 | 2 | 0.794 | 6.35E-05 | 919 |
| ENSP00000338934 | EZR | 820 | 0.796 | 0.026052 | 874 |
| ENSP00000223095 | SERPINE1 | 108 | 0.804 | 0.003431 | 987 |
| ENSP00000262320 | AXIN1 | 1490 | 0.807 | 0.047338 | 999 |
| ENSP00000304895 | IRS1 | 272 | 0.807 | 0.008642 | 996 |
| ENSP00000003084 | CFTR | 1175 | 0.808 | 0.03733 | 847 |
| ENSP00000263309 | CLNS1A | 47 | 0.808 | 0.001493 | 0 |
| ENSP00000296871 | CSF2 | 78 | 0.808 | 0.002478 | 953 |
| ENSP00000342952 | ADCY2 | 3 | 0.808 | 9.53E-05 | 942 |
| ENSP00000384053 | CSF2RB | 78 | 0.808 | 0.002478 | 621 |
| ENSP00000319169 | PRMT5 | 47 | 0.809 | 0.001493 | 633 |
| ENSP00000235090 | WDR77 | 45 | 0.813 | 0.00143 | 277 |
| ENSP00000216797 | NFKBIA | 2 | 0.814 | 6.35E-05 | 950 |
| ENSP00000300651 | MED1 | 124 | 0.814 | 0.00394 | 462 |
| ENSP00000287497 | ITGAM | 498 | 0.815 | 0.015822 | 921 |
| ENSP00000262613 | SLC9A3R1 | 1062 | 0.823 | 0.03374 | 957 |
| ENSP00000303634 | LRP8 | 5 | 0.829 | 0.000159 | 203 |
| ENSP00000310596 | LSM1 | 7 | 0.829 | 0.000222 | 210 |
| ENSP00000366135 | EXOSC10 | 253 | 0.83 | 0.008038 | 172 |
| ENSP00000371532 | VLDLR | 18 | 0.832 | 0.000572 | 837 |
| ENSP00000256443 | CDK7 | 258 | 0.835 | 0.008197 | 387 |
| ENSP00000315859 | RNPS1 | 5 | 0.835 | 0.000159 | 0 |
| ENSP00000251849 | RAF1 | 725 | 0.837 | 0.023033 | 400 |
| ENSP00000380921 | SH3KBP1 | 29 | 0.838 | 0.000921 | 811 |
| ENSP00000369050 | CYP1A1 | 737 | 0.843 | 0.023415 | 918 |
| ENSP00000378165 | ZNF207 | 42 | 0.845 | 0.001334 | 0 |
| ENSP00000233946 | IL1R1 | 257 | 0.849 | 0.008165 | 896 |
| ENSP00000260363 | KIF23 | 3 | 0.849 | 9.53E-05 | 279 |
| ENSP00000249299 | NAA38 | 2 | 0.85 | 6.35E-05 | 0 |
| ENSP00000303242 | ITGB2 | 496 | 0.85 | 0.015758 | 962 |
| ENSP00000309503 | YWHAZ | 9 | 0.852 | 0.000286 | 784 |
| ENSP00000367316 | ITGA8 | 495 | 0.852 | 0.015726 | 820 |
| ENSP00000344818 | UBC | 15477 | 0.857 | 0.491708 | 999 |
| ENSP00000291552 | U2AF1 | 4 | 0.86 | 0.000127 | 316 |
| ENSP00000268712 | NCOR1 | 21 | 0.864 | 0.000667 | 943 |
| ENSP00000240185 | TARDBP | 234 | 0.866 | 0.007434 | 177 |
| ENSP00000357858 | BUB3 | 42 | 0.868 | 0.001334 | 300 |
| ENSP00000414634 | LSM2 | 14 | 0.868 | 0.000445 | 0 |
| ENSP00000348708 | UPF2 | 12 | 0.873 | 0.000381 | 228 |
| ENSP00000221494 | SF3A2 | 396 | 0.881 | 0.012581 | 944 |
| ENSP00000342374 | SNRPD2 | 1 | 0.891 | 3.18E-05 | 241 |
| ENSP00000264033 | CBL | 5713 | 0.894 | 0.181503 | 996 |
| ENSP00000302967 | HDAC3 | 54 | 0.895 | 0.001716 | 991 |
| ENSP00000348554 | CDC16 | 1 | 0.903 | 3.18E-05 | 0 |
| ENSP00000300574 | CRK | 410 | 0.926 | 0.013026 | 953 |
| ENSP00000358997 | IRAK1 | 249 | 0.926 | 0.007911 | 938 |
| ENSP00000379625 | MYD88 | 257 | 0.931 | 0.008165 | 983 |
| ENSP00000266970 | CDK2 | 760 | 0.943 | 0.024145 | 893 |
| ENSP00000370473 | IGFBP3 | 801 | 0.943 | 0.025448 | 999 |
| ENSP00000384273 | RELA | 74 | 0.949 | 0.002351 | 978 |
| ENSP00000313829 | KHDRBS1 | 525 | 0.95 | 0.016679 | 374 |
| ENSP00000307863 | U2AF2 | 464 | 0.967 | 0.014741 | 367 |
| ENSP00000338799 | IL6ST | 410 | 0.977 | 0.013026 | 999 |
| ENSP00000401303 | SHC1 | 1213 | 0.983 | 0.038537 | 994 |
| ENSP00000351486 | NTRK1 | 1220 | 0.994 | 0.03876 | 998 |

1. 417 candidate co-regeneration genes of dentine and nerve

| **Ensembl ID** | **Gene symbol** | **Betweenness** | **P-value** | **Betweenness ratio** | **Min-Max interaction score** |
| --- | --- | --- | --- | --- | --- |
| ENSP00000263409 | LIFR | 27 | <0.001 | 0.003559 | 245 |
| ENSP00000320758 | NOS1 | 54 | <0.001 | 0.007117 | 313 |
| ENSP00000338799 | IL6ST | 57 | <0.001 | 0.007513 | 900 |
| ENSP00000349437 | IGF2R | 4 | <0.001 | 0.000527 | 274 |
| ENSP00000261207 | PPP1R12A | 27 | 0.001 | 0.003559 | 274 |
| ENSP00000263354 | NAPA | 27 | 0.001 | 0.003559 | 0 |
| ENSP00000321239 | RCHY1 | 27 | 0.002 | 0.003559 | 203 |
| ENSP00000340944 | PTPN11 | 83 | 0.002 | 0.01094 | 281 |
| ENSP00000360483 | NDC1 | 280 | 0.004 | 0.036905 | 466 |
| ENSP00000358081 | BAG3 | 27 | 0.01 | 0.003559 | 215 |
| ENSP00000362649 | HDAC1 | 50 | 0.01 | 0.00659 | 993 |
| ENSP00000313809 | AMBN | 280 | 0.012 | 0.036905 | 819 |
| ENSP00000357711 | S100A7 | 27 | 0.012 | 0.003559 | 0 |
| ENSP00000255390 | SCO1 | 280 | 0.013 | 0.036905 | 0 |
| ENSP00000296181 | ITGB5 | 1 | 0.013 | 0.000132 | 825 |
| ENSP00000299335 | COX11 | 280 | 0.013 | 0.036905 | 266 |
| ENSP00000329384 | IL22 | 27 | 0.013 | 0.003559 | 0 |
| ENSP00000231572 | RARS | 281 | 0.015 | 0.037037 | 865 |
| ENSP00000290200 | IL10RB | 27 | 0.015 | 0.003559 | 0 |
| ENSP00000323194 | PLXNA4 | 27 | 0.015 | 0.003559 | 0 |
| ENSP00000296695 | SPINK1 | 27 | 0.016 | 0.003559 | 567 |
| ENSP00000355001 | POU3F3 | 27 | 0.016 | 0.003559 | 260 |
| ENSP00000285968 | NUP205 | 10 | 0.017 | 0.001318 | 0 |
| ENSP00000387286 | RAB1A | 27 | 0.017 | 0.003559 | 188 |
| ENSP00000316845 | ARHGEF4 | 27 | 0.018 | 0.003559 | 0 |
| ENSP00000336762 | ANG | 27 | 0.021 | 0.003559 | 215 |
| ENSP00000338297 | IGF2 | 4 | 0.021 | 0.000527 | 440 |
| ENSP00000361850 | PLAU | 29 | 0.022 | 0.003822 | 380 |
| ENSP00000260228 | MMP20 | 281 | 0.023 | 0.037037 | 213 |
| ENSP00000044462 | PSMA4 | 27 | 0.024 | 0.003559 | 901 |
| ENSP00000253925 | PPFIA1 | 27 | 0.024 | 0.003559 | 0 |
| ENSP00000371973 | SAP18 | 2 | 0.024 | 0.000264 | 174 |
| ENSP00000283228 | PTPRR | 27 | 0.025 | 0.003559 | 0 |
| ENSP00000350877 | SRSF2 | 6 | 0.025 | 0.000791 | 0 |
| ENSP00000339428 | SOCS2 | 14 | 0.027 | 0.001845 | 0 |
| ENSP00000356713 | IFNGR1 | 27 | 0.028 | 0.003559 | 0 |
| ENSP00000295119 | NUP35 | 280 | 0.029 | 0.036905 | 0 |
| ENSP00000406878 | PSMB8 | 27 | 0.029 | 0.003559 | 0 |
| ENSP00000354280 | PRSS3 | 27 | 0.031 | 0.003559 | 502 |
| ENSP00000266085 | TIMP3 | 27 | 0.033 | 0.003559 | 331 |
| ENSP00000387662 | GCG | 55 | 0.033 | 0.007249 | 896 |
| ENSP00000264554 | SHC2 | 339 | 0.034 | 0.044682 | 260 |
| ENSP00000325120 | PGR | 27 | 0.034 | 0.003559 | 860 |
| ENSP00000308741 | CLOCK | 27 | 0.035 | 0.003559 | 979 |
| ENSP00000367462 | OLAH | 27 | 0.036 | 0.003559 | 180 |
| ENSP00000394794 | PTPN13 | 27 | 0.036 | 0.003559 | 156 |
| ENSP00000204961 | EFNB1 | 27 | 0.037 | 0.003559 | 273 |
| ENSP00000250894 | MAPK8IP3 | 27 | 0.039 | 0.003559 | 0 |
| ENSP00000291442 | NR2F6 | 27 | 0.039 | 0.003559 | 902 |
| ENSP00000230882 | GHR | 281 | 0.04 | 0.037037 | 675 |
| ENSP00000323065 | GADD45GIP1 | 27 | 0.04 | 0.003559 | 0 |
| ENSP00000376684 | EPHB6 | 27 | 0.04 | 0.003559 | 0 |
| ENSP00000223029 | AIMP2 | 280 | 0.041 | 0.036905 | 0 |
| ENSP00000246032 | STK35 | 27 | 0.041 | 0.003559 | 204 |
| ENSP00000254657 | PER2 | 26 | 0.041 | 0.003427 | 243 |
| ENSP00000264426 | GRIA2 | 1 | 0.045 | 0.000132 | 0 |
| ENSP00000275874 | RAB19 | 27 | 0.047 | 0.003559 | 204 |
| ENSP00000374357 | ARNTL | 27 | 0.048 | 0.003559 | 900 |
| ENSP00000200453 | PPP1R15A | 27 | 0.049 | 0.003559 | 899 |
| ENSP00000310668 | NUP93 | 280 | 0.049 | 0.036905 | 0 |
| ENSP00000250003 | MYOD1 | 27 | 0.05 | 0.003559 | 848 |
| ENSP00000339151 | IKBKB | 1 | 0.05 | 0.000132 | 749 |
| ENSP00000417864 | ANP32A | 27 | 0.052 | 0.003559 | 0 |
| ENSP00000008527 | CRY1 | 26 | 0.053 | 0.003427 | 193 |
| ENSP00000368020 | KIF3A | 27 | 0.053 | 0.003559 | 0 |
| ENSP00000291552 | U2AF1 | 5 | 0.056 | 0.000659 | 0 |
| ENSP00000216225 | RBX1 | 70 | 0.058 | 0.009226 | 193 |
| ENSP00000236671 | CTSD | 27 | 0.059 | 0.003559 | 412 |
| ENSP00000227507 | CCND1 | 331 | 0.06 | 0.043627 | 946 |
| ENSP00000396127 | RAN | 343 | 0.06 | 0.045209 | 899 |
| ENSP00000377141 | ARRB1 | 27 | 0.061 | 0.003559 | 201 |
| ENSP00000283195 | RANBP2 | 24 | 0.062 | 0.003163 | 170 |
| ENSP00000226730 | IL2 | 4 | 0.064 | 0.000527 | 609 |
| ENSP00000327583 | RANBP1 | 2 | 0.064 | 0.000264 | 422 |
| ENSP00000381066 | MAP2K7 | 27 | 0.064 | 0.003559 | 0 |
| ENSP00000261205 | SYT1 | 27 | 0.067 | 0.003559 | 621 |
| ENSP00000347198 | SRGAP1 | 105 | 0.067 | 0.013839 | 0 |
| ENSP00000348577 | RANGAP1 | 342 | 0.068 | 0.045077 | 0 |
| ENSP00000356918 | STX7 | 27 | 0.068 | 0.003559 | 174 |
| ENSP00000362994 | TRAF1 | 27 | 0.069 | 0.003559 | 201 |
| ENSP00000256857 | GRP | 2 | 0.07 | 0.000264 | 290 |
| ENSP00000393725 | GFRA1 | 81 | 0.07 | 0.010676 | 0 |
| ENSP00000352929 | CSNK1E | 26 | 0.072 | 0.003427 | 900 |
| ENSP00000391069 | SRPK1 | 12 | 0.072 | 0.001582 | 0 |
| ENSP00000357753 | IVL | 27 | 0.074 | 0.003559 | 842 |
| ENSP00000162749 | TNFRSF1A | 269 | 0.076 | 0.035455 | 347 |
| ENSP00000316042 | HNRNPA0 | 9 | 0.076 | 0.001186 | 0 |
| ENSP00000376177 | CALCRL | 27 | 0.078 | 0.003559 | 221 |
| ENSP00000391901 | PHF1 | 18 | 0.078 | 0.002372 | 318 |
| ENSP00000348708 | UPF2 | 9 | 0.079 | 0.001186 | 0 |
| ENSP00000332353 | PTCH1 | 78 | 0.08 | 0.010281 | 878 |
| ENSP00000374372 | SPTB | 26 | 0.082 | 0.003427 | 196 |
| ENSP00000263864 | VAMP8 | 27 | 0.083 | 0.003559 | 0 |
| ENSP00000287934 | FZD1 | 281 | 0.084 | 0.037037 | 896 |
| ENSP00000370343 | IRF4 | 4 | 0.085 | 0.000527 | 285 |
| ENSP00000227667 | APOC3 | 27 | 0.086 | 0.003559 | 918 |
| ENSP00000375986 | MAP3K4 | 27 | 0.086 | 0.003559 | 0 |
| ENSP00000303325 | TACR3 | 2 | 0.087 | 0.000264 | 0 |
| ENSP00000314520 | KCNA2 | 27 | 0.09 | 0.003559 | 0 |
| ENSP00000247668 | TRAF2 | 313 | 0.091 | 0.041255 | 654 |
| ENSP00000257904 | CDK4 | 4 | 0.091 | 0.000527 | 378 |
| ENSP00000261464 | TRAF5 | 27 | 0.091 | 0.003559 | 0 |
| ENSP00000344468 | SDC3 | 27 | 0.091 | 0.003559 | 800 |
| ENSP00000358541 | SIKE1 | 2 | 0.092 | 0.000264 | 0 |
| ENSP00000327647 | CRADD | 27 | 0.095 | 0.003559 | 0 |
| ENSP00000333001 | RBM8A | 1 | 0.095 | 0.000132 | 538 |
| ENSP00000407431 | HLA-C | 6 | 0.095 | 0.000791 | 235 |
| ENSP00000302955 | RRM2 | 1 | 0.097 | 0.000132 | 182 |
| ENSP00000356087 | IKBKE | 2 | 0.098 | 0.000264 | 285 |
| ENSP00000262077 | NUP153 | 270 | 0.099 | 0.035587 | 456 |
| ENSP00000268182 | IQGAP1 | 4 | 0.099 | 0.000527 | 374 |
| ENSP00000282588 | ITGA1 | 27 | 0.099 | 0.003559 | 912 |
| ENSP00000290158 | KPNB1 | 270 | 0.1 | 0.035587 | 0 |
| ENSP00000224237 | VIM | 27 | 0.103 | 0.003559 | 611 |
| ENSP00000229179 | NUP107 | 10 | 0.103 | 0.001318 | 0 |
| ENSP00000355537 | ACTN2 | 27 | 0.103 | 0.003559 | 0 |
| ENSP00000299106 | JAM3 | 27 | 0.104 | 0.003559 | 340 |
| ENSP00000342215 | KIR2DL3 | 6 | 0.104 | 0.000791 | 379 |
| ENSP00000346389 | MEF2A | 1 | 0.104 | 0.000132 | 247 |
| ENSP00000340858 | B2M | 6 | 0.107 | 0.000791 | 427 |
| ENSP00000363071 | DES | 27 | 0.109 | 0.003559 | 532 |
| ENSP00000386259 | NEB | 27 | 0.109 | 0.003559 | 207 |
| ENSP00000376076 | SUMO1 | 26 | 0.11 | 0.003427 | 619 |
| ENSP00000256442 | CCNB1 | 235 | 0.111 | 0.030974 | 749 |
| ENSP00000360525 | MAGOH | 3 | 0.112 | 0.000395 | 0 |
| ENSP00000257430 | APC | 27 | 0.113 | 0.003559 | 626 |
| ENSP00000300093 | PLK1 | 43 | 0.113 | 0.005668 | 305 |
| ENSP00000357927 | BNIPL | 6 | 0.113 | 0.000791 | 0 |
| ENSP00000262435 | SMURF2 | 6 | 0.114 | 0.000791 | 998 |
| ENSP00000309968 | ADAM17 | 27 | 0.114 | 0.003559 | 281 |
| ENSP00000259633 | CD72 | 8 | 0.115 | 0.001054 | 0 |
| ENSP00000362820 | SRSF3 | 6 | 0.115 | 0.000791 | 0 |
| ENSP00000362900 | SRSF4 | 1 | 0.116 | 0.000132 | 0 |
| ENSP00000241014 | MAPK8IP1 | 27 | 0.117 | 0.003559 | 191 |
| ENSP00000265333 | VDAC1 | 26 | 0.117 | 0.003427 | 0 |
| ENSP00000278385 | CD44 | 11 | 0.118 | 0.00145 | 936 |
| ENSP00000362082 | CCND3 | 52 | 0.119 | 0.006854 | 360 |
| ENSP00000350512 | COPS5 | 27 | 0.121 | 0.003559 | 0 |
| ENSP00000284981 | APP | 27 | 0.122 | 0.003559 | 393 |
| ENSP00000264926 | RAD18 | 27 | 0.124 | 0.003559 | 348 |
| ENSP00000332973 | SMAD3 | 556 | 0.126 | 0.073283 | 875 |
| ENSP00000218388 | TIMP1 | 281 | 0.128 | 0.037037 | 505 |
| ENSP00000267163 | RB1 | 12 | 0.128 | 0.001582 | 653 |
| ENSP00000363435 | ITPR3 | 1 | 0.13 | 0.000132 | 946 |
| ENSP00000310491 | ARHGAP1 | 6 | 0.131 | 0.000791 | 203 |
| ENSP00000320147 | EZH2 | 18 | 0.133 | 0.002372 | 542 |
| ENSP00000328511 | KCNA4 | 27 | 0.135 | 0.003559 | 0 |
| ENSP00000314458 | CDC42 | 130 | 0.14 | 0.017135 | 467 |
| ENSP00000293379 | ITGA5 | 588 | 0.141 | 0.077501 | 936 |
| ENSP00000232014 | BCL6 | 281 | 0.142 | 0.037037 | 991 |
| ENSP00000304669 | CTNNA1 | 27 | 0.142 | 0.003559 | 914 |
| ENSP00000261769 | CDH1 | 3 | 0.144 | 0.000395 | 700 |
| ENSP00000354130 | SOX10 | 54 | 0.144 | 0.007117 | 826 |
| ENSP00000330393 | LEPR | 27 | 0.146 | 0.003559 | 867 |
| ENSP00000317714 | STX4 | 27 | 0.147 | 0.003559 | 0 |
| ENSP00000223023 | WASL | 126 | 0.153 | 0.016607 | 0 |
| ENSP00000341344 | GGA1 | 27 | 0.153 | 0.003559 | 163 |
| ENSP00000324897 | UBE2I | 340 | 0.155 | 0.044813 | 644 |
| ENSP00000357392 | EFNA1 | 26 | 0.155 | 0.003427 | 160 |
| ENSP00000279593 | GRIN2B | 27 | 0.16 | 0.003559 | 183 |
| ENSP00000351273 | CASP8 | 27 | 0.16 | 0.003559 | 374 |
| ENSP00000227752 | IL10RA | 27 | 0.165 | 0.003559 | 0 |
| ENSP00000363763 | EPHB2 | 5 | 0.165 | 0.000659 | 200 |
| ENSP00000412237 | IL10 | 27 | 0.165 | 0.003559 | 430 |
| ENSP00000245544 | NUP85 | 27 | 0.168 | 0.003559 | 0 |
| ENSP00000299402 | APBB1 | 10 | 0.168 | 0.001318 | 274 |
| ENSP00000302530 | BUB1 | 7 | 0.168 | 0.000923 | 0 |
| ENSP00000222254 | PIK3R2 | 1 | 0.169 | 0.000132 | 619 |
| ENSP00000349465 | PICK1 | 1 | 0.169 | 0.000132 | 0 |
| ENSP00000263033 | SYTL4 | 27 | 0.175 | 0.003559 | 0 |
| ENSP00000337761 | RAB27A | 27 | 0.175 | 0.003559 | 307 |
| ENSP00000238081 | YWHAQ | 1 | 0.176 | 0.000132 | 923 |
| ENSP00000338934 | EZR | 174 | 0.176 | 0.022934 | 430 |
| ENSP00000242480 | EGR2 | 54 | 0.178 | 0.007117 | 208 |
| ENSP00000289902 | FCER1G | 54 | 0.178 | 0.007117 | 0 |
| ENSP00000327850 | NFATC1 | 54 | 0.178 | 0.007117 | 885 |
| ENSP00000269349 | EIF4A3 | 2 | 0.179 | 0.000264 | 0 |
| ENSP00000296785 | ANKRA2 | 2 | 0.187 | 0.000264 | 0 |
| ENSP00000269485 | TNFRSF11A | 27 | 0.188 | 0.003559 | 341 |
| ENSP00000291700 | S100B | 27 | 0.19 | 0.003559 | 317 |
| ENSP00000310596 | LSM1 | 1 | 0.192 | 0.000132 | 210 |
| ENSP00000363822 | AR | 11 | 0.195 | 0.00145 | 978 |
| ENSP00000265709 | ANK1 | 26 | 0.199 | 0.003427 | 348 |
| ENSP00000267814 | SORD | 26 | 0.199 | 0.003427 | 508 |
| ENSP00000348827 | THRB | 2 | 0.199 | 0.000264 | 907 |
| ENSP00000252997 | GATA5 | 27 | 0.201 | 0.003559 | 274 |
| ENSP00000375921 | PAX3 | 54 | 0.201 | 0.007117 | 818 |
| ENSP00000315644 | TYMS | 27 | 0.203 | 0.003559 | 427 |
| ENSP00000262613 | SLC9A3R1 | 445 | 0.204 | 0.058653 | 892 |
| ENSP00000292644 | PSMC2 | 27 | 0.204 | 0.003559 | 899 |
| ENSP00000162330 | BCAR1 | 82 | 0.206 | 0.010808 | 229 |
| ENSP00000256474 | VHL | 762 | 0.211 | 0.100435 | 165 |
| ENSP00000354554 | MT-CYB | 280 | 0.214 | 0.036905 | 344 |
| ENSP00000340330 | KAT5 | 10 | 0.215 | 0.001318 | 228 |
| ENSP00000228682 | GLI1 | 25 | 0.216 | 0.003295 | 412 |
| ENSP00000302961 | HSPA4 | 35 | 0.216 | 0.004613 | 621 |
| ENSP00000354961 | MT-ND4 | 280 | 0.218 | 0.036905 | 274 |
| ENSP00000411698 | USO1 | 27 | 0.219 | 0.003559 | 165 |
| ENSP00000262320 | AXIN1 | 358 | 0.221 | 0.047186 | 902 |
| ENSP00000052754 | DCN | 103 | 0.225 | 0.013576 | 930 |
| ENSP00000348551 | NCOR2 | 277 | 0.226 | 0.03651 | 986 |
| ENSP00000248244 | TICAM1 | 27 | 0.228 | 0.003559 | 0 |
| ENSP00000315859 | RNPS1 | 5 | 0.229 | 0.000659 | 0 |
| ENSP00000276414 | GNRH1 | 25 | 0.23 | 0.003295 | 432 |
| ENSP00000294172 | NXF1 | 61 | 0.23 | 0.00804 | 258 |
| ENSP00000416097 | GOLGA2 | 27 | 0.23 | 0.003559 | 619 |
| ENSP00000225831 | CCL2 | 2 | 0.234 | 0.000264 | 879 |
| ENSP00000310127 | IRF3 | 4 | 0.234 | 0.000527 | 187 |
| ENSP00000243776 | CHPF | 3 | 0.242 | 0.000395 | 0 |
| ENSP00000343204 | JAK1 | 28 | 0.242 | 0.003691 | 902 |
| ENSP00000228837 | FGF6 | 279 | 0.243 | 0.036773 | 908 |
| ENSP00000284384 | PRKCA | 28 | 0.246 | 0.003691 | 672 |
| ENSP00000384675 | SOS1 | 69 | 0.251 | 0.009095 | 299 |
| ENSP00000364000 | COL5A2 | 1 | 0.252 | 0.000132 | 915 |
| ENSP00000318585 | BACE1 | 27 | 0.254 | 0.003559 | 655 |
| ENSP00000209728 | CDC6 | 7 | 0.255 | 0.000923 | 0 |
| ENSP00000351163 | COL11A1 | 1 | 0.255 | 0.000132 | 900 |
| ENSP00000219476 | TSC2 | 17 | 0.258 | 0.002241 | 953 |
| ENSP00000302564 | BCL2L1 | 27 | 0.259 | 0.003559 | 196 |
| ENSP00000317272 | MET | 54 | 0.26 | 0.007117 | 842 |
| ENSP00000398698 | TNF | 269 | 0.262 | 0.035455 | 942 |
| ENSP00000348786 | RAP1A | 27 | 0.263 | 0.003559 | 177 |
| ENSP00000354876 | MT-CO2 | 280 | 0.263 | 0.036905 | 899 |
| ENSP00000262629 | TYROBP | 5 | 0.267 | 0.000659 | 254 |
| ENSP00000340820 | MAPT | 53 | 0.268 | 0.006986 | 867 |
| ENSP00000351997 | MAP2K6 | 162 | 0.272 | 0.021352 | 167 |
| ENSP00000219548 | STUB1 | 67 | 0.276 | 0.008831 | 621 |
| ENSP00000222812 | STX1A | 135 | 0.281 | 0.017794 | 241 |
| ENSP00000299293 | FRS2 | 2 | 0.291 | 0.000264 | 367 |
| ENSP00000360025 | GADD45A | 54 | 0.295 | 0.007117 | 676 |
| ENSP00000301838 | FADD | 2 | 0.298 | 0.000264 | 219 |
| ENSP00000254066 | RARA | 13 | 0.3 | 0.001713 | 905 |
| ENSP00000260130 | SDCBP | 26 | 0.301 | 0.003427 | 200 |
| ENSP00000361818 | SDC4 | 54 | 0.301 | 0.007117 | 800 |
| ENSP00000303634 | LRP8 | 1 | 0.305 | 0.000132 | 227 |
| ENSP00000222256 | RAB3A | 27 | 0.306 | 0.003559 | 171 |
| ENSP00000300651 | MED1 | 10 | 0.308 | 0.001318 | 765 |
| ENSP00000401303 | SHC1 | 298 | 0.316 | 0.039278 | 924 |
| ENSP00000352400 | NUP214 | 49 | 0.317 | 0.006458 | 210 |
| ENSP00000383623 | MLLT4 | 281 | 0.317 | 0.037037 | 945 |
| ENSP00000391592 | PTPN6 | 9 | 0.32 | 0.001186 | 0 |
| ENSP00000353224 | TFRC | 6 | 0.328 | 0.000791 | 432 |
| ENSP00000351486 | NTRK1 | 311 | 0.335 | 0.040991 | 0 |
| ENSP00000269260 | ARRB2 | 27 | 0.339 | 0.003559 | 240 |
| ENSP00000344818 | UBC | 3966 | 0.341 | 0.522736 | 999 |
| ENSP00000356346 | PTPRC | 19 | 0.341 | 0.002504 | 613 |
| ENSP00000003084 | CFTR | 564 | 0.342 | 0.074338 | 429 |
| ENSP00000287727 | ZFYVE9 | 24 | 0.344 | 0.003163 | 993 |
| ENSP00000311032 | CASP3 | 1 | 0.345 | 0.000132 | 935 |
| ENSP00000417404 | HFE | 6 | 0.345 | 0.000791 | 274 |
| ENSP00000170630 | IL4R | 4 | 0.346 | 0.000527 | 249 |
| ENSP00000343040 | HMGB1 | 54 | 0.348 | 0.007117 | 409 |
| ENSP00000263753 | SGOL1 | 23 | 0.351 | 0.003032 | 0 |
| ENSP00000389140 | DCC | 72 | 0.351 | 0.00949 | 235 |
| ENSP00000242152 | NPY | 27 | 0.356 | 0.003559 | 340 |
| ENSP00000307046 | SDC2 | 46 | 0.356 | 0.006063 | 805 |
| ENSP00000418447 | PPP2CA | 27 | 0.357 | 0.003559 | 229 |
| ENSP00000359988 | SRSF11 | 3 | 0.358 | 0.000395 | 0 |
| ENSP00000361066 | NCOA3 | 45 | 0.361 | 0.005931 | 912 |
| ENSP00000360286 | RAE1 | 27 | 0.363 | 0.003559 | 218 |
| ENSP00000261908 | NEO1 | 33 | 0.367 | 0.00435 | 800 |
| ENSP00000313752 | SSNA1 | 27 | 0.369 | 0.003559 | 162 |
| ENSP00000368438 | PCNA | 112 | 0.369 | 0.014762 | 565 |
| ENSP00000315955 | FOXA2 | 26 | 0.371 | 0.003427 | 809 |
| ENSP00000337014 | HFE2 | 9 | 0.377 | 0.001186 | 260 |
| ENSP00000216911 | AURKA | 27 | 0.38 | 0.003559 | 621 |
| ENSP00000365380 | FOXP3 | 108 | 0.381 | 0.014235 | 385 |
| ENSP00000300574 | CRK | 95 | 0.382 | 0.012521 | 307 |
| ENSP00000304895 | IRS1 | 65 | 0.385 | 0.008567 | 859 |
| ENSP00000296871 | CSF2 | 6 | 0.386 | 0.000791 | 561 |
| ENSP00000316879 | EIF4G1 | 2 | 0.393 | 0.000264 | 752 |
| ENSP00000280892 | EIF4E | 2 | 0.398 | 0.000264 | 228 |
| ENSP00000329967 | TBK1 | 26 | 0.401 | 0.003427 | 285 |
| ENSP00000252945 | CYP2E1 | 1 | 0.403 | 0.000132 | 389 |
| ENSP00000384053 | CSF2RB | 6 | 0.403 | 0.000791 | 0 |
| ENSP00000266970 | CDK2 | 55 | 0.404 | 0.007249 | 988 |
| ENSP00000265023 | KNG1 | 1 | 0.406 | 0.000132 | 379 |
| ENSP00000363921 | PARD3 | 25 | 0.408 | 0.003295 | 924 |
| ENSP00000371532 | VLDLR | 29 | 0.408 | 0.003822 | 205 |
| ENSP00000313950 | AURKB | 27 | 0.409 | 0.003559 | 243 |
| ENSP00000264246 | CD80 | 8 | 0.415 | 0.001054 | 229 |
| ENSP00000367207 | MYC | 35 | 0.416 | 0.004613 | 851 |
| ENSP00000419692 | RXRA | 161 | 0.419 | 0.021221 | 997 |
| ENSP00000258962 | SRSF1 | 14 | 0.424 | 0.001845 | 319 |
| ENSP00000276201 | UPF3B | 9 | 0.426 | 0.001186 | 0 |
| ENSP00000344115 | CDH5 | 6 | 0.427 | 0.000791 | 346 |
| ENSP00000329411 | IRF7 | 2 | 0.429 | 0.000264 | 200 |
| ENSP00000265351 | XPO5 | 1 | 0.431 | 0.000132 | 490 |
| ENSP00000378529 | FZR1 | 7 | 0.432 | 0.000923 | 0 |
| ENSP00000223095 | SERPINE1 | 63 | 0.437 | 0.008304 | 833 |
| ENSP00000271628 | SF3B4 | 26 | 0.442 | 0.003427 | 256 |
| ENSP00000309503 | YWHAZ | 2 | 0.442 | 0.000264 | 969 |
| ENSP00000295797 | PRKCI | 18 | 0.443 | 0.002372 | 193 |
| ENSP00000252102 | NDUFA2 | 280 | 0.454 | 0.036905 | 0 |
| ENSP00000287598 | BUB1B | 19 | 0.454 | 0.002504 | 0 |
| ENSP00000226218 | SEBOX | 19 | 0.46 | 0.002504 | 499 |
| ENSP00000252444 | LDLR | 9 | 0.463 | 0.001186 | 469 |
| ENSP00000367316 | ITGA8 | 82 | 0.463 | 0.010808 | 800 |
| ENSP00000303242 | ITGB2 | 82 | 0.465 | 0.010808 | 340 |
| ENSP00000324890 | CD28 | 8 | 0.465 | 0.001054 | 212 |
| ENSP00000360683 | PTPN1 | 16 | 0.473 | 0.002109 | 347 |
| ENSP00000363998 | ITCH | 52 | 0.48 | 0.006854 | 619 |
| ENSP00000263686 | SELP | 27 | 0.482 | 0.003559 | 396 |
| ENSP00000266987 | TARBP2 | 1 | 0.485 | 0.000132 | 424 |
| ENSP00000249647 | SNAP23 | 27 | 0.493 | 0.003559 | 0 |
| ENSP00000300161 | YWHAB | 31 | 0.494 | 0.004086 | 927 |
| ENSP00000379330 | NFATC2 | 12 | 0.496 | 0.001582 | 227 |
| ENSP00000398597 | EXOSC6 | 68 | 0.498 | 0.008963 | 0 |
| ENSP00000356070 | MAPKAPK2 | 281 | 0.5 | 0.037037 | 998 |
| ENSP00000297518 | CDK5 | 53 | 0.501 | 0.006986 | 340 |
| ENSP00000309103 | BAD | 3 | 0.505 | 0.000395 | 281 |
| ENSP00000011653 | CD4 | 27 | 0.508 | 0.003559 | 752 |
| ENSP00000256452 | IL5RA | 26 | 0.513 | 0.003427 | 0 |
| ENSP00000264951 | XRN1 | 212 | 0.519 | 0.027943 | 191 |
| ENSP00000350003 | CCR3 | 25 | 0.521 | 0.003295 | 271 |
| ENSP00000227758 | BIRC2 | 27 | 0.522 | 0.003559 | 162 |
| ENSP00000308533 | GEMIN2 | 304 | 0.529 | 0.040069 | 229 |
| ENSP00000358716 | DDX20 | 304 | 0.529 | 0.040069 | 462 |
| ENSP00000296581 | LSM6 | 1 | 0.53 | 0.000132 | 0 |
| ENSP00000326031 | PPP1CA | 2 | 0.531 | 0.000264 | 186 |
| ENSP00000342374 | SNRPD2 | 27 | 0.531 | 0.003559 | 0 |
| ENSP00000242577 | DYNLL1 | 24 | 0.532 | 0.003163 | 200 |
| ENSP00000261461 | PPP2R5A | 28 | 0.533 | 0.003691 | 0 |
| ENSP00000219255 | PARD6A | 25 | 0.534 | 0.003295 | 927 |
| ENSP00000233057 | EIF2AK2 | 1 | 0.534 | 0.000132 | 768 |
| ENSP00000220592 | AGO2 | 304 | 0.537 | 0.040069 | 835 |
| ENSP00000268058 | PML | 11 | 0.538 | 0.00145 | 963 |
| ENSP00000281708 | FBXW7 | 3 | 0.539 | 0.000395 | 193 |
| ENSP00000338983 | MUC1 | 27 | 0.541 | 0.003559 | 299 |
| ENSP00000259808 | RIPK1 | 82 | 0.542 | 0.010808 | 165 |
| ENSP00000308450 | CDC20 | 39 | 0.543 | 0.00514 | 213 |
| ENSP00000385269 | ELAVL1 | 2 | 0.543 | 0.000264 | 347 |
| ENSP00000413035 | RBFOX2 | 2 | 0.547 | 0.000264 | 0 |
| ENSP00000287497 | ITGAM | 54 | 0.555 | 0.007117 | 306 |
| ENSP00000336790 | ATF4 | 1 | 0.559 | 0.000132 | 894 |
| ENSP00000326804 | CUL1 | 2 | 0.563 | 0.000264 | 306 |
| ENSP00000320940 | NCOA1 | 227 | 0.566 | 0.02992 | 996 |
| ENSP00000367408 | CASK | 19 | 0.569 | 0.002504 | 307 |
| ENSP00000306245 | FOS | 55 | 0.57 | 0.007249 | 565 |
| ENSP00000284957 | RABGEF1 | 23 | 0.571 | 0.003032 | 0 |
| ENSP00000262477 | RABEP1 | 23 | 0.572 | 0.003032 | 0 |
| ENSP00000339109 | ANAPC1 | 7 | 0.589 | 0.000923 | 0 |
| ENSP00000358997 | IRAK1 | 158 | 0.592 | 0.020825 | 278 |
| ENSP00000206249 | ESR1 | 786 | 0.593 | 0.103598 | 958 |
| ENSP00000249299 | NAA38 | 41 | 0.595 | 0.005404 | 0 |
| ENSP00000300413 | SNRPD1 | 304 | 0.595 | 0.040069 | 0 |
| ENSP00000382004 | CTNND1 | 6 | 0.601 | 0.000791 | 885 |
| ENSP00000256383 | EIF2S1 | 1 | 0.609 | 0.000132 | 245 |
| ENSP00000329357 | SP1 | 1 | 0.611 | 0.000132 | 922 |
| ENSP00000268712 | NCOR1 | 15 | 0.619 | 0.001977 | 995 |
| ENSP00000374455 | SQSTM1 | 18 | 0.619 | 0.002372 | 491 |
| ENSP00000355325 | PSMB5 | 27 | 0.624 | 0.003559 | 899 |
| ENSP00000227378 | HSPA8 | 50 | 0.629 | 0.00659 | 949 |
| ENSP00000293272 | CCL5 | 25 | 0.631 | 0.003295 | 219 |
| ENSP00000239223 | DUSP1 | 15 | 0.645 | 0.001977 | 344 |
| ENSP00000262633 | RBM42 | 2 | 0.645 | 0.000264 | 0 |
| ENSP00000311677 | PPP1R8 | 2 | 0.646 | 0.000264 | 0 |
| ENSP00000332468 | TRAF3 | 27 | 0.647 | 0.003559 | 666 |
| ENSP00000245960 | CDC25B | 10 | 0.65 | 0.001318 | 0 |
| ENSP00000300134 | STAT6 | 4 | 0.654 | 0.000527 | 900 |
| ENSP00000296585 | ITGA2 | 559 | 0.658 | 0.073679 | 987 |
| ENSP00000231487 | SKP1 | 56 | 0.664 | 0.007381 | 605 |
| ENSP00000314491 | SRRT | 50 | 0.667 | 0.00659 | 581 |
| ENSP00000304592 | FASN | 27 | 0.671 | 0.003559 | 609 |
| ENSP00000046794 | LCP2 | 37 | 0.686 | 0.004877 | 0 |
| ENSP00000324804 | PPP2R1A | 35 | 0.687 | 0.004613 | 258 |
| ENSP00000370473 | IGFBP3 | 77 | 0.692 | 0.010149 | 729 |
| ENSP00000384273 | RELA | 9 | 0.695 | 0.001186 | 800 |
| ENSP00000295897 | ALB | 174 | 0.709 | 0.022934 | 879 |
| ENSP00000348986 | INS-IGF2 | 62 | 0.715 | 0.008172 | 994 |
| ENSP00000302150 | PRL | 79 | 0.72 | 0.010413 | 842 |
| ENSP00000348554 | CDC16 | 7 | 0.724 | 0.000923 | 0 |
| ENSP00000357656 | FYN | 218 | 0.739 | 0.028733 | 371 |
| ENSP00000303830 | INSR | 55 | 0.745 | 0.007249 | 729 |
| ENSP00000378165 | ZNF207 | 37 | 0.748 | 0.004877 | 0 |
| ENSP00000292303 | CCR5 | 25 | 0.766 | 0.003295 | 427 |
| ENSP00000284811 | TCEB1 | 14 | 0.767 | 0.001845 | 0 |
| ENSP00000303939 | CTLA4 | 92 | 0.768 | 0.012126 | 330 |
| ENSP00000357858 | BUB3 | 37 | 0.768 | 0.004877 | 0 |
| ENSP00000371432 | PRLR | 76 | 0.776 | 0.010017 | 268 |
| ENSP00000341551 | SMAD4 | 282 | 0.777 | 0.037169 | 995 |
| ENSP00000264110 | ATF2 | 123 | 0.782 | 0.016212 | 579 |
| ENSP00000229022 | VDR | 104 | 0.785 | 0.013708 | 909 |
| ENSP00000366135 | EXOSC10 | 251 | 0.787 | 0.033083 | 270 |
| ENSP00000251849 | RAF1 | 233 | 0.793 | 0.03071 | 366 |
| ENSP00000339007 | GRB2 | 470 | 0.798 | 0.061948 | 374 |
| ENSP00000371067 | JAK2 | 403 | 0.799 | 0.053117 | 379 |
| ENSP00000240185 | TARDBP | 249 | 0.805 | 0.032819 | 228 |
| ENSP00000262238 | YY1 | 12 | 0.814 | 0.001582 | 943 |
| ENSP00000309845 | HRAS | 322 | 0.824 | 0.042441 | 467 |
| ENSP00000337825 | LCK | 51 | 0.83 | 0.006722 | 380 |
| ENSP00000263309 | CLNS1A | 39 | 0.833 | 0.00514 | 0 |
| ENSP00000319169 | PRMT5 | 39 | 0.834 | 0.00514 | 0 |
| ENSP00000302967 | HDAC3 | 29 | 0.845 | 0.003822 | 914 |
| ENSP00000252622 | LSM7 | 59 | 0.857 | 0.007776 | 0 |
| ENSP00000263967 | PIK3CA | 56 | 0.869 | 0.007381 | 500 |
| ENSP00000264033 | CBL | 1074 | 0.874 | 0.141558 | 249 |
| ENSP00000358022 | MCL1 | 44 | 0.875 | 0.005799 | 516 |
| ENSP00000361626 | YBX1 | 7 | 0.875 | 0.000923 | 860 |
| ENSP00000265171 | EGF | 61 | 0.879 | 0.00804 | 540 |
| ENSP00000363868 | ABCA1 | 7 | 0.884 | 0.000923 | 990 |
| ENSP00000215829 | SNRPD3 | 59 | 0.888 | 0.007776 | 0 |
| ENSP00000221494 | SF3A2 | 306 | 0.892 | 0.040332 | 0 |
| ENSP00000269321 | ARHGDIA | 15 | 0.9 | 0.001977 | 180 |
| ENSP00000335153 | HSP90AA1 | 277 | 0.903 | 0.03651 | 666 |
| ENSP00000349467 | CALM1 | 27 | 0.906 | 0.003559 | 563 |
| ENSP00000353483 | MAPK8 | 88 | 0.907 | 0.011599 | 858 |
| ENSP00000354394 | STAT1 | 73 | 0.911 | 0.009622 | 808 |
| ENSP00000307863 | U2AF2 | 290 | 0.913 | 0.038223 | 0 |
| ENSP00000350941 | SRC | 427 | 0.914 | 0.05628 | 856 |
| ENSP00000352980 | HIST1H4A | 5 | 0.925 | 0.000659 | 348 |
| ENSP00000235090 | WDR77 | 39 | 0.926 | 0.00514 | 0 |
| ENSP00000315702 | MOB4 | 24 | 0.947 | 0.003163 | 0 |
| ENSP00000233946 | IL1R1 | 27 | 0.954 | 0.003559 | 240 |
| ENSP00000379625 | MYD88 | 54 | 0.964 | 0.007117 | 235 |
| ENSP00000313829 | KHDRBS1 | 269 | 0.967 | 0.035455 | 0 |
| ENSP00000302269 | VAV1 | 3 | 0.978 | 0.000395 | 184 |

1. 390 candidate co-regeneration genes of dentine and vessel

| **Ensembl ID** | **Gene symbol** | **Betweenness** | **P-value** | **Betweenness ratio** | **Min-Max interaction score** |
| --- | --- | --- | --- | --- | --- |
| ENSP00000286332 | TAB2 | 26 | <0.001 | 0.003732 | 899 |
| ENSP00000360519 | RBP4 | 27 | <0.001 | 0.003876 | 265 |
| ENSP00000001008 | FKBP4 | 27 | 0.001 | 0.003876 | 240 |
| ENSP00000316854 | ATOX1 | 27 | 0.001 | 0.003876 | 0 |
| ENSP00000231572 | RARS | 258 | 0.003 | 0.037037 | 865 |
| ENSP00000295119 | NUP35 | 256 | 0.006 | 0.03675 | 0 |
| ENSP00000340944 | PTPN11 | 66 | 0.006 | 0.009475 | 281 |
| ENSP00000360483 | TMEM48 | 256 | 0.006 | 0.03675 | 228 |
| ENSP00000296181 | ITGB5 | 1 | 0.007 | 0.000144 | 825 |
| ENSP00000303864 | OR8I2 | 27 | 0.008 | 0.003876 | 0 |
| ENSP00000320709 | ADIPOQ | 27 | 0.009 | 0.003876 | 427 |
| ENSP00000327246 | VIPR1 | 24 | 0.009 | 0.003445 | 0 |
| ENSP00000350616 | DDC | 27 | 0.009 | 0.003876 | 306 |
| ENSP00000342905 | ADNP | 27 | 0.01 | 0.003876 | 0 |
| ENSP00000362649 | HDAC1 | 112 | 0.01 | 0.016078 | 993 |
| ENSP00000310668 | NUP93 | 256 | 0.012 | 0.03675 | 0 |
| ENSP00000313809 | AMBN | 258 | 0.012 | 0.037037 | 260 |
| ENSP00000365851 | BMI1 | 26 | 0.012 | 0.003732 | 229 |
| ENSP00000255390 | SCO1 | 258 | 0.013 | 0.037037 | 0 |
| ENSP00000299335 | COX11 | 258 | 0.013 | 0.037037 | 303 |
| ENSP00000370571 | TH | 27 | 0.014 | 0.003876 | 419 |
| ENSP00000269397 | CBX4 | 26 | 0.015 | 0.003732 | 185 |
| ENSP00000290921 | CTBP1 | 26 | 0.017 | 0.003732 | 867 |
| ENSP00000236147 | SELL | 27 | 0.018 | 0.003876 | 828 |
| ENSP00000309572 | TERT | 27 | 0.018 | 0.003876 | 346 |
| ENSP00000322142 | ING5 | 27 | 0.018 | 0.003876 | 179 |
| ENSP00000229390 | SRSF9 | 27 | 0.019 | 0.003876 | 0 |
| ENSP00000256458 | IRAK2 | 27 | 0.019 | 0.003876 | 0 |
| ENSP00000263269 | GRIN2D | 1 | 0.019 | 0.000144 | 0 |
| ENSP00000355245 | PAX9 | 27 | 0.019 | 0.003876 | 534 |
| ENSP00000358309 | EPHA7 | 27 | 0.02 | 0.003876 | 0 |
| ENSP00000260228 | MMP20 | 258 | 0.021 | 0.037037 | 167 |
| ENSP00000220751 | RIPK2 | 27 | 0.022 | 0.003876 | 204 |
| ENSP00000263464 | BIRC3 | 27 | 0.022 | 0.003876 | 0 |
| ENSP00000316042 | HNRNPA0 | 24 | 0.023 | 0.003445 | 0 |
| ENSP00000156825 | MBD3 | 27 | 0.024 | 0.003876 | 0 |
| ENSP00000262077 | NUP153 | 251 | 0.024 | 0.036032 | 456 |
| ENSP00000269886 | SH3GL1 | 26 | 0.024 | 0.003732 | 468 |
| ENSP00000317337 | CD300LB | 27 | 0.024 | 0.003876 | 0 |
| ENSP00000334003 | INTU | 27 | 0.024 | 0.003876 | 462 |
| ENSP00000376076 | SUMO1 | 21 | 0.024 | 0.003015 | 619 |
| ENSP00000387662 | GCG | 76 | 0.026 | 0.01091 | 896 |
| ENSP00000244520 | SNRPC | 25 | 0.027 | 0.003589 | 0 |
| ENSP00000262188 | SMARCD3 | 27 | 0.028 | 0.003876 | 900 |
| ENSP00000363868 | ABCA1 | 30 | 0.028 | 0.004307 | 954 |
| ENSP00000413720 | CDKN1C | 27 | 0.028 | 0.003876 | 260 |
| ENSP00000227507 | CCND1 | 263 | 0.03 | 0.037755 | 946 |
| ENSP00000223029 | AIMP2 | 283 | 0.031 | 0.040626 | 0 |
| ENSP00000364802 | HSPA1A | 27 | 0.031 | 0.003876 | 444 |
| ENSP00000318868 | SHMT1 | 27 | 0.032 | 0.003876 | 207 |
| ENSP00000260605 | DYNC2LI1 | 27 | 0.034 | 0.003876 | 0 |
| ENSP00000247225 | SGPP1 | 27 | 0.035 | 0.003876 | 0 |
| ENSP00000361162 | TOE1 | 2 | 0.035 | 0.000287 | 0 |
| ENSP00000298130 | SPTSSA | 27 | 0.036 | 0.003876 | 0 |
| ENSP00000377492 | HMMR | 27 | 0.036 | 0.003876 | 621 |
| ENSP00000332973 | SMAD3 | 490 | 0.038 | 0.070342 | 875 |
| ENSP00000344468 | SDC3 | 1 | 0.038 | 0.000144 | 800 |
| ENSP00000247843 | YEATS4 | 27 | 0.039 | 0.003876 | 0 |
| ENSP00000338127 | TESK1 | 26 | 0.039 | 0.003732 | 0 |
| ENSP00000338799 | IL6ST | 27 | 0.039 | 0.003876 | 900 |
| ENSP00000290158 | KPNB1 | 251 | 0.04 | 0.036032 | 0 |
| ENSP00000261507 | MSMO1 | 27 | 0.041 | 0.003876 | 0 |
| ENSP00000386741 | CHN1 | 27 | 0.041 | 0.003876 | 347 |
| ENSP00000332353 | PTCH1 | 131 | 0.044 | 0.018806 | 878 |
| ENSP00000372224 | HGFAC | 27 | 0.044 | 0.003876 | 0 |
| ENSP00000350369 | MAFG | 27 | 0.046 | 0.003876 | 0 |
| ENSP00000364000 | COL5A2 | 27 | 0.046 | 0.003876 | 985 |
| ENSP00000222399 | LAMB1 | 25 | 0.047 | 0.003589 | 396 |
| ENSP00000354360 | LAMC3 | 27 | 0.047 | 0.003876 | 0 |
| ENSP00000250003 | MYOD1 | 208 | 0.048 | 0.029859 | 848 |
| ENSP00000262554 | SPTLC1 | 27 | 0.048 | 0.003876 | 0 |
| ENSP00000285949 | CYP26C1 | 27 | 0.05 | 0.003876 | 0 |
| ENSP00000373340 | BRPF1 | 27 | 0.05 | 0.003876 | 268 |
| ENSP00000362166 | MEAF6 | 27 | 0.051 | 0.003876 | 0 |
| ENSP00000267163 | RB1 | 81 | 0.052 | 0.011628 | 653 |
| ENSP00000272190 | REN | 27 | 0.052 | 0.003876 | 430 |
| ENSP00000303212 | SEMA3E | 27 | 0.052 | 0.003876 | 0 |
| ENSP00000216181 | MYH9 | 27 | 0.053 | 0.003876 | 340 |
| ENSP00000251074 | NUP37 | 1 | 0.053 | 0.000144 | 213 |
| ENSP00000300403 | TPX2 | 24 | 0.053 | 0.003445 | 0 |
| ENSP00000216225 | RBX1 | 73 | 0.054 | 0.010479 | 193 |
| ENSP00000312697 | DMAP1 | 54 | 0.055 | 0.007752 | 233 |
| ENSP00000331831 | GAS6 | 3 | 0.059 | 0.000431 | 238 |
| ENSP00000295797 | PRKCI | 20 | 0.06 | 0.002871 | 193 |
| ENSP00000327048 | MAF | 27 | 0.062 | 0.003876 | 184 |
| ENSP00000353847 | WWTR1 | 27 | 0.062 | 0.003876 | 899 |
| ENSP00000361626 | YBX1 | 1 | 0.062 | 0.000144 | 860 |
| ENSP00000354621 | SMURF1 | 26 | 0.063 | 0.003732 | 995 |
| ENSP00000245919 | FOSB | 17 | 0.066 | 0.00244 | 339 |
| ENSP00000229769 | FANCE | 27 | 0.067 | 0.003876 | 0 |
| ENSP00000240922 | NAA50 | 27 | 0.067 | 0.003876 | 335 |
| ENSP00000351908 | MAP3K5 | 1 | 0.068 | 0.000144 | 219 |
| ENSP00000334008 | PARVA | 26 | 0.069 | 0.003732 | 0 |
| ENSP00000281928 | MED13L | 27 | 0.07 | 0.003876 | 899 |
| ENSP00000302955 | RRM2 | 1 | 0.07 | 0.000144 | 182 |
| ENSP00000204604 | CHRD | 27 | 0.072 | 0.003876 | 524 |
| ENSP00000338477 | HNRNPF | 24 | 0.072 | 0.003445 | 195 |
| ENSP00000220584 | FDFT1 | 27 | 0.074 | 0.003876 | 899 |
| ENSP00000339428 | SOCS2 | 12 | 0.074 | 0.001723 | 0 |
| ENSP00000315644 | TYMS | 27 | 0.076 | 0.003876 | 427 |
| ENSP00000355778 | H3F3A | 27 | 0.082 | 0.003876 | 558 |
| ENSP00000360672 | PARD6B | 7 | 0.082 | 0.001005 | 619 |
| ENSP00000254719 | RPA1 | 22 | 0.083 | 0.003158 | 234 |
| ENSP00000314458 | CDC42 | 11 | 0.083 | 0.001579 | 467 |
| ENSP00000340330 | KAT5 | 54 | 0.084 | 0.007752 | 228 |
| ENSP00000231449 | IL4 | 27 | 0.085 | 0.003876 | 800 |
| ENSP00000348965 | DYNC1H1 | 27 | 0.086 | 0.003876 | 223 |
| ENSP00000223023 | WASL | 11 | 0.087 | 0.001579 | 0 |
| ENSP00000373614 | SELPLG | 27 | 0.087 | 0.003876 | 340 |
| ENSP00000247668 | TRAF2 | 298 | 0.089 | 0.042779 | 654 |
| ENSP00000375863 | HNRNPUL1 | 27 | 0.089 | 0.003876 | 0 |
| ENSP00000293379 | ITGA5 | 608 | 0.091 | 0.087281 | 936 |
| ENSP00000356070 | MAPKAPK2 | 258 | 0.092 | 0.037037 | 993 |
| ENSP00000356213 | VIP | 27 | 0.092 | 0.003876 | 307 |
| ENSP00000218388 | TIMP1 | 258 | 0.094 | 0.037037 | 505 |
| ENSP00000252444 | LDLR | 8 | 0.094 | 0.001148 | 469 |
| ENSP00000300134 | STAT6 | 27 | 0.094 | 0.003876 | 900 |
| ENSP00000396127 | RAN | 275 | 0.094 | 0.039477 | 899 |
| ENSP00000348577 | RANGAP1 | 275 | 0.096 | 0.039477 | 0 |
| ENSP00000259089 | BLK | 26 | 0.097 | 0.003732 | 227 |
| ENSP00000318297 | RUVBL1 | 54 | 0.097 | 0.007752 | 930 |
| ENSP00000263208 | HIRA | 27 | 0.104 | 0.003876 | 714 |
| ENSP00000361423 | ABL1 | 9 | 0.105 | 0.001292 | 319 |
| ENSP00000328777 | EFNA5 | 27 | 0.108 | 0.003876 | 201 |
| ENSP00000346389 | MEF2A | 5 | 0.109 | 0.000718 | 247 |
| ENSP00000402240 | KIAA1432 | 27 | 0.109 | 0.003876 | 0 |
| ENSP00000285968 | NUP205 | 5 | 0.11 | 0.000718 | 0 |
| ENSP00000257904 | CDK4 | 31 | 0.111 | 0.00445 | 378 |
| ENSP00000361066 | NCOA3 | 56 | 0.113 | 0.008039 | 912 |
| ENSP00000309913 | TBX5 | 27 | 0.114 | 0.003876 | 949 |
| ENSP00000279146 | AIP | 27 | 0.115 | 0.003876 | 631 |
| ENSP00000282588 | ITGA1 | 2 | 0.115 | 0.000287 | 912 |
| ENSP00000322788 | MMP1 | 4 | 0.116 | 0.000574 | 414 |
| ENSP00000384442 | CDK11A | 27 | 0.119 | 0.003876 | 151 |
| ENSP00000362441 | ATRX | 27 | 0.121 | 0.003876 | 0 |
| ENSP00000335544 | CCKBR | 25 | 0.122 | 0.003589 | 0 |
| ENSP00000335657 | CCK | 27 | 0.122 | 0.003876 | 329 |
| ENSP00000287647 | FANCD2 | 27 | 0.123 | 0.003876 | 0 |
| ENSP00000239938 | EGR1 | 27 | 0.125 | 0.003876 | 273 |
| ENSP00000327758 | NKX2-5 | 27 | 0.128 | 0.003876 | 491 |
| ENSP00000266000 | DAXX | 27 | 0.13 | 0.003876 | 676 |
| ENSP00000400365 | LAMA2 | 52 | 0.13 | 0.007465 | 260 |
| ENSP00000256442 | CCNB1 | 194 | 0.132 | 0.02785 | 749 |
| ENSP00000264335 | YWHAE | 27 | 0.132 | 0.003876 | 925 |
| ENSP00000295897 | ALB | 82 | 0.133 | 0.011771 | 879 |
| ENSP00000296543 | NAA15 | 5 | 0.133 | 0.000718 | 0 |
| ENSP00000400717 | GNA13 | 27 | 0.133 | 0.003876 | 213 |
| ENSP00000230882 | GHR | 258 | 0.134 | 0.037037 | 675 |
| ENSP00000351905 | TGFBR2 | 27 | 0.134 | 0.003876 | 999 |
| ENSP00000246071 | SNRPB2 | 30 | 0.135 | 0.004307 | 0 |
| ENSP00000256474 | VHL | 743 | 0.135 | 0.106661 | 165 |
| ENSP00000380252 | NFE2L2 | 27 | 0.136 | 0.003876 | 317 |
| ENSP00000343619 | HOXA9 | 27 | 0.138 | 0.003876 | 173 |
| ENSP00000265564 | EXOSC7 | 1 | 0.142 | 0.000144 | 0 |
| ENSP00000268296 | ITGAX | 2 | 0.145 | 0.000287 | 180 |
| ENSP00000254066 | RARA | 7 | 0.147 | 0.001005 | 905 |
| ENSP00000346294 | S100A4 | 27 | 0.149 | 0.003876 | 277 |
| ENSP00000255764 | MED10 | 27 | 0.151 | 0.003876 | 899 |
| ENSP00000262965 | TCF3 | 54 | 0.151 | 0.007752 | 340 |
| ENSP00000380942 | ARHGEF12 | 27 | 0.152 | 0.003876 | 0 |
| ENSP00000300093 | PLK1 | 54 | 0.153 | 0.007752 | 305 |
| ENSP00000364336 | TBXA2R | 27 | 0.154 | 0.003876 | 0 |
| ENSP00000367207 | MYC | 17 | 0.154 | 0.00244 | 851 |
| ENSP00000323050 | RBBP8 | 13 | 0.158 | 0.001866 | 228 |
| ENSP00000232014 | BCL6 | 258 | 0.159 | 0.037037 | 952 |
| ENSP00000296785 | ANKRA2 | 2 | 0.159 | 0.000287 | 0 |
| ENSP00000363435 | ITPR3 | 2 | 0.16 | 0.000287 | 919 |
| ENSP00000290663 | MED8 | 27 | 0.167 | 0.003876 | 899 |
| ENSP00000363071 | DES | 2 | 0.168 | 0.000287 | 532 |
| ENSP00000386259 | NEB | 2 | 0.168 | 0.000287 | 207 |
| ENSP00000360683 | PTPN1 | 5 | 0.169 | 0.000718 | 347 |
| ENSP00000261908 | NEO1 | 30 | 0.171 | 0.004307 | 800 |
| ENSP00000360247 | CYP2J2 | 27 | 0.171 | 0.003876 | 507 |
| ENSP00000229179 | NUP107 | 5 | 0.173 | 0.000718 | 0 |
| ENSP00000254480 | SMARCC1 | 27 | 0.173 | 0.003876 | 296 |
| ENSP00000320940 | NCOA1 | 189 | 0.173 | 0.027132 | 979 |
| ENSP00000278385 | CD44 | 57 | 0.178 | 0.008183 | 936 |
| ENSP00000222005 | CDC37 | 5 | 0.18 | 0.000718 | 0 |
| ENSP00000352798 | COL18A1 | 25 | 0.182 | 0.003589 | 911 |
| ENSP00000312435 | DAG1 | 52 | 0.183 | 0.007465 | 241 |
| ENSP00000316054 | DVL3 | 27 | 0.189 | 0.003876 | 288 |
| ENSP00000292644 | PSMC2 | 32 | 0.19 | 0.004594 | 415 |
| ENSP00000231487 | SKP1 | 91 | 0.194 | 0.013063 | 605 |
| ENSP00000262435 | SMURF2 | 5 | 0.198 | 0.000718 | 998 |
| ENSP00000331358 | GAST | 25 | 0.198 | 0.003589 | 368 |
| ENSP00000324897 | UBE2I | 254 | 0.199 | 0.036463 | 644 |
| ENSP00000264110 | ATF2 | 100 | 0.201 | 0.014355 | 579 |
| ENSP00000265023 | KNG1 | 27 | 0.204 | 0.003876 | 379 |
| ENSP00000268058 | PML | 29 | 0.204 | 0.004163 | 963 |
| ENSP00000383623 | MLLT4 | 258 | 0.204 | 0.037037 | 899 |
| ENSP00000258962 | SRSF1 | 2 | 0.208 | 0.000287 | 319 |
| ENSP00000352712 | DACH1 | 54 | 0.21 | 0.007752 | 190 |
| ENSP00000247161 | ELK1 | 6 | 0.211 | 0.000861 | 0 |
| ENSP00000229595 | ASF1A | 27 | 0.212 | 0.003876 | 266 |
| ENSP00000313950 | AURKB | 2 | 0.217 | 0.000287 | 243 |
| ENSP00000389140 | DCC | 48 | 0.217 | 0.006891 | 235 |
| ENSP00000371067 | JAK2 | 304 | 0.222 | 0.043641 | 379 |
| ENSP00000295926 | CCNL1 | 25 | 0.226 | 0.003589 | 0 |
| ENSP00000337014 | HFE2 | 6 | 0.226 | 0.000861 | 260 |
| ENSP00000339151 | IKBKB | 5 | 0.227 | 0.000718 | 749 |
| ENSP00000417763 | NAA10 | 5 | 0.228 | 0.000718 | 0 |
| ENSP00000354791 | DCTN1 | 27 | 0.231 | 0.003876 | 229 |
| ENSP00000362820 | SRSF3 | 4 | 0.234 | 0.000574 | 0 |
| ENSP00000377141 | ARRB1 | 27 | 0.238 | 0.003876 | 201 |
| ENSP00000350877 | SRSF2 | 4 | 0.24 | 0.000574 | 0 |
| ENSP00000348108 | KHDRBS3 | 25 | 0.241 | 0.003589 | 430 |
| ENSP00000276420 | DOK2 | 30 | 0.243 | 0.004307 | 0 |
| ENSP00000245907 | C3 | 27 | 0.247 | 0.003876 | 369 |
| ENSP00000351997 | MAP2K6 | 128 | 0.247 | 0.018375 | 167 |
| ENSP00000344115 | CDH5 | 41 | 0.25 | 0.005886 | 346 |
| ENSP00000366466 | CTNNBIP1 | 25 | 0.254 | 0.003589 | 0 |
| ENSP00000350283 | BRCA1 | 31 | 0.257 | 0.00445 | 676 |
| ENSP00000317272 | MET | 27 | 0.26 | 0.003876 | 842 |
| ENSP00000363998 | ITCH | 26 | 0.263 | 0.003732 | 619 |
| ENSP00000361850 | PLAU | 3 | 0.267 | 0.000431 | 380 |
| ENSP00000228682 | GLI1 | 25 | 0.268 | 0.003589 | 412 |
| ENSP00000348786 | RAP1A | 54 | 0.268 | 0.007752 | 177 |
| ENSP00000239223 | DUSP1 | 14 | 0.27 | 0.00201 | 344 |
| ENSP00000260130 | SDCBP | 26 | 0.272 | 0.003732 | 200 |
| ENSP00000287934 | FZD1 | 284 | 0.285 | 0.040769 | 896 |
| ENSP00000371973 | SAP18 | 2 | 0.285 | 0.000287 | 174 |
| ENSP00000351665 | CLIP1 | 27 | 0.288 | 0.003876 | 333 |
| ENSP00000334122 | FGF3 | 27 | 0.289 | 0.003876 | 906 |
| ENSP00000363763 | EPHB2 | 2 | 0.289 | 0.000287 | 200 |
| ENSP00000299293 | FRS2 | 2 | 0.291 | 0.000287 | 367 |
| ENSP00000371532 | VLDLR | 3 | 0.292 | 0.000431 | 205 |
| ENSP00000281708 | FBXW7 | 12 | 0.294 | 0.001723 | 193 |
| ENSP00000226218 | SEBOX | 27 | 0.297 | 0.003876 | 499 |
| ENSP00000391349 | DOM3Z | 2 | 0.303 | 0.000287 | 193 |
| ENSP00000265056 | MCM2 | 25 | 0.311 | 0.003589 | 0 |
| ENSP00000334458 | GATA4 | 54 | 0.311 | 0.007752 | 416 |
| ENSP00000354876 | MT-CO2 | 258 | 0.312 | 0.037037 | 344 |
| ENSP00000264554 | SHC2 | 254 | 0.315 | 0.036463 | 260 |
| ENSP00000263686 | SELP | 54 | 0.317 | 0.007752 | 396 |
| ENSP00000368438 | PCNA | 26 | 0.325 | 0.003732 | 565 |
| ENSP00000312122 | SEC13 | 27 | 0.329 | 0.003876 | 0 |
| ENSP00000278616 | ATM | 11 | 0.331 | 0.001579 | 659 |
| ENSP00000262629 | TYROBP | 27 | 0.334 | 0.003876 | 254 |
| ENSP00000264606 | HDAC4 | 15 | 0.336 | 0.002153 | 685 |
| ENSP00000265171 | EGF | 21 | 0.336 | 0.003015 | 540 |
| ENSP00000338868 | PHF8 | 1 | 0.337 | 0.000144 | 345 |
| ENSP00000296585 | ITGA2 | 606 | 0.339 | 0.086994 | 987 |
| ENSP00000223129 | RPA3 | 22 | 0.341 | 0.003158 | 0 |
| ENSP00000316032 | NUP98 | 26 | 0.343 | 0.003732 | 362 |
| ENSP00000294172 | NXF1 | 30 | 0.346 | 0.004307 | 258 |
| ENSP00000251968 | TSG101 | 19 | 0.347 | 0.002728 | 0 |
| ENSP00000373952 | FANCA | 27 | 0.347 | 0.003876 | 0 |
| ENSP00000276201 | UPF3B | 4 | 0.352 | 0.000574 | 0 |
| ENSP00000222254 | PIK3R2 | 2 | 0.356 | 0.000287 | 619 |
| ENSP00000349467 | CALM1 | 26 | 0.361 | 0.003732 | 563 |
| ENSP00000381331 | HDAC2 | 43 | 0.367 | 0.006173 | 229 |
| ENSP00000306245 | FOS | 357 | 0.369 | 0.051249 | 565 |
| ENSP00000374455 | SQSTM1 | 20 | 0.37 | 0.002871 | 491 |
| ENSP00000287497 | ITGAM | 54 | 0.371 | 0.007752 | 306 |
| ENSP00000419692 | RXRA | 133 | 0.371 | 0.019093 | 997 |
| ENSP00000405934 | ITPR1 | 25 | 0.372 | 0.003589 | 502 |
| ENSP00000304895 | IRS1 | 40 | 0.374 | 0.005742 | 859 |
| ENSP00000219476 | TSC2 | 3 | 0.381 | 0.000431 | 953 |
| ENSP00000292303 | CCR5 | 7 | 0.383 | 0.001005 | 427 |
| ENSP00000261769 | CDH1 | 49 | 0.394 | 0.007034 | 700 |
| ENSP00000370938 | CDK8 | 10 | 0.395 | 0.001436 | 899 |
| ENSP00000225831 | CCL2 | 26 | 0.397 | 0.003732 | 879 |
| ENSP00000162749 | TNFRSF1A | 276 | 0.398 | 0.039621 | 347 |
| ENSP00000360286 | RAE1 | 26 | 0.4 | 0.003732 | 218 |
| ENSP00000356024 | CR2 | 25 | 0.407 | 0.003589 | 307 |
| ENSP00000046794 | LCP2 | 8 | 0.408 | 0.001148 | 0 |
| ENSP00000297518 | CDK5 | 27 | 0.409 | 0.003876 | 340 |
| ENSP00000256452 | IL5RA | 26 | 0.411 | 0.003732 | 0 |
| ENSP00000229854 | MCM3 | 3 | 0.412 | 0.000431 | 0 |
| ENSP00000352980 | HIST1H4A | 12 | 0.417 | 0.001723 | 348 |
| ENSP00000248572 | GNGT1 | 27 | 0.426 | 0.003876 | 0 |
| ENSP00000162330 | BCAR1 | 43 | 0.438 | 0.006173 | 229 |
| ENSP00000223095 | SERPINE1 | 26 | 0.439 | 0.003732 | 833 |
| ENSP00000345008 | FBLN5 | 27 | 0.44 | 0.003876 | 401 |
| ENSP00000314949 | POLR2A | 2 | 0.448 | 0.000287 | 195 |
| ENSP00000266970 | CDK2 | 31 | 0.46 | 0.00445 | 988 |
| ENSP00000282441 | YAP1 | 27 | 0.463 | 0.003876 | 938 |
| ENSP00000309103 | BAD | 1 | 0.465 | 0.000144 | 281 |
| ENSP00000378529 | FZR1 | 6 | 0.468 | 0.000861 | 0 |
| ENSP00000302564 | BCL2L1 | 1 | 0.47 | 0.000144 | 196 |
| ENSP00000313419 | CD19 | 25 | 0.474 | 0.003589 | 562 |
| ENSP00000364893 | ARHGEF7 | 17 | 0.475 | 0.00244 | 213 |
| ENSP00000170630 | IL4R | 27 | 0.476 | 0.003876 | 249 |
| ENSP00000341551 | SMAD4 | 333 | 0.478 | 0.047804 | 995 |
| ENSP00000354476 | SREBF2 | 54 | 0.48 | 0.007752 | 947 |
| ENSP00000206249 | ESR1 | 831 | 0.492 | 0.119294 | 958 |
| ENSP00000326031 | PPP1CA | 2 | 0.497 | 0.000287 | 186 |
| ENSP00000401980 | MAVS | 27 | 0.499 | 0.003876 | 0 |
| ENSP00000360798 | EPS15 | 27 | 0.502 | 0.003876 | 200 |
| ENSP00000367316 | ITGA8 | 56 | 0.512 | 0.008039 | 800 |
| ENSP00000248566 | SHFM1 | 32 | 0.513 | 0.004594 | 260 |
| ENSP00000303242 | ITGB2 | 56 | 0.514 | 0.008039 | 340 |
| ENSP00000311677 | PPP1R8 | 2 | 0.52 | 0.000287 | 0 |
| ENSP00000228837 | FGF6 | 306 | 0.522 | 0.043928 | 908 |
| ENSP00000302961 | HSPA4 | 31 | 0.525 | 0.00445 | 621 |
| ENSP00000216911 | AURKA | 24 | 0.526 | 0.003445 | 621 |
| ENSP00000348551 | NCOR2 | 259 | 0.527 | 0.037181 | 986 |
| ENSP00000353483 | MAPK8 | 42 | 0.527 | 0.006029 | 858 |
| ENSP00000352400 | NUP214 | 24 | 0.536 | 0.003445 | 210 |
| ENSP00000287598 | BUB1B | 11 | 0.538 | 0.001579 | 0 |
| ENSP00000262320 | AXIN1 | 359 | 0.553 | 0.051536 | 902 |
| ENSP00000340820 | MAPT | 27 | 0.561 | 0.003876 | 867 |
| ENSP00000398698 | TNF | 277 | 0.569 | 0.039765 | 942 |
| ENSP00000222256 | RAB3A | 27 | 0.57 | 0.003876 | 171 |
| ENSP00000307046 | SDC2 | 53 | 0.572 | 0.007608 | 805 |
| ENSP00000343274 | INTS8 | 32 | 0.578 | 0.004594 | 163 |
| ENSP00000337088 | MEN1 | 27 | 0.582 | 0.003876 | 401 |
| ENSP00000052754 | DCN | 4 | 0.583 | 0.000574 | 930 |
| ENSP00000252818 | JUND | 27 | 0.592 | 0.003876 | 867 |
| ENSP00000308450 | CDC20 | 15 | 0.601 | 0.002153 | 213 |
| ENSP00000269321 | ARHGDIA | 5 | 0.605 | 0.000718 | 180 |
| ENSP00000339007 | GRB2 | 223 | 0.607 | 0.032013 | 374 |
| ENSP00000261205 | SYT1 | 27 | 0.625 | 0.003876 | 369 |
| ENSP00000302269 | VAV1 | 28 | 0.628 | 0.00402 | 184 |
| ENSP00000310596 | LSM1 | 1 | 0.632 | 0.000144 | 210 |
| ENSP00000332468 | TRAF3 | 27 | 0.637 | 0.003876 | 666 |
| ENSP00000384675 | SOS1 | 38 | 0.637 | 0.005455 | 299 |
| ENSP00000256857 | GRP | 5 | 0.64 | 0.000718 | 290 |
| ENSP00000329357 | SP1 | 62 | 0.646 | 0.0089 | 922 |
| ENSP00000296871 | CSF2 | 9 | 0.652 | 0.001292 | 561 |
| ENSP00000384053 | CSF2RB | 9 | 0.652 | 0.001292 | 0 |
| ENSP00000219548 | STUB1 | 44 | 0.653 | 0.006316 | 621 |
| ENSP00000350275 | HIST1H3A | 28 | 0.658 | 0.00402 | 646 |
| ENSP00000339109 | ANAPC1 | 8 | 0.661 | 0.001148 | 0 |
| ENSP00000300161 | YWHAB | 3 | 0.664 | 0.000431 | 927 |
| ENSP00000382004 | CTNND1 | 10 | 0.668 | 0.001436 | 885 |
| ENSP00000344818 | UBC | 3458 | 0.669 | 0.496411 | 999 |
| ENSP00000011653 | CD4 | 8 | 0.671 | 0.001148 | 752 |
| ENSP00000307387 | PDCD6IP | 26 | 0.682 | 0.003732 | 0 |
| ENSP00000291552 | U2AF1 | 4 | 0.695 | 0.000574 | 0 |
| ENSP00000354554 | MT-CYB | 258 | 0.707 | 0.037037 | 344 |
| ENSP00000335153 | HSP90AA1 | 325 | 0.713 | 0.046655 | 666 |
| ENSP00000354394 | STAT1 | 40 | 0.715 | 0.005742 | 808 |
| ENSP00000220592 | AGO2 | 258 | 0.716 | 0.037037 | 302 |
| ENSP00000370473 | IGFBP3 | 66 | 0.718 | 0.009475 | 729 |
| ENSP00000398597 | EXOSC6 | 41 | 0.718 | 0.005886 | 0 |
| ENSP00000348554 | CDC16 | 6 | 0.719 | 0.000861 | 0 |
| ENSP00000262238 | YY1 | 4 | 0.727 | 0.000574 | 943 |
| ENSP00000299543 | CTDP1 | 44 | 0.728 | 0.006316 | 163 |
| ENSP00000351486 | NTRK1 | 99 | 0.741 | 0.014212 | 0 |
| ENSP00000314491 | SRRT | 44 | 0.747 | 0.006316 | 196 |
| ENSP00000245960 | CDC25B | 4 | 0.75 | 0.000574 | 0 |
| ENSP00000354961 | MT-ND4 | 258 | 0.754 | 0.037037 | 229 |
| ENSP00000348708 | UPF2 | 4 | 0.758 | 0.000574 | 0 |
| ENSP00000358716 | DDX20 | 258 | 0.76 | 0.037037 | 293 |
| ENSP00000309503 | YWHAZ | 3 | 0.768 | 0.000431 | 969 |
| ENSP00000215829 | SNRPD3 | 37 | 0.771 | 0.005312 | 0 |
| ENSP00000308533 | GEMIN2 | 258 | 0.786 | 0.037037 | 229 |
| ENSP00000302967 | HDAC3 | 17 | 0.788 | 0.00244 | 914 |
| ENSP00000300651 | MED1 | 25 | 0.79 | 0.003589 | 765 |
| ENSP00000284811 | TCEB1 | 12 | 0.809 | 0.001723 | 0 |
| ENSP00000338934 | EZR | 89 | 0.811 | 0.012776 | 430 |
| ENSP00000363822 | AR | 1 | 0.818 | 0.000144 | 978 |
| ENSP00000262613 | SLC9A3R1 | 342 | 0.82 | 0.049096 | 892 |
| ENSP00000369050 | CYP1A1 | 54 | 0.832 | 0.007752 | 922 |
| ENSP00000249299 | NAA38 | 51 | 0.836 | 0.007321 | 0 |
| ENSP00000300413 | SNRPD1 | 258 | 0.844 | 0.037037 | 0 |
| ENSP00000380921 | SH3KBP1 | 7 | 0.845 | 0.001005 | 0 |
| ENSP00000315859 | RNPS1 | 4 | 0.856 | 0.000574 | 0 |
| ENSP00000342374 | SNRPD2 | 18 | 0.859 | 0.002584 | 0 |
| ENSP00000235090 | WDR77 | 27 | 0.864 | 0.003876 | 0 |
| ENSP00000351407 | ARNT | 125 | 0.87 | 0.017944 | 0 |
| ENSP00000348827 | THRB | 3 | 0.874 | 0.000431 | 907 |
| ENSP00000378165 | ZNF207 | 25 | 0.88 | 0.003589 | 0 |
| ENSP00000357858 | BUB3 | 25 | 0.887 | 0.003589 | 0 |
| ENSP00000384273 | RELA | 7 | 0.899 | 0.001005 | 800 |
| ENSP00000227378 | HSPA8 | 44 | 0.905 | 0.006316 | 845 |
| ENSP00000300574 | CRK | 101 | 0.906 | 0.014499 | 307 |
| ENSP00000252102 | NDUFA2 | 258 | 0.909 | 0.037037 | 200 |
| ENSP00000263309 | CLNS1A | 29 | 0.909 | 0.004163 | 0 |
| ENSP00000319169 | PRMT5 | 29 | 0.91 | 0.004163 | 0 |
| ENSP00000264951 | XRN1 | 217 | 0.915 | 0.031151 | 263 |
| ENSP00000401303 | SHC1 | 96 | 0.918 | 0.013781 | 924 |
| ENSP00000264033 | CBL | 782 | 0.923 | 0.11226 | 249 |
| ENSP00000337825 | LCK | 25 | 0.927 | 0.003589 | 380 |
| ENSP00000268712 | NCOR1 | 4 | 0.931 | 0.000574 | 943 |
| ENSP00000358022 | MCL1 | 32 | 0.935 | 0.004594 | 516 |
| ENSP00000252622 | LSM7 | 37 | 0.939 | 0.005312 | 0 |
| ENSP00000309845 | HRAS | 304 | 0.944 | 0.043641 | 467 |
| ENSP00000222812 | STX1A | 27 | 0.945 | 0.003876 | 241 |
| ENSP00000263967 | PIK3CA | 39 | 0.967 | 0.005599 | 500 |
| ENSP00000251849 | RAF1 | 263 | 0.982 | 0.037755 | 366 |
| ENSP00000350941 | SRC | 478 | 0.983 | 0.068619 | 856 |
| ENSP00000229022 | VDR | 84 | 0.986 | 0.012059 | 909 |
| ENSP00000221494 | SF3A2 | 241 | 0.991 | 0.034597 | 0 |
| ENSP00000313829 | KHDRBS1 | 260 | 0.992 | 0.037324 | 0 |
| ENSP00000366135 | EXOSC10 | 264 | 0.995 | 0.037898 | 172 |
| ENSP00000240185 | TARDBP | 263 | 0.997 | 0.037755 | 228 |
| ENSP00000307863 | U2AF2 | 256 | 0.998 | 0.03675 | 0 |
| ENSP00000003084 | CFTR | 363 | 0.999 | 0.05211 | 429 |

1. 649 candidate co-regeneration genes of nerve and vessel

| **Ensembl ID** | **Gene symbol** | **Betweenness** | **P-value** | **Betweenness ratio** | **Min-Max interaction score** |
| --- | --- | --- | --- | --- | --- |
| ENSP00000220751 | RIPK2 | 281 | <0.001 | 0.003876 | 969 |
| ENSP00000001008 | FKBP4 | 281 | 0.001 | 0.003876 | 768 |
| ENSP00000316854 | ATOX1 | 281 | 0.001 | 0.003876 | 218 |
| ENSP00000360519 | RBP4 | 281 | 0.001 | 0.003876 | 920 |
| ENSP00000364864 | KIF3B | 3 | 0.001 | 4.14E-05 | 600 |
| ENSP00000321239 | RCHY1 | 258 | 0.002 | 0.003559 | 942 |
| ENSP00000330237 | CASP9 | 7 | 0.003 | 9.66E-05 | 996 |
| ENSP00000309572 | TERT | 281 | 0.004 | 0.003876 | 999 |
| ENSP00000314444 | WDR35 | 2 | 0.004 | 2.76E-05 | 428 |
| ENSP00000250003 | MYOD1 | 2465 | 0.005 | 0.034001 | 999 |
| ENSP00000342656 | EXT2 | 1 | 0.005 | 1.38E-05 | 995 |
| ENSP00000327647 | CRADD | 258 | 0.007 | 0.003559 | 306 |
| ENSP00000303864 | OR8I2 | 281 | 0.008 | 0.003876 | 0 |
| ENSP00000300935 | RAB8A | 3 | 0.01 | 4.14E-05 | 424 |
| ENSP00000340944 | PTPN11 | 1350 | 0.01 | 0.018621 | 999 |
| ENSP00000342905 | ADNP | 281 | 0.01 | 0.003876 | 428 |
| ENSP00000355651 | RAB4A | 3 | 0.01 | 4.14E-05 | 619 |
| ENSP00000262188 | SMARCD3 | 281 | 0.012 | 0.003876 | 917 |
| ENSP00000347858 | XIAP | 9 | 0.012 | 0.000124 | 906 |
| ENSP00000357711 | S100A7 | 258 | 0.012 | 0.003559 | 821 |
| ENSP00000376177 | CALCRL | 257 | 0.012 | 0.003545 | 326 |
| ENSP00000350616 | DDC | 281 | 0.014 | 0.003876 | 379 |
| ENSP00000351163 | COL11A1 | 3 | 0.014 | 4.14E-05 | 900 |
| ENSP00000323194 | PLXNA4 | 258 | 0.015 | 0.003559 | 887 |
| ENSP00000296695 | SPINK1 | 258 | 0.016 | 0.003559 | 621 |
| ENSP00000329384 | IL22 | 258 | 0.016 | 0.003559 | 369 |
| ENSP00000355001 | POU3F3 | 258 | 0.016 | 0.003559 | 466 |
| ENSP00000346901 | FMO1 | 1 | 0.017 | 1.38E-05 | 462 |
| ENSP00000387286 | RAB1A | 258 | 0.017 | 0.003559 | 717 |
| ENSP00000256458 | IRAK2 | 281 | 0.019 | 0.003876 | 916 |
| ENSP00000229390 | SRSF9 | 281 | 0.02 | 0.003876 | 936 |
| ENSP00000261207 | PPP1R12A | 257 | 0.02 | 0.003545 | 965 |
| ENSP00000384169 | FBLN2 | 1 | 0.02 | 1.38E-05 | 847 |
| ENSP00000322142 | ING5 | 281 | 0.021 | 0.003876 | 900 |
| ENSP00000375863 | HNRNPUL1 | 281 | 0.021 | 0.003876 | 344 |
| ENSP00000227507 | CCND1 | 3568 | 0.022 | 0.049215 | 991 |
| ENSP00000358081 | BAG3 | 258 | 0.022 | 0.003559 | 800 |
| ENSP00000370571 | TH | 281 | 0.023 | 0.003876 | 888 |
| ENSP00000156825 | MBD3 | 281 | 0.024 | 0.003876 | 723 |
| ENSP00000263464 | BIRC3 | 275 | 0.024 | 0.003793 | 831 |
| ENSP00000281537 | TJP1 | 2 | 0.025 | 2.76E-05 | 948 |
| ENSP00000283228 | PTPRR | 258 | 0.025 | 0.003559 | 995 |
| ENSP00000316845 | ARHGEF4 | 258 | 0.027 | 0.003559 | 462 |
| ENSP00000295731 | IHH | 20 | 0.029 | 0.000276 | 838 |
| ENSP00000327246 | VIPR1 | 261 | 0.03 | 0.0036 | 899 |
| ENSP00000384179 | ZFPM2 | 2 | 0.03 | 2.76E-05 | 505 |
| ENSP00000354280 | PRSS3 | 258 | 0.031 | 0.003559 | 837 |
| ENSP00000371067 | JAK2 | 2332 | 0.031 | 0.032166 | 999 |
| ENSP00000266085 | TIMP3 | 258 | 0.033 | 0.003559 | 835 |
| ENSP00000364000 | COL5A2 | 282 | 0.033 | 0.00389 | 915 |
| ENSP00000316042 | HNRNPA0 | 260 | 0.035 | 0.003586 | 171 |
| ENSP00000222399 | LAMB1 | 273 | 0.036 | 0.003766 | 918 |
| ENSP00000334003 | INTU | 281 | 0.036 | 0.003876 | 659 |
| ENSP00000386741 | CHN1 | 281 | 0.036 | 0.003876 | 778 |
| ENSP00000394794 | PTPN13 | 256 | 0.036 | 0.003531 | 747 |
| ENSP00000413720 | CDKN1C | 281 | 0.036 | 0.003876 | 930 |
| ENSP00000286332 | TAB2 | 267 | 0.037 | 0.003683 | 966 |
| ENSP00000264708 | POMC | 10 | 0.038 | 0.000138 | 921 |
| ENSP00000250894 | MAPK8IP3 | 258 | 0.039 | 0.003559 | 892 |
| ENSP00000260762 | EXOC6 | 2 | 0.039 | 2.76E-05 | 651 |
| ENSP00000269886 | SH3GL1 | 281 | 0.039 | 0.003876 | 800 |
| ENSP00000287139 | NODAL | 2 | 0.039 | 2.76E-05 | 650 |
| ENSP00000338127 | TESK1 | 279 | 0.039 | 0.003848 | 241 |
| ENSP00000247225 | SGPP1 | 281 | 0.04 | 0.003876 | 193 |
| ENSP00000253925 | PPFIA1 | 258 | 0.041 | 0.003559 | 872 |
| ENSP00000364802 | HSPA1A | 281 | 0.042 | 0.003876 | 770 |
| ENSP00000389934 | EXOC5 | 2 | 0.043 | 2.76E-05 | 321 |
| ENSP00000377492 | HMMR | 281 | 0.046 | 0.003876 | 630 |
| ENSP00000287598 | BUB1B | 58 | 0.047 | 0.0008 | 626 |
| ENSP00000320709 | ADIPOQ | 281 | 0.047 | 0.003876 | 907 |
| ENSP00000354360 | LAMC3 | 279 | 0.047 | 0.003848 | 903 |
| ENSP00000400717 | GNA13 | 278 | 0.048 | 0.003835 | 959 |
| ENSP00000230449 | EXOC2 | 2 | 0.05 | 2.76E-05 | 321 |
| ENSP00000345571 | E2F1 | 102 | 0.05 | 0.001407 | 916 |
| ENSP00000221403 | DHDH | 1 | 0.052 | 1.38E-05 | 213 |
| ENSP00000358309 | EPHA7 | 281 | 0.052 | 0.003876 | 850 |
| ENSP00000216181 | MYH9 | 280 | 0.053 | 0.003862 | 924 |
| ENSP00000256474 | VHL | 7117 | 0.053 | 0.098168 | 998 |
| ENSP00000246032 | STK35 | 258 | 0.054 | 0.003559 | 283 |
| ENSP00000263269 | GRIN2D | 4 | 0.054 | 5.52E-05 | 406 |
| ENSP00000372224 | HGFAC | 281 | 0.054 | 0.003876 | 540 |
| ENSP00000310572 | PSMC5 | 1 | 0.055 | 1.38E-05 | 668 |
| ENSP00000265512 | ADH4 | 2 | 0.056 | 2.76E-05 | 336 |
| ENSP00000284384 | PRKCA | 266 | 0.056 | 0.003669 | 988 |
| ENSP00000236147 | SELL | 281 | 0.057 | 0.003876 | 886 |
| ENSP00000263354 | NAPA | 258 | 0.057 | 0.003559 | 229 |
| ENSP00000323065 | GADD45GIP1 | 258 | 0.058 | 0.003559 | 213 |
| ENSP00000386259 | NEB | 277 | 0.058 | 0.003821 | 436 |
| ENSP00000355245 | PAX9 | 281 | 0.059 | 0.003876 | 563 |
| ENSP00000367462 | OLAH | 258 | 0.059 | 0.003559 | 0 |
| ENSP00000313950 | AURKB | 277 | 0.06 | 0.003821 | 943 |
| ENSP00000363071 | DES | 277 | 0.06 | 0.003821 | 672 |
| ENSP00000248272 | GAN | 2 | 0.061 | 2.76E-05 | 401 |
| ENSP00000325120 | PGR | 258 | 0.061 | 0.003559 | 923 |
| ENSP00000285949 | CYP26C1 | 279 | 0.062 | 0.003848 | 340 |
| ENSP00000363921 | PARD3 | 251 | 0.062 | 0.003462 | 924 |
| ENSP00000200453 | PPP1R15A | 242 | 0.064 | 0.003338 | 899 |
| ENSP00000257904 | CDK4 | 409 | 0.064 | 0.005642 | 915 |
| ENSP00000276571 | CRH | 9 | 0.064 | 0.000124 | 932 |
| ENSP00000387662 | GCG | 1396 | 0.067 | 0.019256 | 984 |
| ENSP00000263409 | LIFR | 258 | 0.069 | 0.003559 | 999 |
| ENSP00000296585 | ITGA2 | 1117 | 0.069 | 0.015407 | 987 |
| ENSP00000317337 | CD300LB | 274 | 0.07 | 0.003779 | 0 |
| ENSP00000346667 | WNK3 | 1 | 0.07 | 1.38E-05 | 671 |
| ENSP00000353847 | WWTR1 | 281 | 0.072 | 0.003876 | 932 |
| ENSP00000377141 | ARRB1 | 532 | 0.072 | 0.007338 | 996 |
| ENSP00000222005 | CDC37 | 57 | 0.073 | 0.000786 | 909 |
| ENSP00000247843 | YEATS4 | 281 | 0.075 | 0.003876 | 843 |
| ENSP00000293379 | ITGA5 | 3742 | 0.076 | 0.051615 | 964 |
| ENSP00000320758 | NOS1 | 515 | 0.076 | 0.007104 | 906 |
| ENSP00000349465 | PICK1 | 6 | 0.076 | 8.28E-05 | 702 |
| ENSP00000371973 | SAP18 | 7 | 0.076 | 9.66E-05 | 900 |
| ENSP00000342793 | PLD1 | 3 | 0.077 | 4.14E-05 | 877 |
| ENSP00000272190 | REN | 281 | 0.078 | 0.003876 | 902 |
| ENSP00000350369 | MAFG | 281 | 0.078 | 0.003876 | 671 |
| ENSP00000260605 | DYNC2LI1 | 280 | 0.079 | 0.003862 | 166 |
| ENSP00000236671 | CTSD | 258 | 0.081 | 0.003559 | 484 |
| ENSP00000416097 | GOLGA2 | 258 | 0.081 | 0.003559 | 269 |
| ENSP00000264426 | GRIA2 | 6 | 0.082 | 8.28E-05 | 917 |
| ENSP00000376684 | EPHB6 | 258 | 0.082 | 0.003559 | 997 |
| ENSP00000247668 | TRAF2 | 1045 | 0.083 | 0.014414 | 944 |
| ENSP00000374372 | SPTB | 253 | 0.083 | 0.00349 | 899 |
| ENSP00000229769 | FANCE | 280 | 0.089 | 0.003862 | 266 |
| ENSP00000334008 | PARVA | 279 | 0.089 | 0.003848 | 958 |
| ENSP00000373340 | BRPF1 | 281 | 0.089 | 0.003876 | 356 |
| ENSP00000005257 | RALA | 3 | 0.091 | 4.14E-05 | 805 |
| ENSP00000261464 | TRAF5 | 258 | 0.091 | 0.003559 | 421 |
| ENSP00000269485 | TNFRSF11A | 258 | 0.091 | 0.003559 | 813 |
| ENSP00000348708 | UPF2 | 7 | 0.091 | 9.66E-05 | 228 |
| ENSP00000402240 | KIAA1432 | 280 | 0.091 | 0.003862 | 567 |
| ENSP00000380942 | ARHGEF12 | 278 | 0.092 | 0.003835 | 857 |
| ENSP00000360683 | PTPN1 | 153 | 0.095 | 0.00211 | 995 |
| ENSP00000275874 | RAB19 | 258 | 0.096 | 0.003559 | 204 |
| ENSP00000356070 | MAPKAPK2 | 6 | 0.096 | 8.28E-05 | 993 |
| ENSP00000252444 | LDLR | 163 | 0.097 | 0.002248 | 998 |
| ENSP00000296145 | TDGF1 | 2 | 0.097 | 2.76E-05 | 946 |
| ENSP00000281928 | MED13L | 279 | 0.099 | 0.003848 | 906 |
| ENSP00000338799 | IL6ST | 919 | 0.099 | 0.012676 | 999 |
| ENSP00000362994 | TRAF1 | 258 | 0.099 | 0.003559 | 400 |
| ENSP00000246071 | SNRPB2 | 299 | 0.1 | 0.004124 | 388 |
| ENSP00000295897 | ALB | 2480 | 0.1 | 0.034208 | 980 |
| ENSP00000296181 | ITGB5 | 17 | 0.1 | 0.000234 | 996 |
| ENSP00000327048 | MAF | 281 | 0.1 | 0.003876 | 829 |
| ENSP00000303212 | SEMA3E | 281 | 0.101 | 0.003876 | 980 |
| ENSP00000259089 | BLK | 277 | 0.102 | 0.003821 | 909 |
| ENSP00000299106 | JAM3 | 258 | 0.104 | 0.003559 | 951 |
| ENSP00000360672 | PARD6B | 117 | 0.107 | 0.001614 | 846 |
| ENSP00000162749 | TNFRSF1A | 270 | 0.108 | 0.003724 | 972 |
| ENSP00000347169 | NUMB | 12 | 0.108 | 0.000166 | 999 |
| ENSP00000360154 | OCRL | 1 | 0.108 | 1.38E-05 | 902 |
| ENSP00000290200 | IL10RB | 258 | 0.109 | 0.003559 | 841 |
| ENSP00000326550 | TACC3 | 7 | 0.111 | 9.66E-05 | 912 |
| ENSP00000338477 | HNRNPF | 260 | 0.112 | 0.003586 | 204 |
| ENSP00000349437 | IGF2R | 74 | 0.112 | 0.001021 | 609 |
| ENSP00000362166 | MEAF6 | 281 | 0.112 | 0.003876 | 212 |
| ENSP00000308741 | CLOCK | 258 | 0.113 | 0.003559 | 899 |
| ENSP00000240922 | NAA50 | 281 | 0.114 | 0.003876 | 560 |
| ENSP00000251968 | TSG101 | 184 | 0.114 | 0.002538 | 899 |
| ENSP00000309968 | ADAM17 | 257 | 0.114 | 0.003545 | 958 |
| ENSP00000361066 | NCOA3 | 117 | 0.114 | 0.001614 | 950 |
| ENSP00000376609 | GRK5 | 1 | 0.114 | 1.38E-05 | 910 |
| ENSP00000300403 | TPX2 | 252 | 0.115 | 0.003476 | 229 |
| ENSP00000265023 | KNG1 | 291 | 0.116 | 0.004014 | 953 |
| ENSP00000363868 | ABCA1 | 281 | 0.116 | 0.003876 | 954 |
| ENSP00000379330 | NFATC2 | 308 | 0.117 | 0.004248 | 920 |
| ENSP00000261733 | ALDH2 | 2 | 0.118 | 2.76E-05 | 340 |
| ENSP00000375921 | PAX3 | 516 | 0.118 | 0.007117 | 795 |
| ENSP00000381066 | MAP2K7 | 258 | 0.118 | 0.003559 | 938 |
| ENSP00000204604 | CHRD | 281 | 0.119 | 0.003876 | 907 |
| ENSP00000244520 | SNRPC | 271 | 0.121 | 0.003738 | 404 |
| ENSP00000264638 | CNTNAP1 | 1 | 0.122 | 1.38E-05 | 621 |
| ENSP00000354560 | KIFAP3 | 3 | 0.123 | 4.14E-05 | 383 |
| ENSP00000309913 | TBX5 | 281 | 0.125 | 0.003876 | 923 |
| ENSP00000356213 | VIP | 281 | 0.13 | 0.003876 | 878 |
| ENSP00000204961 | EFNB1 | 256 | 0.131 | 0.003531 | 933 |
| ENSP00000264554 | SHC2 | 737 | 0.132 | 0.010166 | 967 |
| ENSP00000249299 | NAA38 | 58 | 0.133 | 0.0008 | 218 |
| ENSP00000255764 | MED10 | 279 | 0.133 | 0.003848 | 899 |
| ENSP00000299293 | FRS2 | 10 | 0.133 | 0.000138 | 999 |
| ENSP00000406878 | PSMB8 | 258 | 0.133 | 0.003559 | 160 |
| ENSP00000289779 | F11R | 2 | 0.134 | 2.76E-05 | 912 |
| ENSP00000350512 | COPS5 | 258 | 0.134 | 0.003559 | 987 |
| ENSP00000254719 | RPA1 | 205 | 0.135 | 0.002828 | 661 |
| ENSP00000417864 | ANP32A | 258 | 0.135 | 0.003559 | 165 |
| ENSP00000393725 | GFRA1 | 774 | 0.136 | 0.010676 | 900 |
| ENSP00000219255 | PARD6A | 249 | 0.137 | 0.003435 | 987 |
| ENSP00000261507 | MSMO1 | 281 | 0.137 | 0.003876 | 200 |
| ENSP00000291442 | NR2F6 | 258 | 0.138 | 0.003559 | 286 |
| ENSP00000327758 | NKX2-5 | 281 | 0.139 | 0.003876 | 915 |
| ENSP00000331831 | GAS6 | 32 | 0.14 | 0.000441 | 926 |
| ENSP00000344468 | SDC3 | 259 | 0.14 | 0.003573 | 929 |
| ENSP00000245919 | FOSB | 165 | 0.141 | 0.002276 | 999 |
| ENSP00000362082 | CCND3 | 515 | 0.141 | 0.007104 | 899 |
| ENSP00000360718 | RAB3B | 1 | 0.143 | 1.38E-05 | 629 |
| ENSP00000279146 | AIP | 281 | 0.144 | 0.003876 | 824 |
| ENSP00000355436 | OR2AK2 | 1 | 0.144 | 1.38E-05 | 0 |
| ENSP00000331358 | GAST | 280 | 0.145 | 0.003862 | 914 |
| ENSP00000312697 | DMAP1 | 562 | 0.146 | 0.007752 | 228 |
| ENSP00000360525 | MAGOH | 5 | 0.147 | 6.9E-05 | 167 |
| ENSP00000361626 | YBX1 | 12 | 0.147 | 0.000166 | 941 |
| ENSP00000227667 | APOC3 | 265 | 0.148 | 0.003655 | 996 |
| ENSP00000363827 | HSPG2 | 1 | 0.148 | 1.38E-05 | 928 |
| ENSP00000258962 | SRSF1 | 234 | 0.149 | 0.003228 | 430 |
| ENSP00000378529 | FZR1 | 7 | 0.149 | 9.66E-05 | 621 |
| ENSP00000290663 | MED8 | 279 | 0.15 | 0.003848 | 901 |
| ENSP00000220584 | FDFT1 | 281 | 0.151 | 0.003876 | 899 |
| ENSP00000384675 | SOS1 | 227 | 0.151 | 0.003131 | 995 |
| ENSP00000358903 | CYP17A1 | 1 | 0.152 | 1.38E-05 | 240 |
| ENSP00000283195 | RANBP2 | 237 | 0.154 | 0.003269 | 846 |
| ENSP00000320940 | NCOA1 | 1561 | 0.154 | 0.021532 | 979 |
| ENSP00000327583 | RANBP1 | 1 | 0.154 | 1.38E-05 | 626 |
| ENSP00000298130 | SPTSSA | 281 | 0.156 | 0.003876 | 267 |
| ENSP00000357927 | BNIPL | 56 | 0.157 | 0.000772 | 619 |
| ENSP00000328777 | EFNA5 | 281 | 0.158 | 0.003876 | 997 |
| ENSP00000335544 | CCKBR | 277 | 0.159 | 0.003821 | 902 |
| ENSP00000335657 | CCK | 281 | 0.16 | 0.003876 | 914 |
| ENSP00000342374 | SNRPD2 | 20 | 0.16 | 0.000276 | 296 |
| ENSP00000354130 | SOX10 | 516 | 0.16 | 0.007117 | 866 |
| ENSP00000268296 | ITGAX | 8 | 0.161 | 0.00011 | 800 |
| ENSP00000302961 | HSPA4 | 632 | 0.162 | 0.008717 | 993 |
| ENSP00000384442 | CDK11A | 281 | 0.162 | 0.003876 | 684 |
| ENSP00000254066 | RARA | 68 | 0.163 | 0.000938 | 956 |
| ENSP00000310596 | LSM1 | 3 | 0.163 | 4.14E-05 | 351 |
| ENSP00000380252 | NFE2L2 | 281 | 0.164 | 0.003876 | 922 |
| ENSP00000295797 | PRKCI | 328 | 0.166 | 0.004524 | 900 |
| ENSP00000413254 | RPH3A | 1 | 0.166 | 1.38E-05 | 229 |
| ENSP00000287647 | FANCD2 | 280 | 0.167 | 0.003862 | 623 |
| ENSP00000325527 | FBN1 | 12 | 0.167 | 0.000166 | 904 |
| ENSP00000368020 | KIF3A | 255 | 0.167 | 0.003517 | 681 |
| ENSP00000248572 | GNGT1 | 279 | 0.168 | 0.003848 | 340 |
| ENSP00000241014 | MAPK8IP1 | 258 | 0.169 | 0.003559 | 912 |
| ENSP00000254657 | PER2 | 257 | 0.169 | 0.003545 | 353 |
| ENSP00000344822 | S100A13 | 5 | 0.169 | 6.9E-05 | 899 |
| ENSP00000008527 | CRY1 | 257 | 0.17 | 0.003545 | 289 |
| ENSP00000338934 | EZR | 2562 | 0.174 | 0.035339 | 906 |
| ENSP00000407431 | HLA-C | 104 | 0.174 | 0.001435 | 273 |
| ENSP00000310491 | ARHGAP1 | 56 | 0.177 | 0.000772 | 960 |
| ENSP00000374357 | ARNTL | 258 | 0.179 | 0.003559 | 899 |
| ENSP00000367910 | FANCG | 1 | 0.18 | 1.38E-05 | 848 |
| ENSP00000226730 | IL2 | 28 | 0.182 | 0.000386 | 987 |
| ENSP00000336762 | ANG | 258 | 0.182 | 0.003559 | 669 |
| ENSP00000318868 | SHMT1 | 281 | 0.185 | 0.003876 | 955 |
| ENSP00000344115 | CDH5 | 386 | 0.185 | 0.005324 | 962 |
| ENSP00000303325 | TACR3 | 1 | 0.188 | 1.38E-05 | 899 |
| ENSP00000355778 | H3F3A | 280 | 0.188 | 0.003862 | 958 |
| ENSP00000224237 | VIM | 258 | 0.191 | 0.003559 | 995 |
| ENSP00000262613 | SLC9A3R1 | 2507 | 0.191 | 0.03458 | 985 |
| ENSP00000291552 | U2AF1 | 11 | 0.194 | 0.000152 | 552 |
| ENSP00000254480 | SMARCC1 | 282 | 0.195 | 0.00389 | 868 |
| ENSP00000239938 | EGR1 | 281 | 0.196 | 0.003876 | 942 |
| ENSP00000287641 | SST | 2 | 0.196 | 2.76E-05 | 788 |
| ENSP00000343204 | JAK1 | 325 | 0.196 | 0.004483 | 996 |
| ENSP00000350877 | SRSF2 | 10 | 0.198 | 0.000138 | 267 |
| ENSP00000358716 | DDX20 | 258 | 0.198 | 0.003559 | 293 |
| ENSP00000346294 | S100A4 | 280 | 0.199 | 0.003862 | 979 |
| ENSP00000376076 | SUMO1 | 104 | 0.2 | 0.001435 | 999 |
| ENSP00000245907 | C3 | 281 | 0.201 | 0.003876 | 903 |
| ENSP00000252997 | GATA5 | 255 | 0.201 | 0.003517 | 934 |
| ENSP00000259633 | CD72 | 257 | 0.201 | 0.003545 | 976 |
| ENSP00000267163 | RB1 | 1050 | 0.202 | 0.014483 | 999 |
| ENSP00000360247 | CYP2J2 | 280 | 0.202 | 0.003862 | 857 |
| ENSP00000229595 | ASF1A | 281 | 0.203 | 0.003876 | 489 |
| ENSP00000354621 | SMURF1 | 281 | 0.205 | 0.003876 | 999 |
| ENSP00000365891 | WAS | 153 | 0.207 | 0.00211 | 900 |
| ENSP00000299402 | APBB1 | 206 | 0.208 | 0.002841 | 962 |
| ENSP00000373614 | SELPLG | 281 | 0.208 | 0.003876 | 671 |
| ENSP00000361850 | PLAU | 304 | 0.209 | 0.004193 | 966 |
| ENSP00000314520 | KCNA2 | 257 | 0.213 | 0.003545 | 891 |
| ENSP00000338297 | IGF2 | 74 | 0.214 | 0.001021 | 970 |
| ENSP00000391592 | PTPN6 | 269 | 0.214 | 0.00371 | 997 |
| ENSP00000257430 | APC | 258 | 0.215 | 0.003559 | 927 |
| ENSP00000265773 | SMARCA2 | 3 | 0.216 | 4.14E-05 | 997 |
| ENSP00000262554 | SPTLC1 | 281 | 0.218 | 0.003876 | 491 |
| ENSP00000262320 | AXIN1 | 1726 | 0.219 | 0.023808 | 999 |
| ENSP00000291700 | S100B | 258 | 0.219 | 0.003559 | 860 |
| ENSP00000408910 | DCTN2 | 1 | 0.219 | 1.38E-05 | 911 |
| ENSP00000332643 | NDN | 102 | 0.22 | 0.001407 | 923 |
| ENSP00000375986 | MAP3K4 | 258 | 0.221 | 0.003559 | 655 |
| ENSP00000352929 | CSNK1E | 257 | 0.223 | 0.003545 | 900 |
| ENSP00000256857 | GRP | 21 | 0.225 | 0.00029 | 919 |
| ENSP00000318297 | RUVBL1 | 562 | 0.227 | 0.007752 | 980 |
| ENSP00000329411 | IRF7 | 14 | 0.227 | 0.000193 | 345 |
| ENSP00000231487 | SKP1 | 736 | 0.228 | 0.010152 | 998 |
| ENSP00000308533 | GEMIN2 | 258 | 0.23 | 0.003559 | 310 |
| ENSP00000296440 | PLXNB1 | 1 | 0.233 | 1.38E-05 | 941 |
| ENSP00000330393 | LEPR | 287 | 0.235 | 0.003959 | 998 |
| ENSP00000231454 | IL5 | 1 | 0.236 | 1.38E-05 | 913 |
| ENSP00000357753 | IVL | 258 | 0.236 | 0.003559 | 908 |
| ENSP00000219476 | TSC2 | 127 | 0.237 | 0.001752 | 999 |
| ENSP00000262643 | CCNE1 | 213 | 0.237 | 0.002938 | 900 |
| ENSP00000276420 | DOK2 | 396 | 0.237 | 0.005462 | 906 |
| ENSP00000371432 | PRLR | 735 | 0.239 | 0.010138 | 950 |
| ENSP00000374990 | IGHG1 | 2 | 0.243 | 2.76E-05 | 904 |
| ENSP00000300134 | STAT6 | 253 | 0.245 | 0.00349 | 968 |
| ENSP00000302955 | RRM2 | 28 | 0.245 | 0.000386 | 824 |
| ENSP00000242480 | EGR2 | 516 | 0.246 | 0.007117 | 923 |
| ENSP00000356918 | STX7 | 258 | 0.246 | 0.003559 | 800 |
| ENSP00000370343 | IRF4 | 16 | 0.246 | 0.000221 | 917 |
| ENSP00000391901 | PHF1 | 191 | 0.246 | 0.002635 | 462 |
| ENSP00000289902 | FCER1G | 514 | 0.247 | 0.00709 | 979 |
| ENSP00000264110 | ATF2 | 100 | 0.248 | 0.001379 | 971 |
| ENSP00000227752 | IL10RA | 258 | 0.249 | 0.003559 | 892 |
| ENSP00000412237 | IL10 | 258 | 0.249 | 0.003559 | 969 |
| ENSP00000286827 | TIAM1 | 2 | 0.252 | 2.76E-05 | 992 |
| ENSP00000351908 | MAP3K5 | 6 | 0.252 | 8.28E-05 | 971 |
| ENSP00000348965 | DYNC1H1 | 281 | 0.254 | 0.003876 | 974 |
| ENSP00000316054 | DVL3 | 281 | 0.257 | 0.003876 | 989 |
| ENSP00000373091 | HLA-E | 1 | 0.257 | 1.38E-05 | 816 |
| ENSP00000315644 | TYMS | 537 | 0.26 | 0.007407 | 505 |
| ENSP00000267868 | RAD51 | 8 | 0.261 | 0.00011 | 548 |
| ENSP00000400365 | LAMA2 | 552 | 0.261 | 0.007614 | 925 |
| ENSP00000419692 | RXRA | 586 | 0.264 | 0.008083 | 999 |
| ENSP00000287727 | ZFYVE9 | 245 | 0.265 | 0.003379 | 993 |
| ENSP00000287934 | FZD1 | 281 | 0.268 | 0.003876 | 979 |
| ENSP00000263864 | VAMP8 | 258 | 0.27 | 0.003559 | 340 |
| ENSP00000300413 | SNRPD1 | 258 | 0.27 | 0.003559 | 408 |
| ENSP00000266000 | DAXX | 280 | 0.274 | 0.003862 | 984 |
| ENSP00000364336 | TBXA2R | 279 | 0.276 | 0.003848 | 909 |
| ENSP00000337915 | CYP3A4 | 1 | 0.28 | 1.38E-05 | 893 |
| ENSP00000162330 | BCAR1 | 801 | 0.283 | 0.011049 | 999 |
| ENSP00000343619 | HOXA9 | 281 | 0.285 | 0.003876 | 924 |
| ENSP00000342215 | KIR2DL3 | 104 | 0.286 | 0.001435 | 317 |
| ENSP00000317272 | MET | 793 | 0.287 | 0.010938 | 984 |
| ENSP00000308450 | CDC20 | 600 | 0.289 | 0.008276 | 283 |
| ENSP00000261908 | NEO1 | 554 | 0.293 | 0.007642 | 868 |
| ENSP00000263208 | HIRA | 281 | 0.293 | 0.003876 | 369 |
| ENSP00000362649 | HDAC1 | 1236 | 0.295 | 0.017049 | 997 |
| ENSP00000356024 | CR2 | 272 | 0.298 | 0.003752 | 373 |
| ENSP00000248244 | TICAM1 | 253 | 0.301 | 0.00349 | 918 |
| ENSP00000366466 | CTNNBIP1 | 268 | 0.302 | 0.003697 | 899 |
| ENSP00000357292 | UBQLN4 | 2 | 0.303 | 2.76E-05 | 812 |
| ENSP00000219548 | STUB1 | 983 | 0.304 | 0.013559 | 868 |
| ENSP00000232014 | BCL6 | 16 | 0.305 | 0.000221 | 952 |
| ENSP00000348551 | NCOR2 | 48 | 0.305 | 0.000662 | 995 |
| ENSP00000318585 | BACE1 | 258 | 0.306 | 0.003559 | 891 |
| ENSP00000264335 | YWHAE | 281 | 0.307 | 0.003876 | 987 |
| ENSP00000340820 | MAPT | 776 | 0.31 | 0.010704 | 981 |
| ENSP00000361162 | TOE1 | 10 | 0.31 | 0.000138 | 921 |
| ENSP00000352712 | DACH1 | 562 | 0.313 | 0.007752 | 969 |
| ENSP00000363092 | PRKG1 | 1 | 0.313 | 1.38E-05 | 906 |
| ENSP00000265171 | EGF | 571 | 0.314 | 0.007876 | 987 |
| ENSP00000262965 | TCF3 | 562 | 0.317 | 0.007752 | 985 |
| ENSP00000351905 | TGFBR2 | 281 | 0.317 | 0.003876 | 999 |
| ENSP00000361818 | SDC4 | 515 | 0.317 | 0.007104 | 984 |
| ENSP00000296543 | NAA15 | 33 | 0.318 | 0.000455 | 285 |
| ENSP00000231449 | IL4 | 281 | 0.32 | 0.003876 | 919 |
| ENSP00000380378 | PAFAH1B1 | 5 | 0.32 | 6.9E-05 | 899 |
| ENSP00000411698 | USO1 | 258 | 0.322 | 0.003559 | 750 |
| ENSP00000225831 | CCL2 | 296 | 0.323 | 0.004083 | 989 |
| ENSP00000278385 | CD44 | 731 | 0.323 | 0.010083 | 936 |
| ENSP00000292807 | AP2M1 | 4 | 0.323 | 5.52E-05 | 928 |
| ENSP00000347198 | SRGAP1 | 1006 | 0.325 | 0.013876 | 193 |
| ENSP00000348108 | KHDRBS3 | 271 | 0.328 | 0.003738 | 752 |
| ENSP00000367207 | MYC | 338 | 0.328 | 0.004662 | 991 |
| ENSP00000282091 | PTH | 4 | 0.329 | 5.52E-05 | 962 |
| ENSP00000391069 | SRPK1 | 223 | 0.331 | 0.003076 | 379 |
| ENSP00000281708 | FBXW7 | 171 | 0.332 | 0.002359 | 999 |
| ENSP00000304669 | CTNNA1 | 258 | 0.333 | 0.003559 | 991 |
| ENSP00000276201 | UPF3B | 7 | 0.334 | 9.66E-05 | 299 |
| ENSP00000302150 | PRL | 747 | 0.335 | 0.010304 | 965 |
| ENSP00000268058 | PML | 280 | 0.336 | 0.003862 | 997 |
| ENSP00000295926 | CCNL1 | 277 | 0.336 | 0.003821 | 189 |
| ENSP00000296785 | ANKRA2 | 13 | 0.336 | 0.000179 | 990 |
| ENSP00000245544 | NUP85 | 258 | 0.337 | 0.003559 | 579 |
| ENSP00000361423 | ABL1 | 343 | 0.34 | 0.004731 | 978 |
| ENSP00000303452 | TRH | 4 | 0.346 | 5.52E-05 | 914 |
| ENSP00000216223 | IL2RB | 47 | 0.347 | 0.000648 | 962 |
| ENSP00000269260 | ARRB2 | 257 | 0.348 | 0.003545 | 999 |
| ENSP00000011653 | CD4 | 309 | 0.349 | 0.004262 | 985 |
| ENSP00000362441 | ATRX | 280 | 0.349 | 0.003862 | 370 |
| ENSP00000223642 | C5 | 1 | 0.35 | 1.38E-05 | 524 |
| ENSP00000365851 | BMI1 | 276 | 0.356 | 0.003807 | 603 |
| ENSP00000267814 | SORD | 229 | 0.358 | 0.003159 | 341 |
| ENSP00000342952 | ADCY2 | 3 | 0.358 | 4.14E-05 | 939 |
| ENSP00000350283 | BRCA1 | 284 | 0.358 | 0.003917 | 964 |
| ENSP00000243776 | CHPF | 9 | 0.359 | 0.000124 | 910 |
| ENSP00000312995 | CLSPN | 2 | 0.359 | 2.76E-05 | 219 |
| ENSP00000327850 | NFATC1 | 516 | 0.36 | 0.007117 | 938 |
| ENSP00000343040 | HMGB1 | 516 | 0.362 | 0.007117 | 801 |
| ENSP00000292303 | CCR5 | 303 | 0.364 | 0.004179 | 945 |
| ENSP00000352516 | DNMT1 | 17 | 0.365 | 0.000234 | 938 |
| ENSP00000282588 | ITGA1 | 286 | 0.366 | 0.003945 | 963 |
| ENSP00000261769 | CDH1 | 538 | 0.367 | 0.007421 | 996 |
| ENSP00000265709 | ANK1 | 255 | 0.37 | 0.003517 | 563 |
| ENSP00000260363 | KIF23 | 3 | 0.371 | 4.14E-05 | 308 |
| ENSP00000271628 | SF3B4 | 254 | 0.371 | 0.003504 | 361 |
| ENSP00000283635 | CD8A | 1 | 0.371 | 1.38E-05 | 911 |
| ENSP00000369497 | BRCA2 | 10 | 0.372 | 0.000138 | 461 |
| ENSP00000352798 | COL18A1 | 258 | 0.377 | 0.003559 | 970 |
| ENSP00000306245 | FOS | 4271 | 0.378 | 0.058912 | 999 |
| ENSP00000391349 | DOM3Z | 7 | 0.379 | 9.66E-05 | 786 |
| ENSP00000412788 | PTBP2 | 1 | 0.38 | 1.38E-05 | 161 |
| ENSP00000303830 | INSR | 466 | 0.384 | 0.006428 | 997 |
| ENSP00000264156 | MCM6 | 4 | 0.385 | 5.52E-05 | 324 |
| ENSP00000305372 | ADRB2 | 1 | 0.386 | 1.38E-05 | 906 |
| ENSP00000346389 | MEF2A | 5 | 0.386 | 6.9E-05 | 908 |
| ENSP00000355537 | ACTN2 | 258 | 0.386 | 0.003559 | 899 |
| ENSP00000340858 | B2M | 106 | 0.389 | 0.001462 | 899 |
| ENSP00000256442 | CCNB1 | 4308 | 0.393 | 0.059422 | 895 |
| ENSP00000348827 | THRB | 2 | 0.398 | 2.76E-05 | 908 |
| ENSP00000261479 | PSMA6 | 257 | 0.399 | 0.003545 | 619 |
| ENSP00000352064 | KLRC1 | 1 | 0.402 | 1.38E-05 | 337 |
| ENSP00000311113 | JUP | 20 | 0.404 | 0.000276 | 999 |
| ENSP00000332973 | SMAD3 | 48 | 0.406 | 0.000662 | 946 |
| ENSP00000417763 | NAA10 | 33 | 0.407 | 0.000455 | 685 |
| ENSP00000206249 | ESR1 | 7519 | 0.408 | 0.103713 | 997 |
| ENSP00000313752 | SSNA1 | 258 | 0.409 | 0.003559 | 899 |
| ENSP00000312435 | DAG1 | 552 | 0.412 | 0.007614 | 934 |
| ENSP00000265333 | VDAC1 | 229 | 0.421 | 0.003159 | 961 |
| ENSP00000265056 | MCM2 | 277 | 0.422 | 0.003821 | 229 |
| ENSP00000315859 | RNPS1 | 19 | 0.422 | 0.000262 | 233 |
| ENSP00000373952 | FANCA | 281 | 0.424 | 0.003876 | 956 |
| ENSP00000365380 | FOXP3 | 1032 | 0.425 | 0.014235 | 963 |
| ENSP00000332353 | PTCH1 | 2122 | 0.427 | 0.02927 | 998 |
| ENSP00000300093 | PLK1 | 1115 | 0.428 | 0.01538 | 924 |
| ENSP00000313419 | CD19 | 272 | 0.428 | 0.003752 | 985 |
| ENSP00000343274 | INTS8 | 270 | 0.43 | 0.003724 | 204 |
| ENSP00000220592 | AGO2 | 258 | 0.434 | 0.003559 | 302 |
| ENSP00000284981 | APP | 258 | 0.434 | 0.003559 | 987 |
| ENSP00000351273 | CASP8 | 258 | 0.439 | 0.003559 | 996 |
| ENSP00000345008 | FBLN5 | 281 | 0.44 | 0.003876 | 851 |
| ENSP00000334122 | FGF3 | 281 | 0.443 | 0.003876 | 999 |
| ENSP00000170630 | IL4R | 253 | 0.444 | 0.00349 | 904 |
| ENSP00000320147 | EZH2 | 191 | 0.446 | 0.002635 | 943 |
| ENSP00000279593 | GRIN2B | 256 | 0.448 | 0.003531 | 805 |
| ENSP00000223129 | RPA3 | 205 | 0.45 | 0.002828 | 0 |
| ENSP00000247161 | ELK1 | 48 | 0.452 | 0.000662 | 997 |
| ENSP00000259808 | RIPK1 | 771 | 0.454 | 0.010635 | 968 |
| ENSP00000334458 | GATA4 | 562 | 0.454 | 0.007752 | 942 |
| ENSP00000337014 | HFE2 | 24 | 0.455 | 0.000331 | 905 |
| ENSP00000231572 | RARS | 1 | 0.456 | 1.38E-05 | 929 |
| ENSP00000354791 | DCTN1 | 280 | 0.456 | 0.003862 | 766 |
| ENSP00000357656 | FYN | 1572 | 0.457 | 0.021683 | 994 |
| ENSP00000382004 | CTNND1 | 184 | 0.457 | 0.002538 | 999 |
| ENSP00000351407 | ARNT | 1321 | 0.458 | 0.018221 | 984 |
| ENSP00000264606 | HDAC4 | 108 | 0.459 | 0.00149 | 974 |
| ENSP00000348786 | RAP1A | 817 | 0.463 | 0.011269 | 979 |
| ENSP00000353154 | NFASC | 6 | 0.465 | 8.28E-05 | 412 |
| ENSP00000360025 | GADD45A | 516 | 0.465 | 0.007117 | 878 |
| ENSP00000262629 | TYROBP | 371 | 0.467 | 0.005117 | 936 |
| ENSP00000307387 | PDCD6IP | 281 | 0.467 | 0.003876 | 681 |
| ENSP00000340330 | KAT5 | 766 | 0.467 | 0.010566 | 998 |
| ENSP00000312262 | ADRBK1 | 2 | 0.472 | 2.76E-05 | 983 |
| ENSP00000269397 | CBX4 | 276 | 0.474 | 0.003807 | 391 |
| ENSP00000314949 | POLR2A | 30 | 0.475 | 0.000414 | 914 |
| ENSP00000263033 | SYTL4 | 255 | 0.476 | 0.003517 | 202 |
| ENSP00000299543 | CTDP1 | 430 | 0.476 | 0.005931 | 738 |
| ENSP00000354586 | GLI2 | 22 | 0.478 | 0.000303 | 997 |
| ENSP00000262477 | RABEP1 | 190 | 0.48 | 0.002621 | 569 |
| ENSP00000052754 | DCN | 1010 | 0.495 | 0.013931 | 967 |
| ENSP00000278616 | ATM | 258 | 0.496 | 0.003559 | 997 |
| ENSP00000284957 | RABGEF1 | 187 | 0.496 | 0.002579 | 621 |
| ENSP00000338130 | KLRD1 | 1 | 0.497 | 1.38E-05 | 318 |
| ENSP00000389140 | DCC | 1273 | 0.497 | 0.017559 | 985 |
| ENSP00000309103 | BAD | 45 | 0.498 | 0.000621 | 998 |
| ENSP00000228837 | FGF6 | 558 | 0.499 | 0.007697 | 999 |
| ENSP00000337761 | RAB27A | 255 | 0.499 | 0.003517 | 524 |
| ENSP00000351665 | CLIP1 | 279 | 0.499 | 0.003848 | 809 |
| ENSP00000294172 | NXF1 | 793 | 0.501 | 0.010938 | 672 |
| ENSP00000363435 | ITPR3 | 3 | 0.502 | 4.14E-05 | 919 |
| ENSP00000349467 | CALM1 | 517 | 0.505 | 0.007131 | 992 |
| ENSP00000357392 | EFNA1 | 255 | 0.506 | 0.003517 | 999 |
| ENSP00000304592 | FASN | 258 | 0.507 | 0.003559 | 905 |
| ENSP00000317714 | STX4 | 260 | 0.508 | 0.003586 | 358 |
| ENSP00000352980 | HIST1H4A | 63 | 0.508 | 0.000869 | 718 |
| ENSP00000366396 | XRN2 | 3 | 0.508 | 4.14E-05 | 191 |
| ENSP00000251074 | NUP37 | 1 | 0.513 | 1.38E-05 | 359 |
| ENSP00000380921 | SH3KBP1 | 97 | 0.514 | 0.001338 | 965 |
| ENSP00000356713 | IFNGR1 | 258 | 0.519 | 0.003559 | 932 |
| ENSP00000231509 | NR3C1 | 72 | 0.521 | 0.000993 | 994 |
| ENSP00000300651 | MED1 | 348 | 0.524 | 0.0048 | 996 |
| ENSP00000312122 | SEC13 | 281 | 0.525 | 0.003876 | 899 |
| ENSP00000263309 | CLNS1A | 88 | 0.528 | 0.001214 | 157 |
| ENSP00000242152 | NPY | 260 | 0.529 | 0.003586 | 902 |
| ENSP00000276414 | GNRH1 | 256 | 0.529 | 0.003531 | 946 |
| ENSP00000209728 | CDC6 | 10 | 0.53 | 0.000138 | 404 |
| ENSP00000350275 | HIST1H3A | 284 | 0.53 | 0.003917 | 900 |
| ENSP00000215829 | SNRPD3 | 71 | 0.531 | 0.000979 | 326 |
| ENSP00000368438 | PCNA | 1503 | 0.531 | 0.020732 | 951 |
| ENSP00000046794 | LCP2 | 417 | 0.534 | 0.005752 | 995 |
| ENSP00000264926 | RAD18 | 258 | 0.534 | 0.003559 | 317 |
| ENSP00000274026 | CCNA2 | 2 | 0.535 | 2.76E-05 | 922 |
| ENSP00000316032 | NUP98 | 280 | 0.535 | 0.003862 | 714 |
| ENSP00000297518 | CDK5 | 778 | 0.536 | 0.010731 | 824 |
| ENSP00000319169 | PRMT5 | 88 | 0.536 | 0.001214 | 977 |
| ENSP00000216911 | AURKA | 514 | 0.539 | 0.00709 | 915 |
| ENSP00000381331 | HDAC2 | 420 | 0.54 | 0.005793 | 998 |
| ENSP00000278916 | CHEK1 | 6 | 0.543 | 8.28E-05 | 880 |
| ENSP00000304895 | IRS1 | 715 | 0.544 | 0.009862 | 996 |
| ENSP00000401303 | SHC1 | 3847 | 0.549 | 0.053064 | 994 |
| ENSP00000378165 | ZNF207 | 79 | 0.552 | 0.00109 | 410 |
| ENSP00000334051 | GNAL | 1 | 0.553 | 1.38E-05 | 344 |
| ENSP00000338868 | PHF8 | 3 | 0.553 | 4.14E-05 | 550 |
| ENSP00000385269 | ELAVL1 | 5 | 0.553 | 6.9E-05 | 899 |
| ENSP00000301633 | BIRC5 | 14 | 0.554 | 0.000193 | 946 |
| ENSP00000351486 | NTRK1 | 3902 | 0.563 | 0.053822 | 998 |
| ENSP00000321656 | CDC25C | 6 | 0.566 | 8.28E-05 | 958 |
| ENSP00000324648 | CYP2B6 | 1 | 0.57 | 1.38E-05 | 896 |
| ENSP00000352400 | NUP214 | 776 | 0.575 | 0.010704 | 641 |
| ENSP00000413035 | RBFOX2 | 4 | 0.575 | 5.52E-05 | 413 |
| ENSP00000296871 | CSF2 | 184 | 0.578 | 0.002538 | 953 |
| ENSP00000405934 | ITPR1 | 260 | 0.578 | 0.003586 | 952 |
| ENSP00000245960 | CDC25B | 41 | 0.58 | 0.000566 | 870 |
| ENSP00000307046 | SDC2 | 977 | 0.582 | 0.013476 | 962 |
| ENSP00000226218 | SEBOX | 191 | 0.583 | 0.002635 | 996 |
| ENSP00000222254 | PIK3R2 | 27 | 0.586 | 0.000372 | 970 |
| ENSP00000302564 | BCL2L1 | 243 | 0.586 | 0.003352 | 999 |
| ENSP00000384053 | CSF2RB | 184 | 0.587 | 0.002538 | 959 |
| ENSP00000339109 | ANAPC1 | 19 | 0.596 | 0.000262 | 160 |
| ENSP00000363763 | EPHB2 | 147 | 0.596 | 0.002028 | 971 |
| ENSP00000239223 | DUSP1 | 14 | 0.602 | 0.000193 | 999 |
| ENSP00000260130 | SDCBP | 526 | 0.605 | 0.007255 | 622 |
| ENSP00000282441 | YAP1 | 281 | 0.608 | 0.003876 | 974 |
| ENSP00000357858 | BUB3 | 79 | 0.608 | 0.00109 | 302 |
| ENSP00000341551 | SMAD4 | 1003 | 0.613 | 0.013835 | 998 |
| ENSP00000211122 | GSTA3 | 1 | 0.617 | 1.38E-05 | 906 |
| ENSP00000369050 | CYP1A1 | 559 | 0.619 | 0.007711 | 907 |
| ENSP00000216225 | RBX1 | 640 | 0.62 | 0.008828 | 985 |
| ENSP00000364893 | ARHGEF7 | 180 | 0.622 | 0.002483 | 995 |
| ENSP00000374455 | SQSTM1 | 327 | 0.625 | 0.00451 | 913 |
| ENSP00000003084 | CFTR | 4027 | 0.629 | 0.055546 | 847 |
| ENSP00000333001 | RBM8A | 4 | 0.629 | 5.52E-05 | 641 |
| ENSP00000354476 | SREBF2 | 561 | 0.629 | 0.007738 | 947 |
| ENSP00000229854 | MCM3 | 68 | 0.63 | 0.000938 | 619 |
| ENSP00000228682 | GLI1 | 529 | 0.631 | 0.007297 | 978 |
| ENSP00000348554 | CDC16 | 7 | 0.632 | 9.66E-05 | 226 |
| ENSP00000335153 | HSP90AA1 | 3376 | 0.635 | 0.046567 | 999 |
| ENSP00000353224 | TFRC | 106 | 0.64 | 0.001462 | 927 |
| ENSP00000363822 | AR | 6 | 0.641 | 8.28E-05 | 993 |
| ENSP00000318861 | SF3B2 | 3 | 0.642 | 4.14E-05 | 266 |
| ENSP00000398597 | EXOSC6 | 3 | 0.643 | 4.14E-05 | 0 |
| ENSP00000339151 | IKBKB | 13 | 0.646 | 0.000179 | 986 |
| ENSP00000354394 | STAT1 | 608 | 0.647 | 0.008386 | 994 |
| ENSP00000398698 | TNF | 270 | 0.648 | 0.003724 | 963 |
| ENSP00000417404 | HFE | 106 | 0.653 | 0.001462 | 369 |
| ENSP00000223095 | SERPINE1 | 617 | 0.655 | 0.008511 | 998 |
| ENSP00000263686 | SELP | 818 | 0.655 | 0.011283 | 906 |
| ENSP00000353483 | MAPK8 | 730 | 0.659 | 0.010069 | 998 |
| ENSP00000262633 | RBM42 | 4 | 0.663 | 5.52E-05 | 200 |
| ENSP00000292644 | PSMC2 | 510 | 0.666 | 0.007035 | 415 |
| ENSP00000314491 | SRRT | 329 | 0.668 | 0.004538 | 196 |
| ENSP00000360286 | RAE1 | 537 | 0.668 | 0.007407 | 641 |
| ENSP00000249647 | SNAP23 | 256 | 0.669 | 0.003531 | 305 |
| ENSP00000366013 | GNB2L1 | 57 | 0.672 | 0.000786 | 997 |
| ENSP00000359988 | SRSF11 | 10 | 0.673 | 0.000138 | 235 |
| ENSP00000309503 | YWHAZ | 45 | 0.68 | 0.000621 | 983 |
| ENSP00000341344 | GGA1 | 261 | 0.682 | 0.0036 | 619 |
| ENSP00000223023 | WASL | 723 | 0.683 | 0.009973 | 934 |
| ENSP00000328511 | KCNA4 | 257 | 0.685 | 0.003545 | 336 |
| ENSP00000363998 | ITCH | 774 | 0.685 | 0.010676 | 985 |
| ENSP00000290921 | CTBP1 | 276 | 0.689 | 0.003807 | 942 |
| ENSP00000261205 | SYT1 | 537 | 0.691 | 0.007407 | 369 |
| ENSP00000329967 | TBK1 | 251 | 0.698 | 0.003462 | 750 |
| ENSP00000302530 | BUB1 | 154 | 0.702 | 0.002124 | 619 |
| ENSP00000302269 | VAV1 | 346 | 0.704 | 0.004773 | 998 |
| ENSP00000218388 | TIMP1 | 8 | 0.709 | 0.00011 | 962 |
| ENSP00000252945 | CYP2E1 | 26 | 0.709 | 0.000359 | 490 |
| ENSP00000323050 | RBBP8 | 137 | 0.709 | 0.00189 | 908 |
| ENSP00000171111 | KEAP1 | 1 | 0.714 | 1.38E-05 | 467 |
| ENSP00000324804 | PPP2R1A | 303 | 0.717 | 0.004179 | 959 |
| ENSP00000396127 | RAN | 1021 | 0.719 | 0.014083 | 899 |
| ENSP00000262435 | SMURF2 | 19 | 0.722 | 0.000262 | 999 |
| ENSP00000314458 | CDC42 | 1432 | 0.723 | 0.019752 | 970 |
| ENSP00000303939 | CTLA4 | 696 | 0.724 | 0.0096 | 929 |
| ENSP00000308176 | BTK | 1 | 0.726 | 1.38E-05 | 946 |
| ENSP00000337825 | LCK | 533 | 0.726 | 0.007352 | 996 |
| ENSP00000360798 | EPS15 | 277 | 0.726 | 0.003821 | 971 |
| ENSP00000370938 | CDK8 | 131 | 0.726 | 0.001807 | 967 |
| ENSP00000235090 | WDR77 | 87 | 0.728 | 0.0012 | 196 |
| ENSP00000350003 | CCR3 | 217 | 0.728 | 0.002993 | 461 |
| ENSP00000229022 | VDR | 532 | 0.73 | 0.007338 | 933 |
| ENSP00000287497 | ITGAM | 1072 | 0.733 | 0.014787 | 921 |
| ENSP00000326804 | CUL1 | 32 | 0.734 | 0.000441 | 982 |
| ENSP00000401980 | MAVS | 281 | 0.735 | 0.003876 | 750 |
| ENSP00000222256 | RAB3A | 535 | 0.74 | 0.00738 | 922 |
| ENSP00000348577 | RANGAP1 | 1040 | 0.744 | 0.014345 | 912 |
| ENSP00000284811 | TCEB1 | 57 | 0.749 | 0.000786 | 978 |
| ENSP00000268182 | IQGAP1 | 71 | 0.751 | 0.000979 | 981 |
| ENSP00000256452 | IL5RA | 520 | 0.754 | 0.007173 | 822 |
| ENSP00000085219 | CD22 | 5 | 0.755 | 6.9E-05 | 732 |
| ENSP00000338983 | MUC1 | 258 | 0.756 | 0.003559 | 485 |
| ENSP00000229179 | NUP107 | 1 | 0.762 | 1.38E-05 | 387 |
| ENSP00000311032 | CASP3 | 8 | 0.767 | 0.00011 | 994 |
| ENSP00000332468 | TRAF3 | 530 | 0.767 | 0.007311 | 684 |
| ENSP00000223029 | AIMP2 | 280 | 0.769 | 0.003862 | 621 |
| ENSP00000248566 | SHFM1 | 524 | 0.77 | 0.007228 | 462 |
| ENSP00000303242 | ITGB2 | 1314 | 0.771 | 0.018125 | 986 |
| ENSP00000322542 | GTF2I | 7 | 0.772 | 9.66E-05 | 946 |
| ENSP00000367316 | ITGA8 | 1309 | 0.778 | 0.018056 | 843 |
| ENSP00000300161 | YWHAB | 287 | 0.779 | 0.003959 | 985 |
| ENSP00000350941 | SRC | 5959 | 0.781 | 0.082195 | 999 |
| ENSP00000414634 | LSM2 | 11 | 0.782 | 0.000152 | 0 |
| ENSP00000326031 | PPP1CA | 13 | 0.785 | 0.000179 | 958 |
| ENSP00000367408 | CASK | 191 | 0.793 | 0.002635 | 609 |
| ENSP00000348069 | SREBF1 | 10 | 0.796 | 0.000138 | 935 |
| ENSP00000264246 | CD80 | 528 | 0.798 | 0.007283 | 516 |
| ENSP00000366135 | EXOSC10 | 57 | 0.801 | 0.000786 | 172 |
| ENSP00000293272 | CCL5 | 217 | 0.808 | 0.002993 | 962 |
| ENSP00000337088 | MEN1 | 281 | 0.808 | 0.003876 | 675 |
| ENSP00000252622 | LSM7 | 10 | 0.809 | 0.000138 | 222 |
| ENSP00000315955 | FOXA2 | 270 | 0.811 | 0.003724 | 984 |
| ENSP00000362900 | SRSF4 | 1 | 0.812 | 1.38E-05 | 0 |
| ENSP00000329357 | SP1 | 658 | 0.815 | 0.009076 | 995 |
| ENSP00000240185 | TARDBP | 48 | 0.816 | 0.000662 | 319 |
| ENSP00000252818 | JUND | 281 | 0.817 | 0.003876 | 982 |
| ENSP00000370473 | IGFBP3 | 1464 | 0.818 | 0.020194 | 999 |
| ENSP00000221494 | SF3A2 | 966 | 0.83 | 0.013325 | 944 |
| ENSP00000233946 | IL1R1 | 257 | 0.845 | 0.003545 | 896 |
| ENSP00000324890 | CD28 | 528 | 0.847 | 0.007283 | 621 |
| ENSP00000418447 | PPP2CA | 558 | 0.851 | 0.007697 | 994 |
| ENSP00000301019 | CDT1 | 4 | 0.854 | 5.52E-05 | 621 |
| ENSP00000311677 | PPP1R8 | 12 | 0.855 | 0.000166 | 0 |
| ENSP00000222812 | STX1A | 1552 | 0.856 | 0.021407 | 287 |
| ENSP00000307863 | U2AF2 | 1141 | 0.861 | 0.015738 | 720 |
| ENSP00000261461 | PPP2R5A | 101 | 0.865 | 0.001393 | 899 |
| ENSP00000227758 | BIRC2 | 257 | 0.866 | 0.003545 | 774 |
| ENSP00000358997 | IRAK1 | 1527 | 0.87 | 0.021063 | 985 |
| ENSP00000348986 | INS-IGF2 | 502 | 0.871 | 0.006924 | 996 |
| ENSP00000313829 | KHDRBS1 | 1602 | 0.873 | 0.022097 | 997 |
| ENSP00000263753 | SGOL1 | 462 | 0.874 | 0.006373 | 0 |
| ENSP00000303634 | LRP8 | 39 | 0.881 | 0.000538 | 993 |
| ENSP00000309845 | HRAS | 1148 | 0.885 | 0.015835 | 998 |
| ENSP00000371532 | VLDLR | 338 | 0.885 | 0.004662 | 988 |
| ENSP00000268712 | NCOR1 | 31 | 0.886 | 0.000428 | 943 |
| ENSP00000344818 | UBC | 36838 | 0.886 | 0.508124 | 999 |
| ENSP00000227378 | HSPA8 | 329 | 0.894 | 0.004538 | 845 |
| ENSP00000339007 | GRB2 | 4255 | 0.898 | 0.058691 | 999 |
| ENSP00000263967 | PIK3CA | 247 | 0.899 | 0.003407 | 998 |
| ENSP00000251849 | RAF1 | 2133 | 0.902 | 0.029422 | 999 |
| ENSP00000371138 | FKBP1A | 12 | 0.915 | 0.000166 | 998 |
| ENSP00000324897 | UBE2I | 1184 | 0.917 | 0.016331 | 965 |
| ENSP00000358541 | SIKE1 | 80 | 0.923 | 0.001103 | 194 |
| ENSP00000269321 | ARHGDIA | 322 | 0.925 | 0.004442 | 929 |
| ENSP00000300574 | CRK | 1181 | 0.928 | 0.01629 | 999 |
| ENSP00000384273 | RELA | 231 | 0.928 | 0.003186 | 982 |
| ENSP00000356087 | IKBKE | 80 | 0.934 | 0.001103 | 542 |
| ENSP00000379625 | MYD88 | 519 | 0.953 | 0.007159 | 983 |
| ENSP00000278568 | PAK1 | 497 | 0.954 | 0.006855 | 932 |
| ENSP00000288986 | NCK1 | 496 | 0.954 | 0.006842 | 997 |
| ENSP00000302967 | HDAC3 | 349 | 0.955 | 0.004814 | 991 |
| ENSP00000315702 | MOB4 | 31 | 0.96 | 0.000428 | 394 |
| ENSP00000362820 | SRSF3 | 10 | 0.961 | 0.000138 | 188 |
| ENSP00000358022 | MCL1 | 690 | 0.963 | 0.009518 | 999 |
| ENSP00000262238 | YY1 | 71 | 0.964 | 0.000979 | 988 |
| ENSP00000242577 | DYNLL1 | 31 | 0.97 | 0.000428 | 663 |
| ENSP00000310127 | IRF3 | 175 | 0.973 | 0.002414 | 865 |
| ENSP00000266970 | CDK2 | 757 | 0.975 | 0.010442 | 988 |
| ENSP00000264033 | CBL | 13245 | 0.985 | 0.182695 | 999 |
| ENSP00000351997 | MAP2K6 | 128 | 0.999 | 0.001766 | 999 |
